# Safety and Preliminary Clinical Outcomes of Silver Ion-Doped Calcium Phosphate-Coated Orthopedic Implants: A Prospective Pilot Study

**DOI:** 10.3390/jfb17100517

**Published:** 2026-10-15

**Authors:** Hünkar Çağdaş Bayrak, Bünyamin Yücel, Ayşe Aydan Köse, Feza Korkusuz, Yavuz Akalın, Nusret Köse

**Affiliations:** 1 Department of Orthopedics and Traumatology, Health Sciences University Bursa Yüksek Ihtisas Training and Research Hospital, Bursa 16130, Türkiye; dryakalin@yahoo.com; 2 Department of Orthopedics and Traumatology, Faculty of Medicine, Eskisehir Osmangazi University, Eskisehir 26040, Türkiye; drbunyaminyucel@gmail.com (B.Y.); drnkose@gmail.com (N.K.); 3 Department of Plastic and Reconstructive Surgery, Faculty of Medicine, Eskisehir Osmangazi University, Eskisehir 26040, Türkiye; aydan.kose@gmail.com; 4 Department of Sports Medicine, Faculty of Medicine, Hacettepe University, Ankara 06230, Türkiye; feza.korkusuz@gmail.com

**Keywords:** silver, calcium phosphate, orthopedic implant, implant-related infection, ceramic coating, prospective study, pilot study

## Abstract

**Background:** Implant-related infections remain a major cause of failure in orthopedic surgery, partly because biofilm formation on implant surfaces limits conventional infection-control strategies. This prospective pilot study evaluated the short-term safety and preliminary anti-infective performance of silver ion-doped calcium phosphate-coated orthopedic implants. **Methods:** Thirty patients undergoing surgery for lower-extremity fractures received 107 coated implants, including 91 external fixator pins in 14 patients, 10 proximal femoral nails, and 6 partial hip arthroplasty. The primary endpoint was pin-tract infection, while secondary endpoints included liver and kidney function, inflammatory markers, and serum silver concentrations. **Results:** Pin-tract infection occurred in 4 of 91 pins (4.39%), involving 2 of 14 patients (14.3%) during follow-up. In one patient, the infection resolved, whereas in the other it persisted until pin removal, corresponding to 1 of 14 patients (7.1%) at final outcome. No infections occurred after proximal femoral nailing, whereas two infections developed after partial hip arthroplasties and were successfully treated with implant-retaining debridement and antibiotics. Liver and kidney function remained stable, inflammatory markers decreased over time, and serum silver concentrations remained below the lower detection limit (<0.3506 µg/L). **Conclusions:** The coating demonstrated a favorable short-term safety profile without evidence of systemic silver toxicity. Its anti-infective effectiveness requires confirmation in larger controlled studies.

## 1. Introduction

The development of advanced materials for orthopedic implants has made significant progress in the search for biomaterials with functional surfaces that enhance biocompatibility, prevent implant corrosion, and reduce bacterial colonization. Implant-related infections (periprosthetic infections) remain one of the most serious complications, leading to implant failure, prolonged hospitalization, repeated surgeries, and increased costs [1,2]. The incidence of implantable device-related musculoskeletal infections or implant-related osteomyelitis (IRO) has been reported to be between 0.5% and 5%, and these infections are associated with significant morbidity and disability [3]. The incidence increases up to 15% after open fracture fixation despite antibiotic prophylaxis [4]. A recent study [5] reported an incidence of 26.4%, with *Staphylococcus aureus* identified as the primary infectious agent. Patients who developed hip or knee arthroplasty infections remained in hospital 2.5–3 times longer and incurred hospital costs almost three times higher compared with uninfected patients [6].

Biofilm-forming bacteria can attach to implant surfaces and develop antimicrobial resistance despite optimal sterile operating conditions and systemic antibiotic prophylaxis. Coating implant surfaces to prevent bacterial adhesion has gained increasing attention, particularly through biomaterials designed to provide local antibacterial release [7]. Silver has emerged as a promising option for implant surface coating because of its broad-spectrum antimicrobial activity against Gram-positive and Gram-negative bacteria. Silver ions disrupt bacterial membranes, interfere with DNA replication, and induce cell death by generating reactive oxygen species. Silver ions exert antimicrobial effects through multiple cellular targets; however, bacterial adaptation and silver-resistance mechanisms have been reported. Therefore, the potential for resistance should not be considered negligible, particularly under prolonged or sub-inhibitory silver exposure [8].

Various silver-coated orthopedic implants are currently used in clinical practice. These include electrochemically silver-coated prostheses and megaprostheses with thin silver coatings applied by physical vapour deposition. Silver-coated intramedullary nails with covalently attached silver on the titanium surface have been used in open long-bone fractures. Plates for fracture fixation have also been coated with silver. Several silver-based surface modification strategies have been investigated to achieve antibacterial activity while maintaining or promoting osseointegration. These include incorporation of silver into bone-compatible ceramics such as hydroxyapatite and tricalcium phosphate, as well as silver-containing modifications of titanium surfaces [8,9,10].

Pure metallic silver coatings or simple silver layers without ceramic incorporation have several shortcomings compared to silver-ceramic coatings (e.g., silver embedded in matrices like hydroxyapatite [HA], or other ceramic composites). These disadvantages stem from differences in silver release kinetics, mechanical stability, biocompatibility, and interaction with host tissues/bacteria. Uncontrolled or burst-like silver ion release leading to local toxicity, poor mechanical durability, and risk of delamination or wear, higher risk of systemic accumulation and side effects (e.g., argyria), and suboptimal balance between antibacterial efficacy and osseointegration are main concerns [9,11,12,13].

This study evaluated the clinical use of orthopedic implants coated with silver ion-doped calcium phosphate ceramics prepared by electrospray. Electrospray generates homogeneous nanoscale coatings at low cost and can be used for complex geometries, offering a biologically suitable alternative to conventional plasma spray, chemical vapour deposition, or physical vapour deposition techniques [14].

The aim of this study was to evaluate the clinical performance, infection-related outcomes, and systemic safety of orthopedic implants coated with electrospray-synthesized silver ion-doped calcium phosphate ceramics. We hypothesized that silver ion-doped calcium phosphate-coated implants would demonstrate acceptable clinical safety and favorable preliminary infection-related outcomes during short-term clinical follow-up.

## 2. Materials and Methods

### 2.1. Study Design and Participants

This prospective observational clinical study was conducted between 2018 and 2020 at the Department of Orthopedics and Traumatology, Eskişehir Osmangazi University Faculty of Medicine. The study protocol was approved by the Eskişehir Osmangazi University Clinical Research Ethics Committee (Approval No: [08], Date: 23 November 2015), and all procedures were performed in accordance with the principles of the Declaration of Helsinki. Written informed consent was obtained from all participants. ClinicalTrials.gov ID: NCT03343288. The study was supported by the Scientific and Technological Research Council of Turkey (TÜBITAK, Project No: 315S101).

Inclusion criteria were age ≥18 years, absence of systemic infection or immunodeficiency, no history of allergy to silver, and provision of written informed consent. Patients with malignancy, diabetes mellitus, or advanced renal or hepatic failure were excluded.

**Figure 1:**
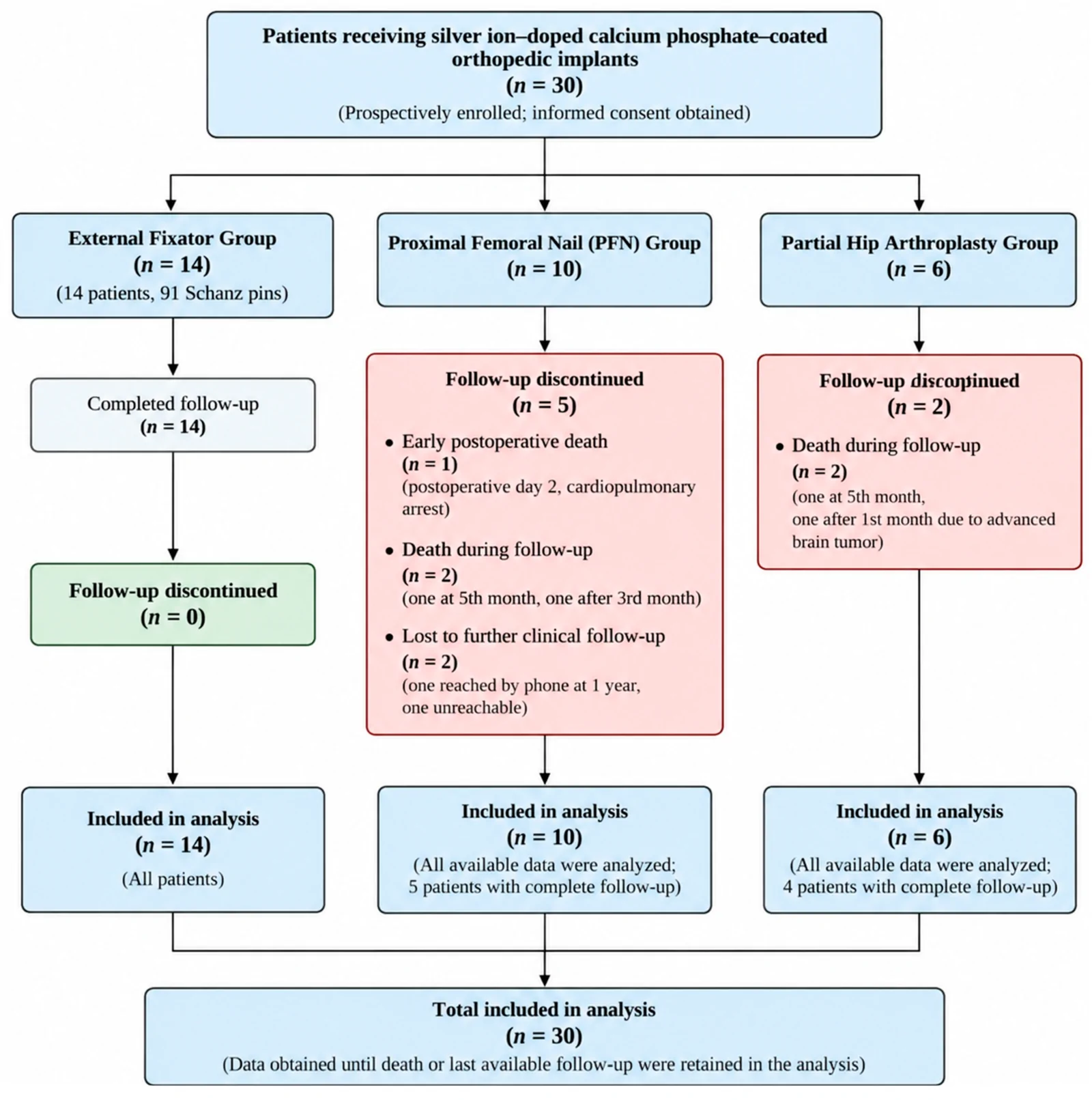
Study flow diagram showing patient enrollment, follow-up status, and inclusion in the final analysis.

A total of 30 adult patients requiring orthopedic implants for lower-extremity fractures were enrolled. Patients were assigned to three groups according to the planned surgical procedure: external fixator (*n* = 14), proximal femoral nail (PFN, *n* = 10), and partial hip arthroplasty (PHA, *n* = 6). All implants were coated with silver ion-doped calcium phosphate using the electrospray technique (Figure 1). The three implant groups represented clinically distinct orthopedic applications with different anatomical environments, implant–tissue interfaces, surface areas, and mechanisms of infection. Accordingly, clinical outcomes were evaluated descriptively within each implant group, and no direct comparison of infection outcomes between the three groups was intended.

### 2.2. Implant Coating and Preclinical Testing

The substrate material differed according to implant type. The external-fixator pins were manufactured from 316L stainless steel (Tıpmed, İzmir, Türkiye), whereas the proximal femoral nails and partial hip arthroplasty components were manufactured from Ti6Al4V titanium alloy (FX, İzmir, Türkiye).

The antibacterial surface modification consisted of a two-layer coating comprising a 6P57 bioglass layer and a silver ion-doped calcium phosphate/HAP-based ceramic layer, applied to the metallic substrates via electrospray deposition.(TÜBİTAK Project No: 315S101).

The electrospray coating procedure enables the formation of homogeneous nanoscale coatings, even on complex implant geometries, while providing both osteoconductive and antibacterial properties [15,16].

**Figure 2:**
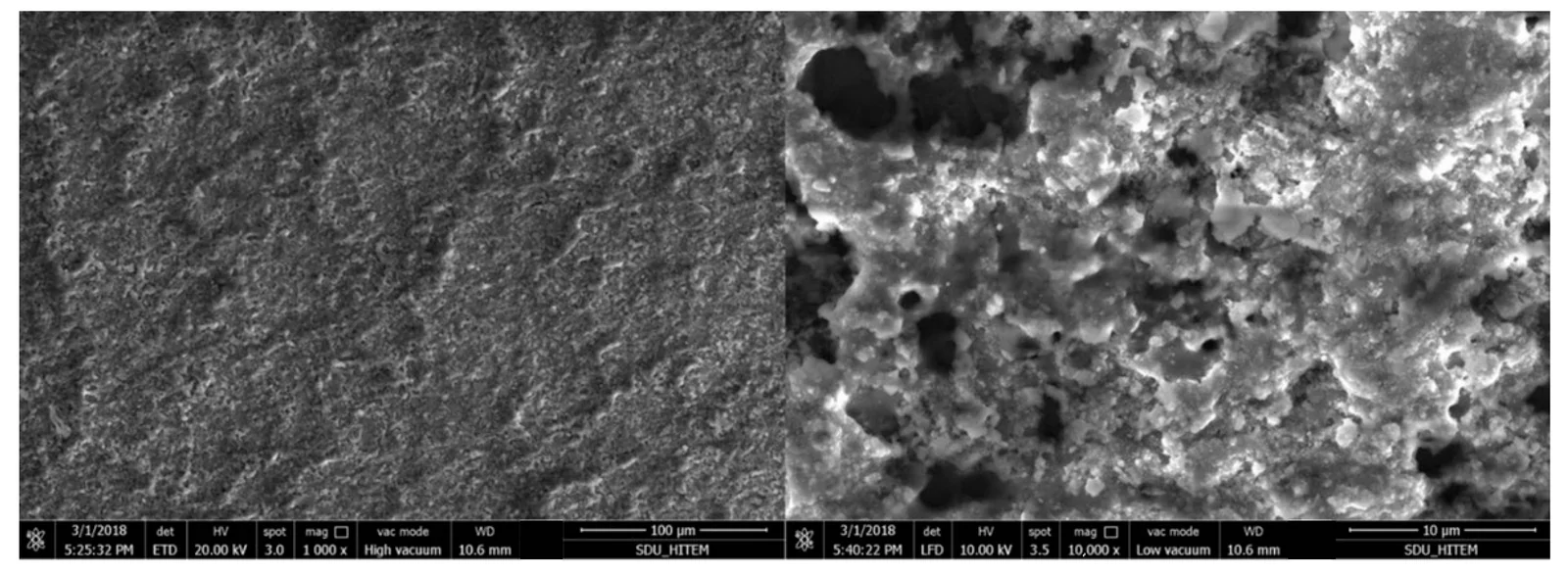
SEM image of the electrospray-coated surface, showing homogeneous nanoscale morphology after mechanical testing.

**Figure 3:**
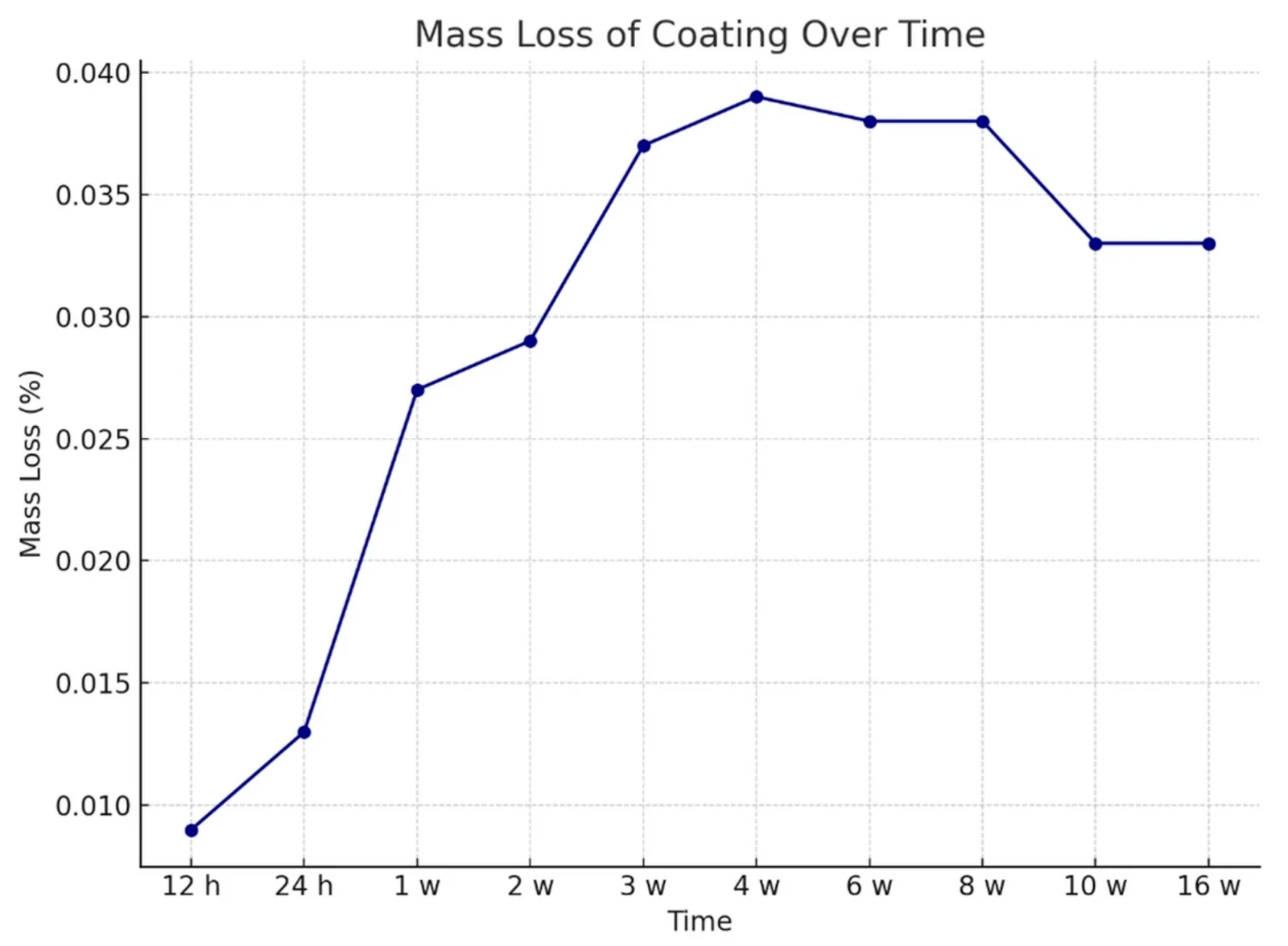
Mass loss profile of the silver ion-doped calcium phosphate coating during static dissolution testing in plasma at 37 °C over 16 weeks. The coating exhibited minimal weight change (<0.1%), indicating limited mass loss and supporting coating stability under the investigated dissolution conditions.

**Figure 4:**
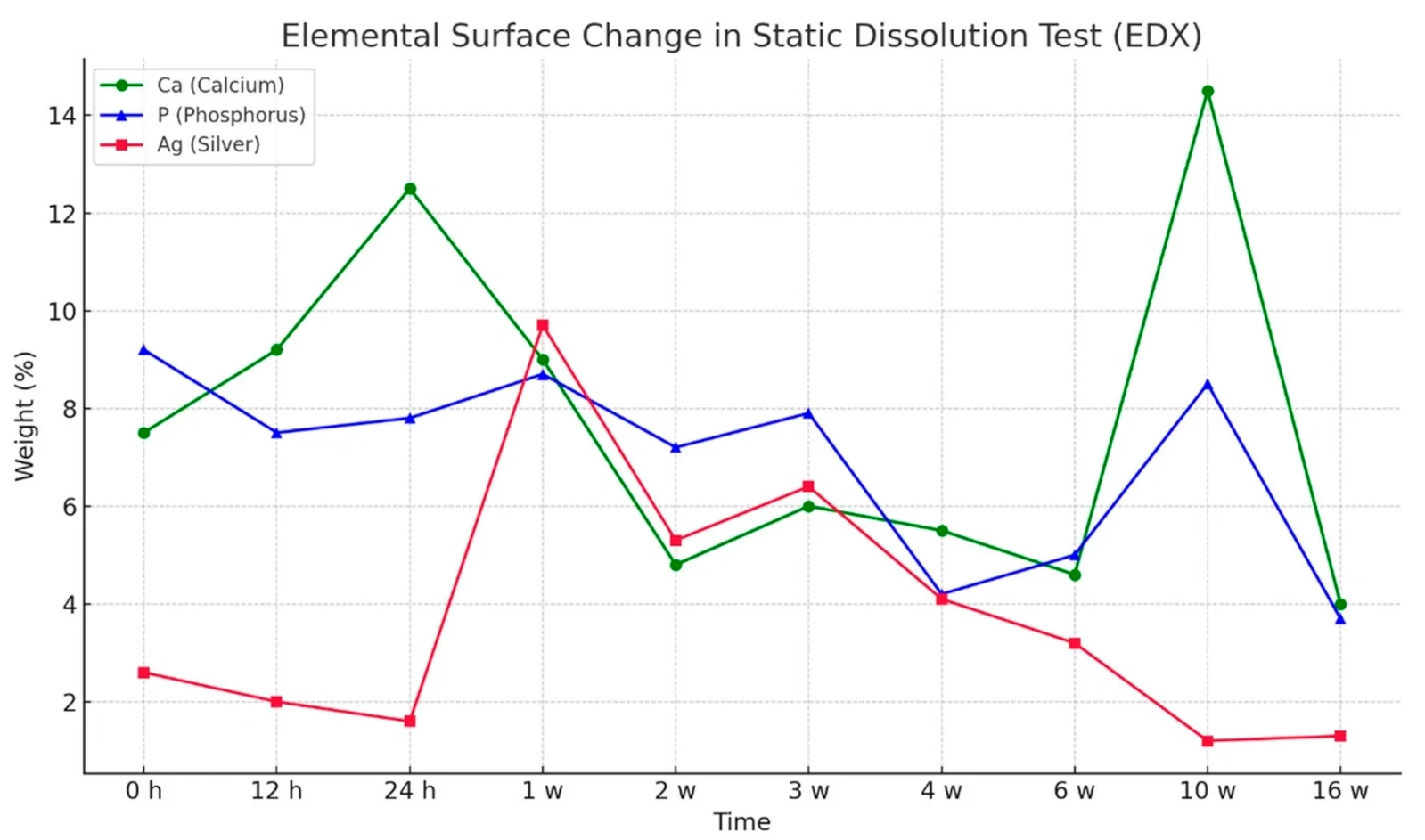
Energy-dispersive X-ray (EDX) elemental analysis of calcium (Ca), phosphorus (P), and silver (Ag) on the implant surface during static dissolution testing in plasma at 37 °C. Despite fluctuations over time, all three elements remained detectable on the surface throughout the 16-week observation period, indicating persistence of the coating constituents. EDX provides information on surface elemental composition and does not directly quantify ion release into the surrounding medium.

**Figure 5:**
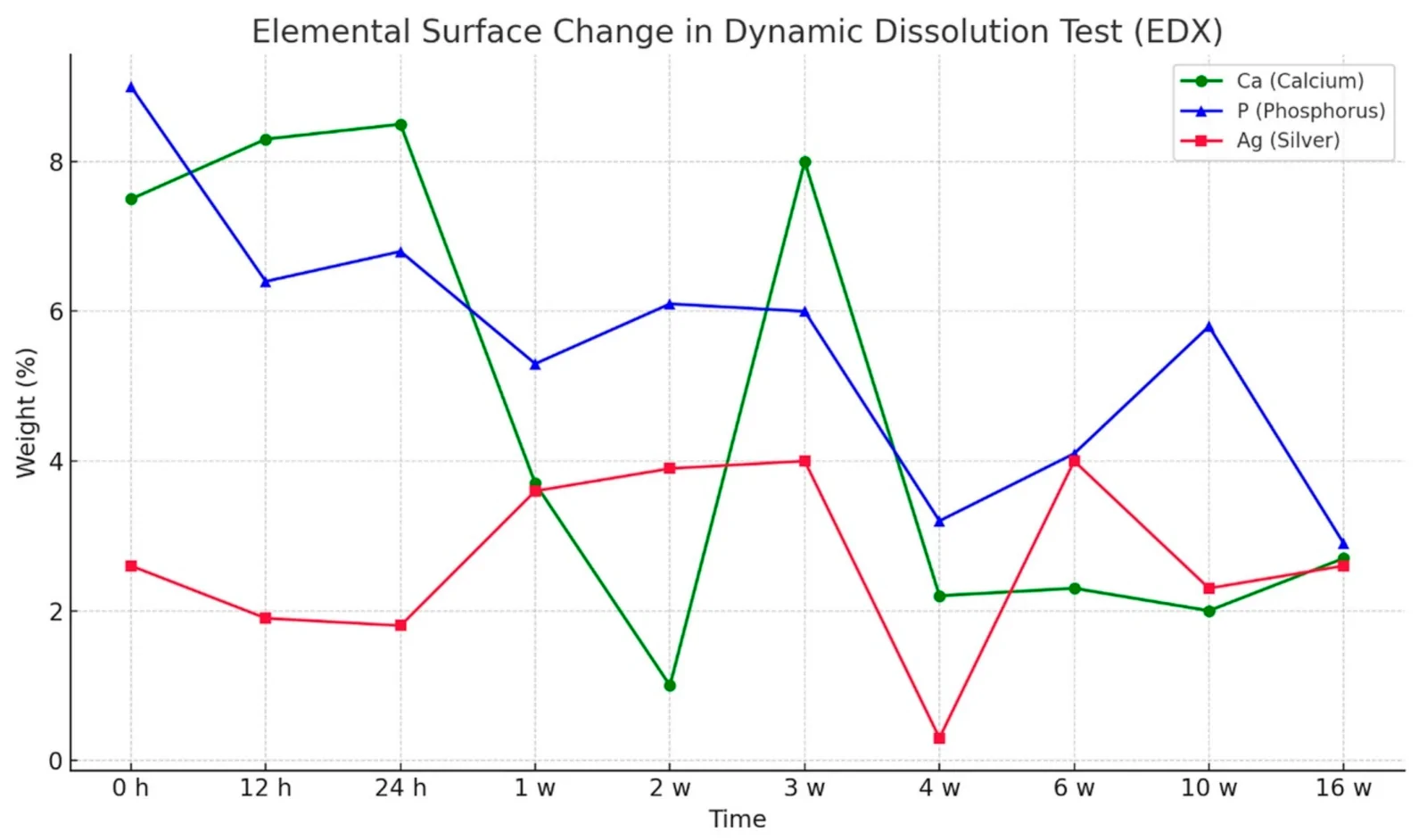
Energy-dispersive X-ray (EDX) elemental analysis of calcium (Ca), phosphorus (P), and silver (Ag) on the implant surface during dynamic dissolution testing in plasma at 37 °C. Elemental signals remained detectable for up to 16 weeks, supporting persistence of the coating constituents under dynamic conditions. Silver ion release into the surrounding medium was not directly quantified in this experiment.

Before clinical use, the coating was characterized by scanning electron microscopy and energy-dispersive X-ray spectroscopy (EDX). Scanning electron microscopy demonstrated a homogeneous and continuous coating morphology (Figure 2). Cross-sectional SEM examination demonstrated an approximate coating thickness of 10 µm. Mechanical integrity of the coating was additionally evaluated using a progressive micro-scratch test on coated external-fixator pins. The applied load was increased from 0.05 to 5 N over a 4 mm scratch length using a 100 µm Rockwell indenter. An abrupt change in the friction and penetration curves occurred at approximately 1 N, which was defined as the critical load (Lc) for the onset of coating damage. EDX analysis of the heat-treated coating demonstrated the presence of Ag within the surface elemental composition, with an approximate Ag content of 3.5 wt%. The Ca/P ratio of the pH 5.5 calcium-phosphate powder used for coating preparation was 1.63; however, a separate quantitative Ca/P ratio of the final implant coating was not determined. The same coating formulation and principal electrospray parameters were used for all three implant types, while coating duration was adjusted according to implant geometry and dimensions. Static and dynamic dissolution experiments were subsequently performed in human plasma at 37 °C for up to 16 weeks to evaluate coating stability and dissolution behavior. Mass loss remained minimal (<0.1%) during the static dissolution experiment (Figure 3). Surface EDX analyses performed during the static and dynamic experiments demonstrated the continued presence of calcium, phosphorus, and silver on the implant surface throughout the study period (Figure 4 and Figure 5, respectively). These findings indicate persistence of the Ag-containing coating during prolonged exposure to plasma. However, although changes in surface Ag content were monitored by EDX, Ag concentration in the surrounding dissolution medium was not directly quantified; therefore, specific local silver ion release kinetics could not be determined.

### 2.3. Clinical Application and Follow-Up

All patients received perioperative antibiotic prophylaxis with a first-generation cephalosporin administered 30–60 min before incision and continued for 24 h postoperatively unless contraindicated. Coated implants were implanted using standard surgical techniques according to the indication. Patients were scheduled for 12 months of follow-up. The follow-up protocol consisted of weekly visits during the first month (four visits) and monthly visits from the second to the twelfth month. For patients who died or discontinued follow-up before 12 months, all available data up to the last completed assessment were retained, and missing subsequent observations were not imputed.

At each visit, the surgical wound, pin insertion sites, and systemic signs of infection were assessed. Pin-tract infection was assessed clinically based on local inflammatory/infectious findings at the pin site, including erythema and/or purulent discharge. Pin-site assessments were performed consistently by the same investigator throughout follow-up, with consultation of the senior investigator when clarification was required. Microbiological confirmation was not required for the clinical diagnosis, although pin-site cultures were obtained during follow-up. No standardized pin-tract infection grading system was applied. In the partial hip arthroplasty group, PJI was diagnosed when a draining sinus tract communicating with the implant was present. Microbiological cultures were obtained when clinically indicated. Laboratory monitoring included complete blood count, liver function tests (AST, ALT), renal function tests (BUN, creatinine), and inflammatory markers (CRP, ESR). Serum silver concentrations were measured together with the other laboratory parameters at all available follow-up time points using ICP-MS (Agilent Technologies, Santa Clara, CA, USA 8800 Triple Quadrupole) at Anadolu University laboratories. The analytical measurement range was 2–2694 µg/L, and the lower detection limit was 0.3506 µg/L. All available serum silver measurements were below the detection limit and were therefore reported as <0.3506 µg/L. A separate laboratory reference interval was not available. Blood samples for silver analysis were obtained by standard venipuncture from the cubital vein, remote from the surgical site.

In the external fixator group, microbiological cultures were obtained from pin insertion sites during follow-up. At the time of pin removal, a deep-tissue specimen was additionally obtained from each pin tract for microbiological evaluation. The microbiological distribution reported in this study was based on these post-removal deep-tissue cultures, with one result assigned to each pin.

**Figure 6:**
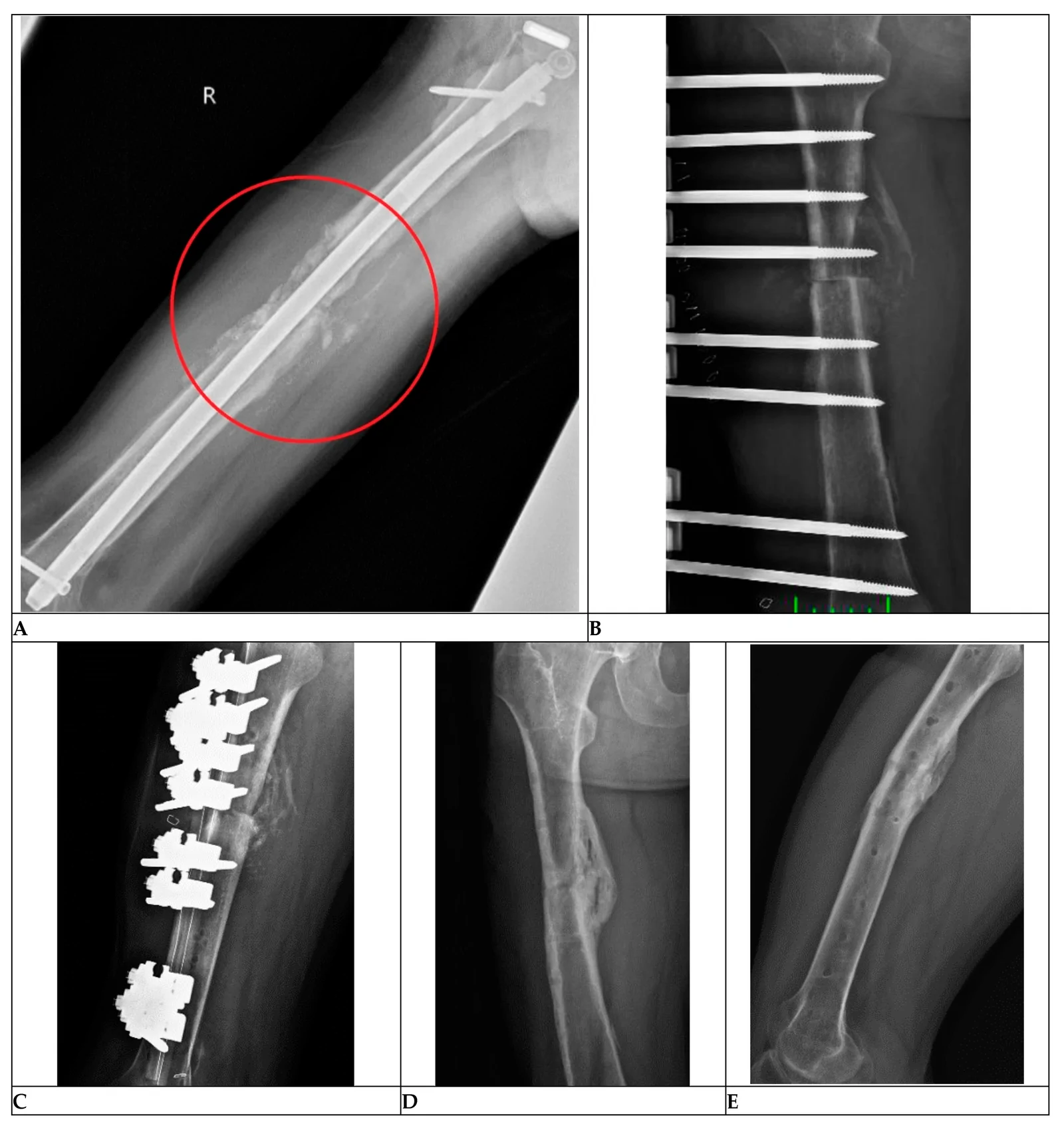
Clinical case of an 18-year-old male with femoral nonunion and chronic osteomyelitis treated with an external fixator using silver ion-doped calcium phosphate-coated pins. Images show (**A**) preoperative radiograph showing the osteomyelitic area highlighted by a red circle. R indicates the right side. (**B**,**C**) the immediate postoperative view, and (**D**,**E**) the 6-month follow-up, demonstrating maintained fixation and absence of pin-tract infection.

**Figure 7:**
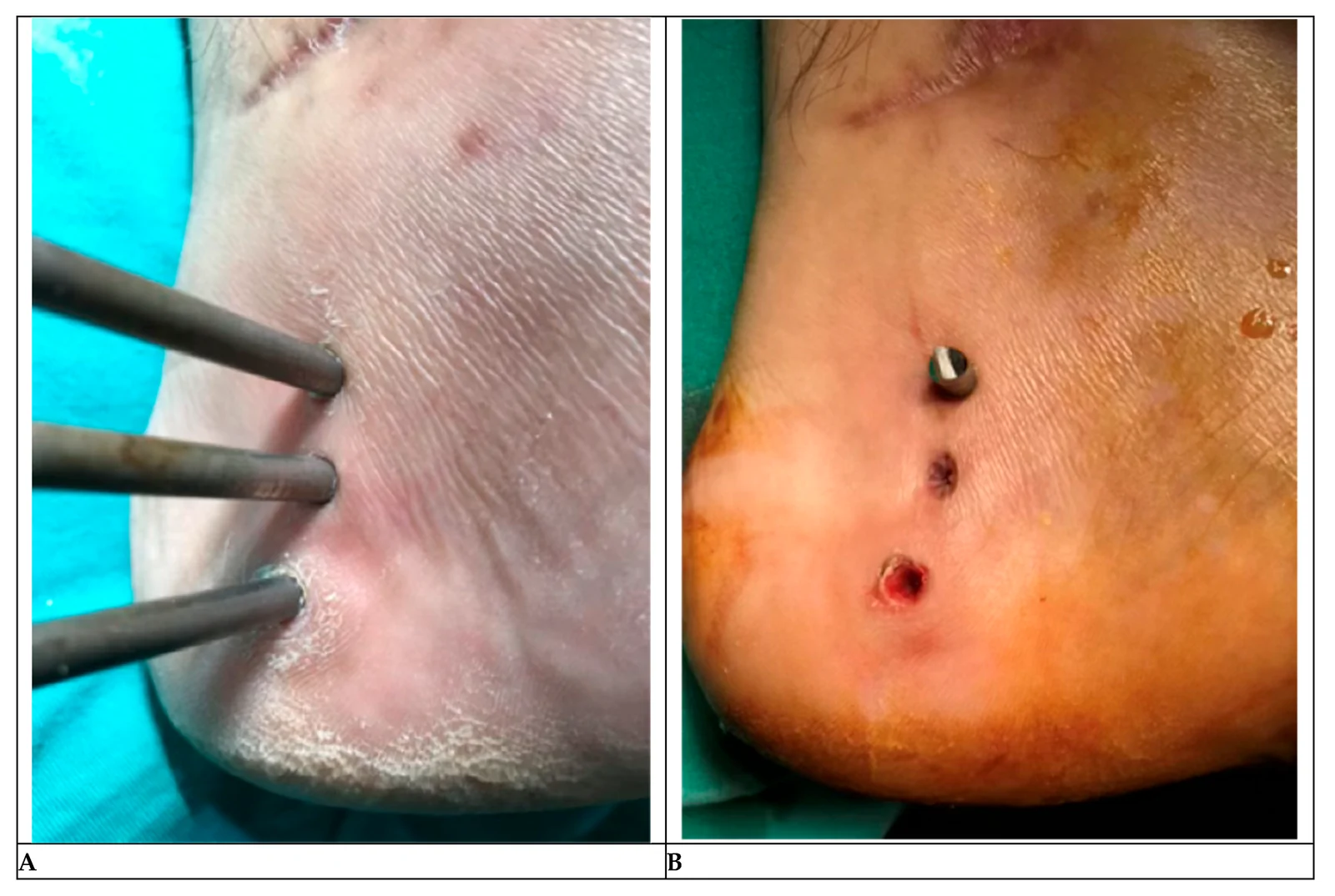
Clinical case of a 45-year-old male with open fracture dislocation of the ankle treated with an external fixator using silver ion-doped calcium phosphate–coated pins. (**A**) Clinical appearance of the pin sites at 6 weeks, without signs of local inflammation or infection. (**B**) Intraoperative appearance at the time of pin removal.

Representative clinical cases are shown in Figure 6 and Figure 7.

### 2.4. Statistical Analysis

Descriptive statistics were reported as mean ± standard deviation (SD) and interquartile range (IQR) for continuous variables and as counts with percentages for categorical variables. Pin-tract infection was reported descriptively at both the pin and patient levels. Because multiple pins were nested within individual patients, pin-level observations were not considered statistically independent for inferential analysis. The 27.4% pin-tract infection rate reported in the literature was used only as a descriptive historical reference and was not subjected to formal hypothesis testing. All analyses were performed using SPSS software (version 27.0; IBM Corp., Armonk, NY, USA).

## 3. Results

A total of 30 patients were included in this study: 14 treated with external fixators (91 pins), 10 with proximal femoral nails, and six with partial hip arthroplasty. The cohort’s mean age was 56.13 ± 19.95 years, with 18 men and 12 women. Demographic and baseline characteristics are presented in Table 1.

In the external fixator group, pin-tract infection was observed in 4 of 91 pins (4.39%), involving 2 of 14 patients (14.3%). Two pins were affected in each patient. In one patient, the infection resolved during follow-up, whereas in the other, clinical signs persisted until pin removal, corresponding to 1 of 14 patients (7.1%) at final outcome. No infections were observed in patients treated with proximal femoral nails. In the partial hip arthroplasty group, two of six patients developed early postoperative PJI. In the first patient, hip dislocation was detected at the 1-week postoperative visit and a second operation was performed. Purulent wound drainage developed after this second procedure. *Acinetobacter* was isolated from the wound culture, and debridement demonstrated extension of the infectious process to the implant. The implant was retained, three sessions of vacuum-assisted wound therapy were performed, and systemic antibiotic treatment with tigecycline followed by meropenem and colistin was administered. The wound subsequently healed, with sutures removed 3 weeks after the second operation. In the second patient, persistent wound drainage developed during the first 2 postoperative weeks. Debridement demonstrated infection extending to the implant; the implant was retained, three sessions of vacuum-assisted wound therapy were performed, and tigecycline was administered. Complete wound healing was documented at the follow-up period.

The distribution of pin outcomes is summarised in Table 2.

Liver (AST, ALT) and kidney (BUN, creatinine) function tests remained stable during the available follow-up, while CRP and ESR showed postoperative variation, with elevations at some early time points followed by an overall decreasing trend during subsequent follow-up. None of the patients developed clinical signs of systemic toxicity, and serum silver concentrations remained below the lower detection limit (<0.3506 µg/L). Detailed laboratory results are provided in the Supplementary Materials.

Microbiological evaluation of the external fixator group was based on deep-tissue specimens obtained from the pin tract after pin removal. Each of the 91 pins contributed a single culture result. Of these, 54 cultures (59.4%) were positive, whereas 37 (40.6%) showed no bacterial growth. *Staphylococcus epidermidis* was the most frequently isolated microorganism (20/91, 21.9%), followed by normal skin flora and *Staphylococcus aureus* (9/91 each, 9.8%). Other isolates included *Staphylococcus lugdunensis* (6/91, 6.6%), *Klebsiella* spp. (3/91, 3.3%), and other microorganisms (7/91, 7.6%). Repeated isolates from the same pin were therefore not counted independently. Detailed microbiological findings are presented in the Supplementary Materials.

## 4. Discussion

In this prospective study, the clinical performance of silver ion-doped calcium phosphate ceramic-coated implants was evaluated with respect to infection-related outcomes and systemic safety. In the external fixator group, pin-tract infection was observed in 4 of 91 pins (4.39%), involving 2 of 14 patients (14.3%) during follow-up. One of these infections resolved, whereas the other persisted until pin removal, corresponding to 1 of 14 patients (7.1%) at final outcome. However, due to the absence of a control group and the limited sample size, no conclusions regarding comparative effectiveness can be drawn. The PFN and partial hip arthroplasty cohorts were small and represented clinically distinct implant applications; therefore, findings from these groups should be interpreted primarily as preliminary feasibility and safety observations rather than evidence of anti-infective efficacy. In particular, successful treatment of the two PJI cases in the partial hip arthroplasty group with implant-retaining debridement and antibiotic therapy should not be interpreted as evidence of antibacterial activity of the coating.

Additionally, microbiological cultures obtained from pin sites showed a positivity rate of 59.4%, whereas the clinical infection rate remained low. Most isolates consisted of skin flora microorganisms, with Staphylococcus epidermidis (21.9%) being the most frequently detected pathogen. The observed discrepancy between culture positivity and clinically diagnosed infection indicates that microbiological culture positivity at the pin site did not necessarily correspond to clinically apparent infection during the follow-up period.

From a systemic safety perspective, liver and kidney function tests remained stable during follow-up, while inflammatory markers showed postoperative variation followed by an overall decreasing trend; serum silver concentrations remained below the lower detection limit (<0.3506 µg/L), and no signs of systemic toxicity were observed. Within the duration of follow-up, these findings suggest that the coating can be used without evident systemic adverse effects across the investigated implant types.

In the literature, infection rates associated with orthopedic implants are reported to be highly variable and generally elevated. These rates differ depending on the surgical technique, patient characteristics, and implant type [17]. One of the major reasons for the limited effectiveness of systemic antibiotics is biofilm formation on implant surfaces [18,19]. Consequently, surface modifications have emerged as an essential strategy in modern infection control [20,21].

External fixators are among the applications most frequently associated with implant-related infections. In a meta-analysis of 150 studies involving 6130 patients, Iobst and Liu reported an average pin-tract infection rate of 27.4% [22]. Similarly, Mahan et al. prospectively evaluated 214 pins in 42 patients and reported inflammation in 41.6% and culture positivity in 74.8% of cases, with *Staphylococcus epidermidis* being the most frequently isolated microorganism (90.6%) [23]. In our study, culture positivity was observed in 59.4% of pin sites, with most isolates belonging to skin flora microorganisms, while *Staphylococcus epidermidis* (21.9%) was the most common pathogen identified. Camathias et al. reported secretion rates of 35–36%, and Ahlborg et al. reported a pin infection rate of 21% in a series of 314 cases [24,25]. The pin-tract infection rate in our series was 4.39% at the pin level, involving 2 of 14 patients (14.3%) during follow-up; clinical signs persisted until pin removal in 1 of 14 patients (7.1%) at final outcome. The pin-level rate was numerically lower than the 27.4% historical rate reported by Iobst and Liu; however, this comparison is descriptive only. Because multiple pins were nested within individual patients, individual pins cannot be considered statistically independent observations. Furthermore, the lack of clinical infection despite culture positivity suggests that culture positivity did not necessarily translate into clinically apparent infection during the follow-up period. Differences in the definitions and grading systems used for pin-tract infection across previous studies limit direct comparability with the historical infection rates cited above; therefore, this historical comparison should be interpreted cautiously.

Intramedullary nails are also associated with surgical site infections, with reported infection rates ranging from 0.9% to 23%, notably higher in open fractures and high-energy injuries [26]. In a prospective study of 231 patients (99 femur, 122 tibia), Oliveira et al. reported an infection rate of 11.6% [27]. In our series of ten patients treated with silver ion-doped coated nails, no infections were observed. Although no infections were observed in this small cohort, the limited number of patients precludes any conclusions regarding the coating’s effectiveness.

Partial hip arthroplasty is another setting in which periprosthetic joint infection remains a serious complication. Bozic et al. identified infection as the third most common cause of revision hip arthroplasty (14.7%) and the most frequent cause of failure after total knee arthroplasty (25.2%) [28]. In a large-scale study, Guillon et al. reported a periprosthetic joint infection incidence of 2.2% following partial hip arthroplasty [29]. In our series, two of six patients developed early postoperative PJI. Both were treated with debridement, implant retention, vacuum-assisted wound therapy, and systemic antibiotics. One case was microbiologically confirmed with *Acinetobacter*, while the second was diagnosed clinically and intraoperatively based on infection extending to the implant. Both infections ultimately resolved without implant removal. Although reported success rates for DAIR in hip and knee PJI vary in the literature, with rates of approximately 50–60% reported in a recent systematic review [30], the successful treatment of both cases in our small cohort should be regarded only as a descriptive clinical observation and should not be interpreted as evidence that the coating contributed to infection control or anti-infective efficacy.

The antibacterial properties of silver ions may provide a biological rationale for the favorable infection-related observations reported in this study. Silver exhibits broad-spectrum activity against both Gram-positive and Gram-negative bacteria. It binds to the bacterial cell wall and membrane, disrupting permeability, interfering with DNA replication, and inducing the production of reactive oxygen species, ultimately leading to bacterial cell death. Although the multiple antimicrobial mechanisms of silver may reduce the likelihood of resistance compared with single-target antimicrobial agents, bacterial adaptation and silver-resistance mechanisms have been described. This possibility may be particularly relevant during prolonged exposure to sub-inhibitory silver concentrations and should be considered when developing long-term silver-based implant coatings [31,32].

Silver coatings are most established in high-risk scenarios (e.g., tumor megaprostheses, infection revisions), with commercial examples such as MUTARS^®^, Agluna^®^, PorAg^®^, and AG-PROTEX. Different forms of silver are incorporated into orthopedic implant surfaces and vary in deposition method, release profile, durability, antimicrobial efficacy, and biocompatibility, with potential cytotoxicity to host cells such as osteoblasts or fibroblasts at higher concentrations. Pure or nearly pure elemental silver layers are applied via electrochemical deposition, physical vapor deposition (PVD), magnetron sputtering, or similar techniques. Thick electrochemical coatings, such as MUTARS^®^ megaprostheses containing approximately 0.7–1.2 g silver per implant, and thinner PVD coatings, such as PorAg^®^ megaprostheses containing up to 0.33 g silver, are examples. Higher silver loads may be associated with argyria or systemic silver elevation [33]. Such high-silver-load coatings may also be less suitable for bone-contact surfaces because excessive Ag exposure may interfere with osteoconductivity and impair osteogenic differentiation and mineralization. Silver in nanoscale form, embedded in matrices such as hydroxyapatite or polymers, or applied via plasma immersion ion implantation, sputtering, or doping, is less common in clinical orthopedic prostheses than metallic silver coatings. Nanoparticles may exert biological effects beyond ion release and may also contribute to cytotoxicity depending on dose and exposure conditions. Silver ion-based coatings, in which silver is incorporated into matrices such as hydroxyapatite or tricalcium phosphate, have shown antimicrobial activity and may reduce bacterial adhesion depending on coating composition and release characteristics. The biological response to silver-containing coatings depends on silver species, local concentration, exposure duration, biological medium, and the characteristics of the exposed bacterial and host cells. Therefore, optimisation of silver-containing coatings requires balancing antimicrobial activity against potential local cytotoxicity. Ongoing research focuses on optimising silver-containing coating systems to achieve an appropriate balance between antimicrobial activity and biocompatibility, while also integrating osteopromotive components and developing smart or responsive surface technologies [9,11,12,13,34].

Calcium phosphate, and particularly hydroxyapatite, is frequently used in orthopedic implants owing to its high biocompatibility with bone tissue. Hydroxyapatite coatings promote bone cell adhesion, enhance osseointegration, and strengthen the primary stability of the implant. However, pure hydroxyapatite coatings have limited antimicrobial activity and are insufficient to protect against infection on their own [35].

The combination of silver and hydroxyapatite provides a complementary biological rationale: silver may contribute antimicrobial effects, while hydroxyapatite supports osteogenic potential and bone integration. The biological effects of silver-containing coatings depend on the amount and kinetics of silver released from the surface. Excessive local exposure may lead to toxicity and osteoblast inhibition, whereas insufficient exposure may provide inadequate antibacterial activity [36,37,38,39]. In the present study, EDX analysis demonstrated that Ag, Ca, and P remained detectable on the implant surface for up to 16 weeks during the dissolution experiments, supporting persistence of the Ag-containing calcium phosphate coating during prolonged exposure to plasma. However, EDX reflects the elemental composition of the residual coating and should not be interpreted as a direct measurement of silver-ion release kinetics. Although ICP-OES analysis of the surrounding plasma demonstrated changes in several dissolved elements related to coating–medium interactions, Ag concentration in the dissolution medium was not quantified. Furthermore, total silver mass per implant, surface silver loading expressed as mass per unit area, and local Ag concentrations at the implant–tissue interface were not directly determined. Therefore, while systemic silver exposure remained very low and no clinically apparent systemic toxicity was observed, the present data do not permit determination of local Ag-release kinetics or a direct concentration–response relationship between local silver exposure, antimicrobial efficacy, and cellular cytotoxicity.

The electrospray coating technique also played a critical role in this outcome. This method atomises charged droplets under a high-voltage electric field and deposits them uniformly onto the implant surface, resulting in homogeneous nanoscale coatings. Its ability to cover complex geometries and its applicability at low temperatures make it particularly suitable for biological agents and metal substrates without causing thermal degradation. With these features, electrospray offers a more controlled and biologically favourable alternative compared with conventional methods such as plasma spraying, chemical vapour deposition, or physical vapour deposition [40,41].

The most important limitations of this study are the relatively small sample size and the heterogeneity among treatment groups. In particular, the small number of patients in the proximal femoral nail and partial hip arthroplasty groups precludes firm conclusions about implant-specific infection rates and limits interpretation of efficacy outcomes. Therefore, findings related to these implant types should primarily be considered in the context of feasibility and short-term safety rather than definitive anti-infective effectiveness. Additionally, the three implant groups differed substantially in anatomical location, implant surface area, mechanical environment, tissue contact, and potential mechanisms of infection; therefore, infection-related outcomes should not be directly compared across implant groups. Furthermore, pin-tract infections were defined based on clinical findings, and no standardized pin-tract infection grading system was used, representing an additional limitation. As this investigation was designed as an early-phase prospective observational study evaluating the first clinical use of this specific electrospray-deposited silver ion-doped calcium phosphate coating system, the absence of a concurrent randomized control group using uncoated implants represents an inherent limitation of the study design. The follow-up period was limited to 12 months, precluding definitive conclusions regarding long-term efficacy, coating degradation, and biocompatibility. Importantly, the study design did not allow direct assessment of biofilm formation, bacterial adhesion, or implant-surface colonization. Therefore, no conclusions can be made regarding the coating’s effect on biofilm prevention. Silver ion release into the surrounding medium, surface silver loading, local tissue silver concentrations, and direct cellular cytotoxicity were not quantified in the present study. Therefore, no direct concentration–response relationship between local silver exposure, antimicrobial activity, and cytotoxicity can be established. Furthermore, several patients in the external-fixator group had pre-existing chronic osteomyelitis or locally infected conditions; therefore, differentiation between pin-site infection and inflammatory or infectious findings related to the underlying condition may not have been unequivocal in all cases.

Nevertheless, the study also has several notable strengths. Most importantly, this study reports prospective clinical experience with this specific electrospray-deposited silver ion-doped calcium phosphate coating system in humans. The integration of preclinical laboratory findings with clinical data demonstrates consistency between biological mechanisms and clinical outcomes. The absence of evident systemic adverse effects across the investigated implant types supports the feasibility of further clinical evaluation of this coating. Moreover, the detailed evaluation of systemic safety, including liver and kidney function tests, inflammatory parameters, and serum silver levels, distinguishes this study from similar reports in the literature.

## 5. Conclusions

Given the limited sample size and absence of a control group, the findings of this study should be considered preliminary and interpreted with caution. A low pin-tract infection rate was observed in the external fixator group, whereas observations in the proximal femoral nail and partial hip arthroplasty groups primarily reflect feasibility and short-term safety rather than definitive anti-infective effectiveness. The persistence and stability of the Ag-containing calcium phosphate coating demonstrated in preclinical testing, together with the absence of systemic toxicity during clinical follow-up, supports the short-term safety of this specific electrospray-deposited coating system. Overall, these findings provide encouraging early clinical data; however, larger controlled, multicentre prospective studies with longer follow-up are required to determine its clinical anti-infective effectiveness across different orthopedic applications.

## Figures and Tables

**Table 1:** Demographic data and overall outcomes by treatment group.

Variable	ExFix (*n* = 14)	PFN (*n* = 10)	Partial Arthroplasty (*n* = 6)	Total (*n* = 30)
**Age (years), mean ± SD (IQR)**	39.71 ± 13.28 (27.50–48.25)	71.60 ± 11.41 (63.25–81.50)	68.67 ± 13.89 (60.00–78.75)	56.13 ± 19.95 (42.00–74.25)
**Sex (M/F)**	12/2	4/6	2/4	18/12
**Initial diagnosis**				
Closed fracture	0	10	4	14
Fracture-dislocation	3	0	0	3
Open fracture	6	0	0	6
Pseudarthrosis	1	0	0	1
Osteomyelitis	4	0	1	5
AVN	0	0	1	1
**Final Outcome**				
Healed	12	6	4	22
Persistent infection at final follow-up	1 patient (2 pins), persisting until pin removal	0	0	1
Pseudarthrosis	1	0	0	1
Lost to follow-up	0	1	0	1
Death	0	3	2	5

Persistent infection at final follow-up, row refers only to unresolved infection present at the time of final outcome assessment and does not represent the total number of infections occurring during follow-up. In the external-fixator group, pin-tract infection occurred in 4 of 91 pins involving 2 of 14 patients; infection resolved in one patient and persisted until pin removal in the other. In the partial hip arthroplasty group, two early postoperative PJIs occurred, both of which resolved after implant-retaining treatment and were therefore classified as healed at final follow-up.

**Table 2:** Clinical diagnoses, number of patients, number of pins, and cases with discharge during follow-up in the external fixator group.

Clinical Diagnosis	Number of Patients	Number of Pins	Discharge During Follow-Up (*n*)
Chronic osteomyelitis	4	29	2 (6.9%)
Nonunion (pseudarthrosis)	2	12	0
Closed fracture + dislocation	2	11	0
Open fracture + dislocation	3	20	0
Open fracture	3	19	2 (10.5%)
Total	14	91	4 (4.4%)

## Data Availability

The original contributions presented in this study are included in the article/Supplementary Materials. Further inquiries can be directed to the corresponding author.

## Supplementary Materials

The following supporting information can be downloaded at: https://www.mdpi.com/article/10.3390/jfb17100517/s1, Supplementary File S1: Engineering thesis; Supplementary File S2: Medical specialty thesis; detailed clinical laboratory data, microbiological culture results, and additional methodological and characterization data related to the preparation, coating, and evaluation of the silver ion-doped calcium phosphate coating.

## Abbreviations

The following abbreviations are used in this manuscript:

CRP	C-reactive protein
ESR	Erythrocyte sedimentation rate
PJI	Periprosthetic joint infection
DAIR	Debridement, antibiotics, and implant retention
HA	Hydroxyapatite
CaP	Calcium phosphate
Ag	Silver
ICP-MS	Inductively coupled plasma mass spectrometry
