# Supplementary material for: Safety and Preliminary Clinical Outcomes of Silver Ion-Doped Calcium Phosphate-Coated Orthopedic Implants: A Prospective Pilot Study
Source: J Funct Biomater. 2026 Oct 15;17(10):517. doi: 10.3390/jfb17100517 (PMC13706277; doi:10.3390/jfb17100517)
Supplement: Supplementary file 1 [file jfb-17-00517-s001.zip › supplementary file S1 engineering thesis.pdf]

SUPPLEMENTARY FILE S1

Materials-Engineering Characterization of the Silver Ion–Doped Calcium Phosphate Coating

1. Introduction

The purpose of this supplementary file is to provide a comprehensive and detailed description of the experimental procedures related to the preparation, coating, and evaluation of silver-ion-doped calcium phosphate ceramics on metallic implants. While the main article summarizes the overall methodology and presents the key results, the whole technical workflow requires a more extensive explanation than can be accommodated within the main text.

This document presents the study in detail, covering all stages, including powder synthesis, aerosol and electrospray deposition methods, post-deposition heat treatment, microstructural and chemical analyses, in vitro dissolution and stability testing, and post-clinical characterization of retrieved implants. Figures and tables are cited with their original numbering from the dissertation to ensure clarity and consistency, allowing direct cross-reference to the experimental data.

This supplementary file has been prepared to enhance transparency, ensure reproducibility of the described methods, and provide readers with access to the complete technical background that supports the findings reported in the article.

2. Powder Preparation and Characterization

Silver-ion-doped calcium phosphate powders were synthesized using the wet chemical precipitation method. Calcium nitrate tetrahydrate [$Ca(NO_3)_2 \cdot 4H_2O$] and diammonium hydrogen phosphate [$(NH_4)_2HPO_4$] served as the primary precursors, while silver nitrate ($AgNO_3$) was introduced as the silver source. Precursor solutions were prepared in deionized water, and the pH was carefully adjusted to either 5.5 or 8.5 using ammonium hydroxide. The precipitation was carried out under magnetic stirring, followed by filtration, drying at 100 °C, and subsequent calcination at 1000 °C for 2 hours. This procedure yielded powders with different phase compositions depending on the synthesis pH and thermal treatment.

Phase characterization was conducted using X-ray diffraction (Rigaku Rint and Bruker D8 Advance Twin, Cu Kα radiation, 40 kV, 30 mA, scan range 20°–80°). The results confirmed the formation of hydroxyapatite and tricalcium phosphate phases, with hydroxyapatite being dominant at pH 5.5 and a greater proportion of TCP forming at pH 8.5. Silver incorporation was verified by characteristic diffraction peak shifts, supporting its integration into the calcium phosphate lattice (Figure 1–4, Table 1).

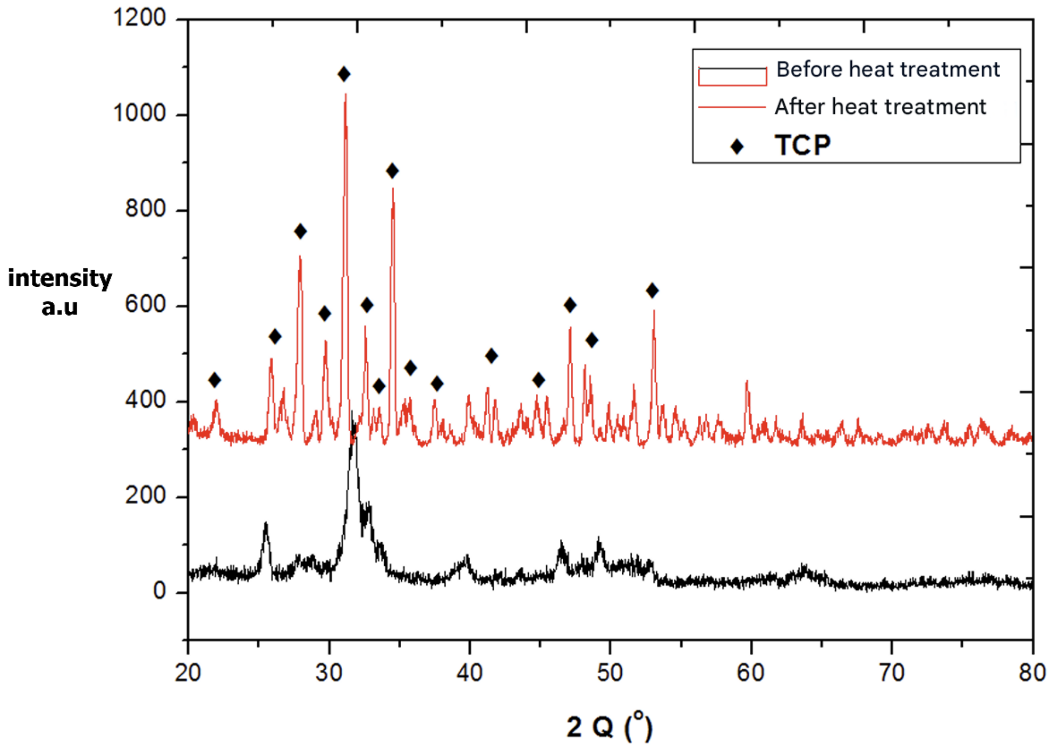

FİGURE 1: XRD lattice pattern of pH 5.5

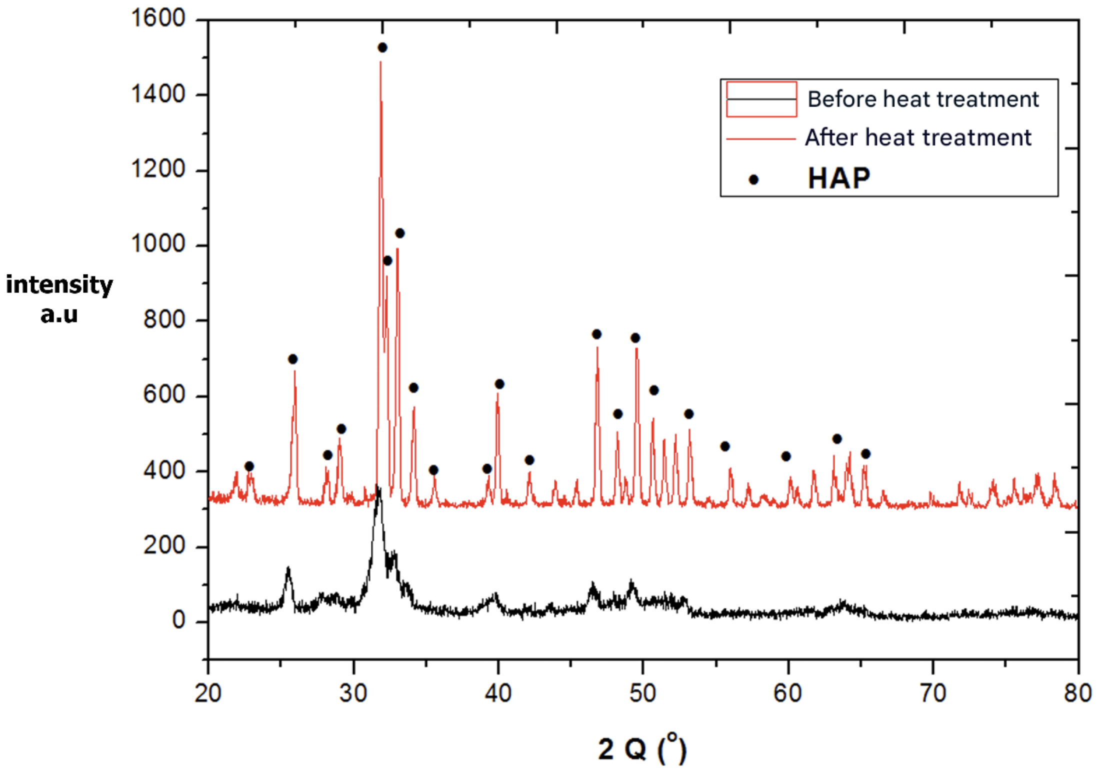

FIGURE 2: XRD lattice pattern of pH 8.5

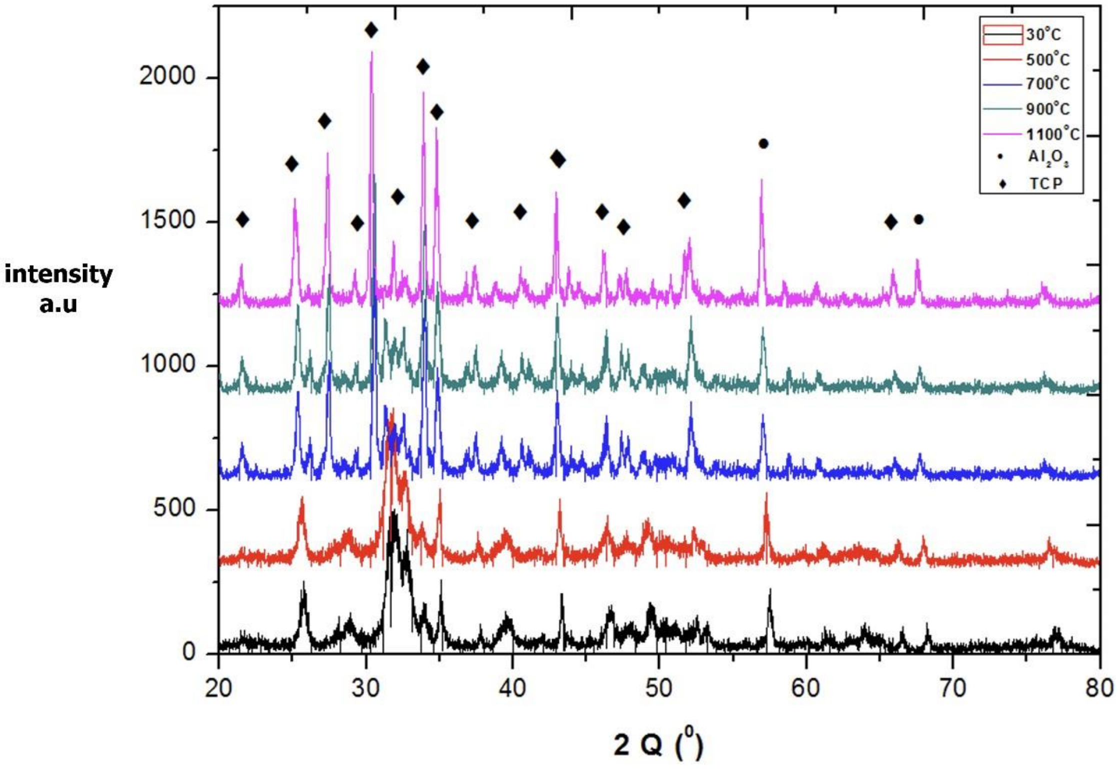

FIGURE 3 : Temperature-dependent XRD lattice patterns of the final pH value of 5.5 and uncalcined powder

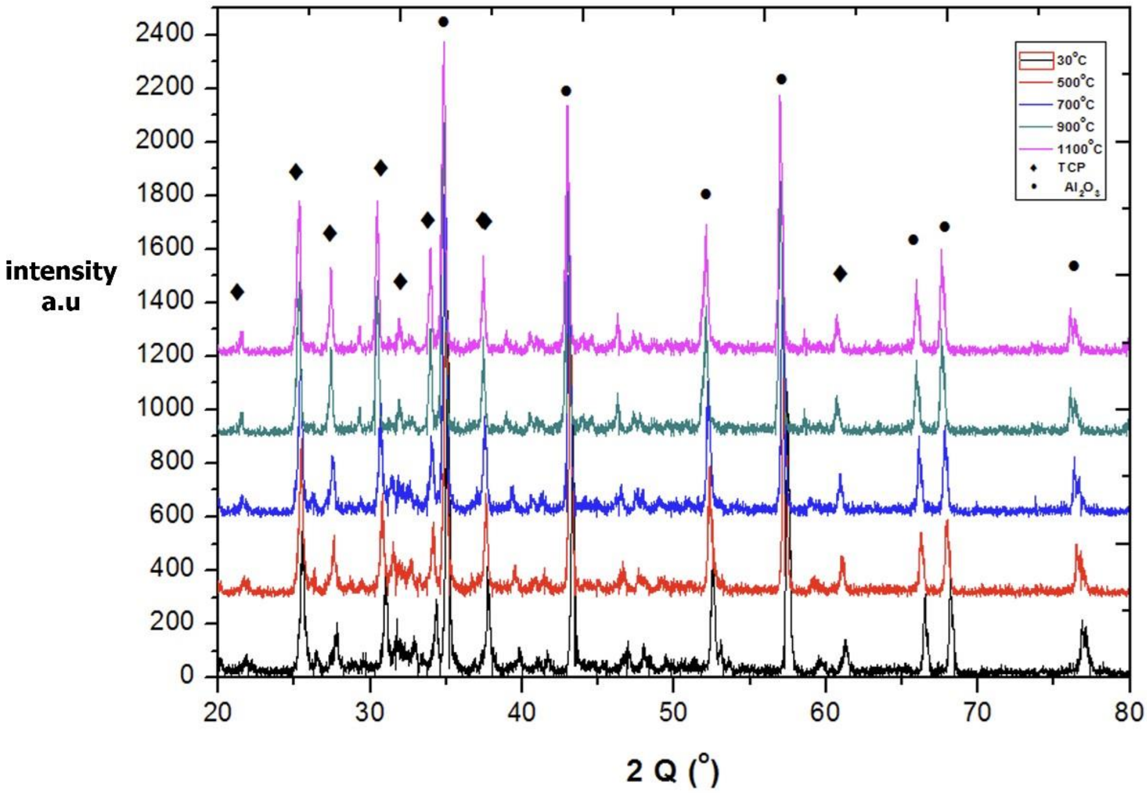

FIGURE 4: Temperature-dependent XRD lattice patterns of the final pH value of 8.5 and uncalcined powder.

Powder ID	$AgNO_3$ added (g)	Ag added (g)	Ag added (%)	EDX result (Ag) 1st measurement	EDX result (Ag) 2nd measurement
POWDER-0	1.10	0.698	11.27	13.38	10.37
POWDER-1	0.45	0.571	9.41	9.12	8.41
POWDER-2	0.375	0.428	7.23	7.50	7.39
POWDER-3	0.225	0.285	4.94	4.10	4.49
POWDER-4	0.11	0.142	2.53	1.31	2.30
POWDER-5	0.05	0.063	1.14	1.00	1.23

TABLE 1: Chemical analysis results of the synthesized antibacterial powders.

Scanning electron microscopy (Zeiss EVO LS10) provided detailed microstructural information before and after calcination. Powders synthesized at pH 5.5 in their as-prepared state displayed nanoscale spherical morphologies averaging 80–120 nm. After calcination at 1000 °C, the particles grew slightly in size but retained their agglomerated spherical morphology. In contrast, powders prepared at pH 8.5 exhibited larger grain sizes of approximately 200–300 nm following heat treatment, indicating that both the synthesis pH and calcination significantly influenced grain growth and microstructural evolution (Figure 5)

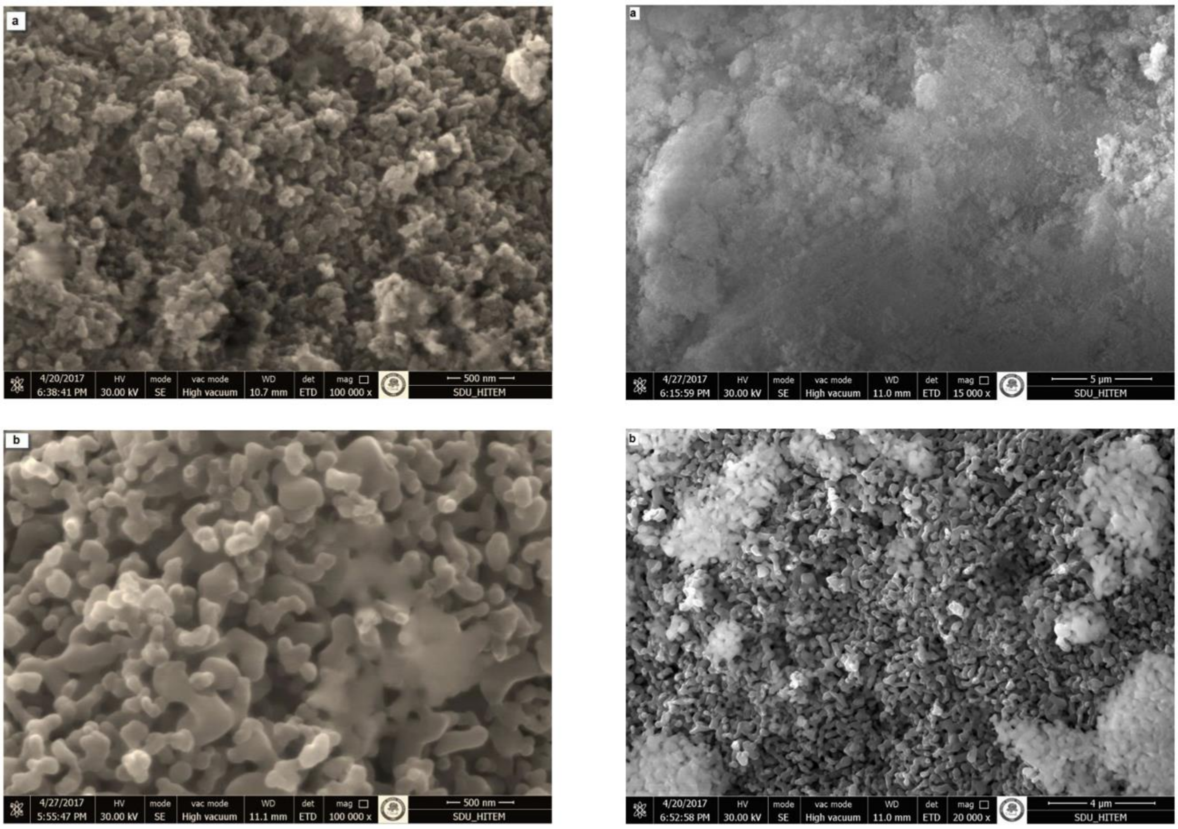

FIGURE 5: On the left is the microstructure of the powder with a final pH of 5.5. a) Uncalcined b) Calcined at 1000°C. On the right is the microstructure of the powder with a final pH of 8.5. a) Uncalcined b) Calcined at 1000°C.

Energy-dispersive X-ray spectroscopy further confirmed the successful incorporation of silver. The spectra demonstrated consistent detection of calcium, phosphorus, oxygen, and silver across all samples, verifying the chemical composition of the synthesized powders and supporting the uniform distribution of silver ions (Figure 6).

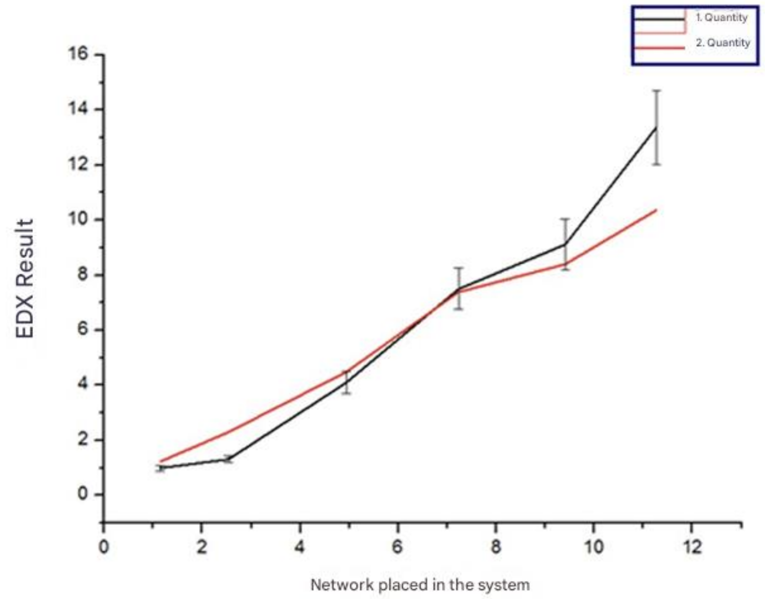

FIGURE 6: Comparison of the initial Ag amount with the Ag amounts read after characterization.

Thermal behavior of the materials was examined using hot-stage microscopy (Figure 7). This analysis revealed that structural changes began to occur between 400 and 800 °C, corresponding to the decomposition of precursor phases and the onset of densification. At approximately 1000 °C, the powders reached a stable crystalline state dominated by hydroxyapatite and tricalcium phosphate. These results aligned with the XRD findings, confirming that calcination at 1000 °C was sufficient to achieve stable and crystalline CaP–Ag powders suitable for subsequent coating applications.

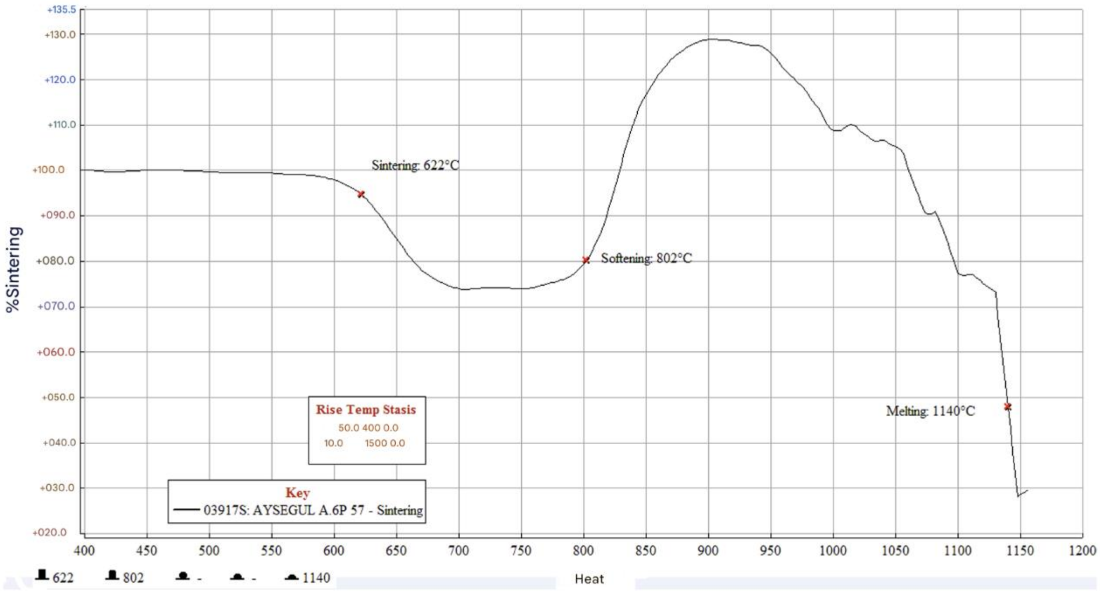

FIGURE 7: Heat microscope analysis result of 6P57 bioglass.

In summary, wet chemical synthesis enabled controlled incorporation of silver into calcium phosphate powders. XRD confirmed the expected crystalline phases, SEM highlighted nanoscale morphologies with particle size growth after calcination, EDX validated chemical composition, and hot-stage microscopy demonstrated thermal stability at elevated temperatures. Together, these findings established a reliable basis for employing the synthesized powders in implant coating procedures.

3. Aerosol Deposition Coating of Metal Implants

Aerosol deposition (AD) was used to coat metallic implant surfaces with silver-ion-doped calcium phosphate powders synthesized as described in Section 1. The process was performed in a custom-built chamber containing a powder feeder, a carrier gas system, a nozzle, and a substrate holder. Before coating, stainless steel and titanium substrates, as well as external fixators and intramedullary nails, were ultrasonically cleaned in ethanol and dried with nitrogen (Figure 8,9).

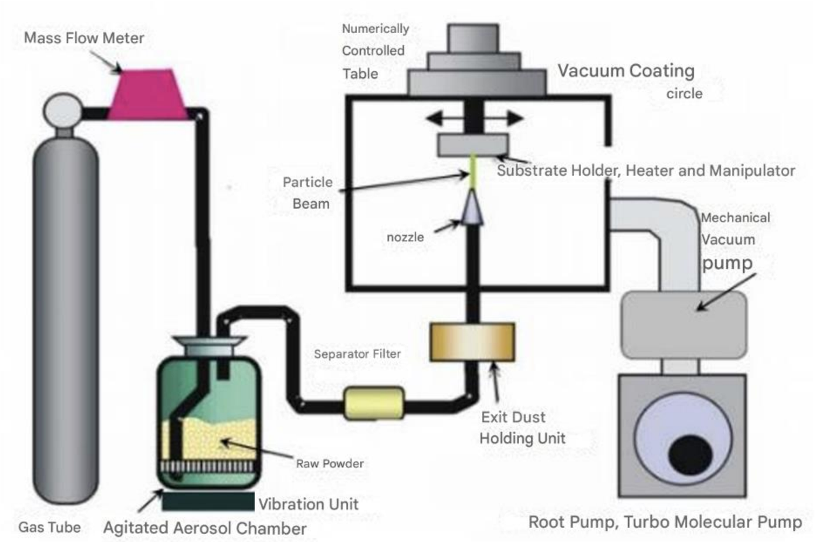

FIGURE 8: Schematic view of the aerosol deposition method.

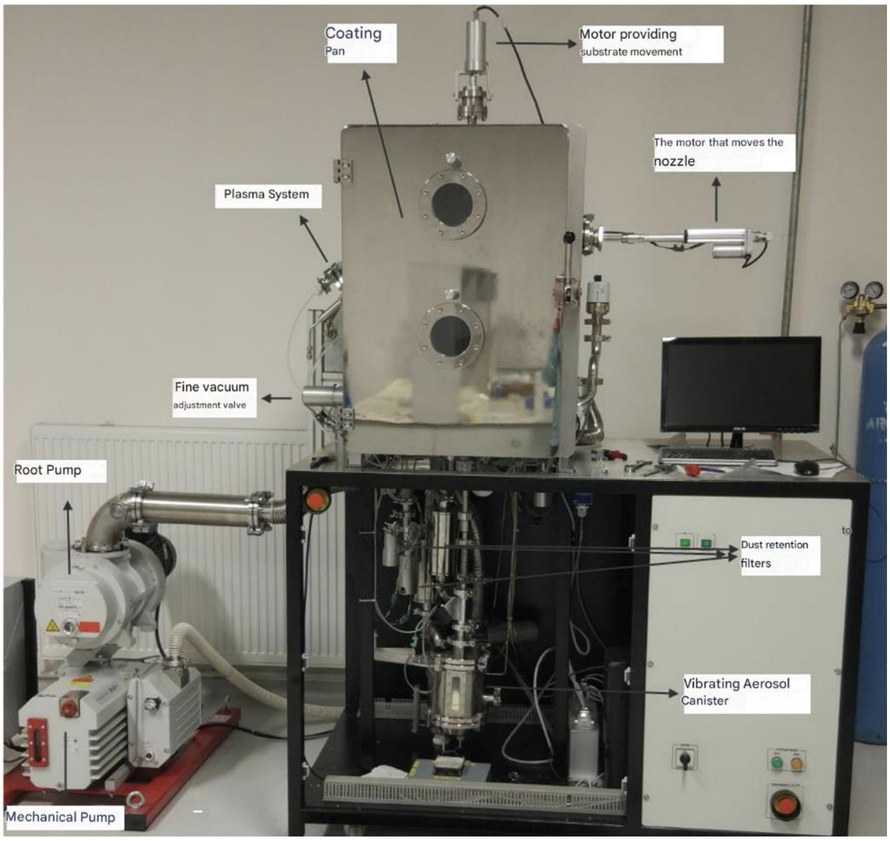

FIGURE 9: Designed Aerosol Deposition System.

Different particle sizes were tested to evaluate their effect on coating morphology, as indicated in the optical microscopy images (Figure 10). Smaller particles (0.06–0.2 µm) formed dense and homogeneous coatings. In contrast, larger powders (~0.8 µm) led to rougher, less adherent surfaces.

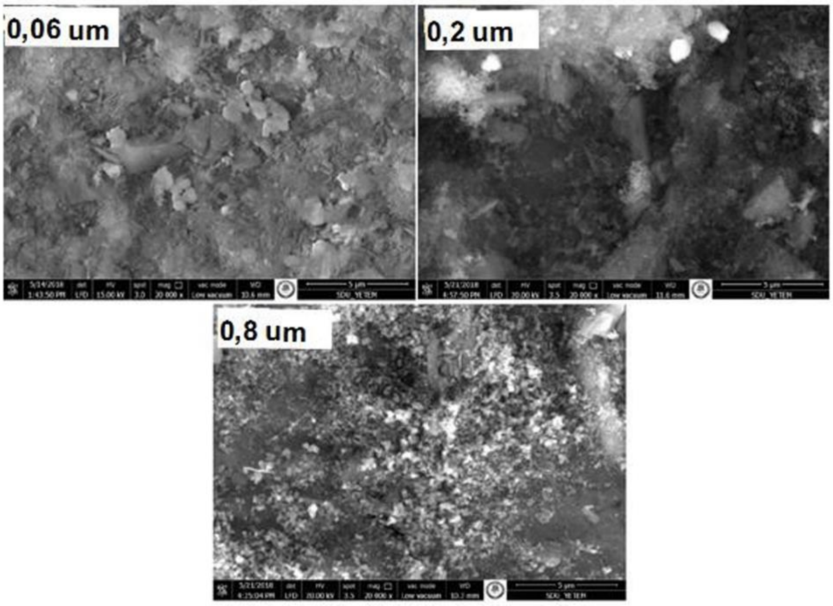

FIGURE 10: Microstructure images of coatings made with powders having grain sizes of 0.06, 0.2 and 0.8 µm.

Gas flow rate was another key parameter: at 1000 cm³/min, coatings were thin and discontinuous, while at 5000–10,000 cm³/min, the coatings were dense and uniform. Excessive flow of 15,000 cm³/min produced local damage and microcracks due to high particle impact (Figure 11- 17; Tablo 3.3).

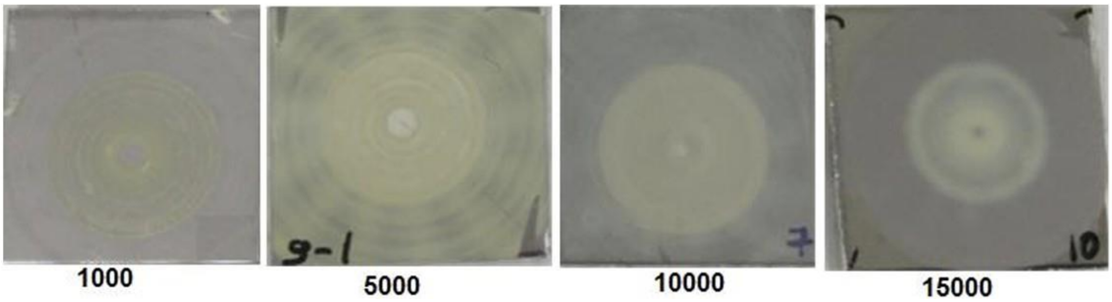

FIGURE 11: Images of substrates coated at different gas flow rates.

Parameter	Value
**Gas Flow Rate**	1,000 cm³/min ; 5,000 cm³/min ; 10,000 cm³/min; 15,000 cm³/min
**Substrate-Nozzle Distance**	• 5 mm
**Number of Passes**	• 10 passes
**Nozzle Traverse Speed**	• 2 mm/s
**Substrate Rotation Speed**	• 30 rpm
**Powder Particle Size**	• 0.06 µm

TABLE 2: Coating parameters used at different gas flow rates

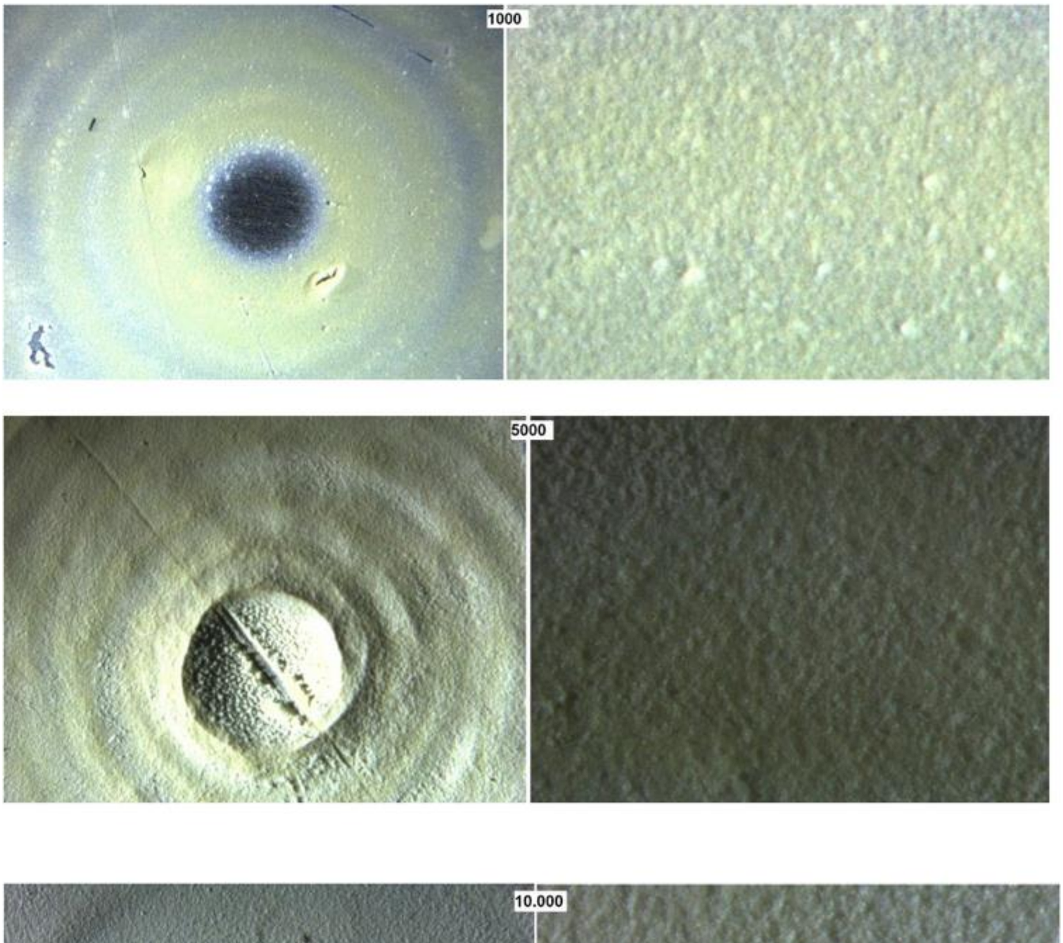

FIGURE 12:Optical images of coatings applied at different gas flow rates (1,000, 5,000, 10,000, 15,000 cm3/min).

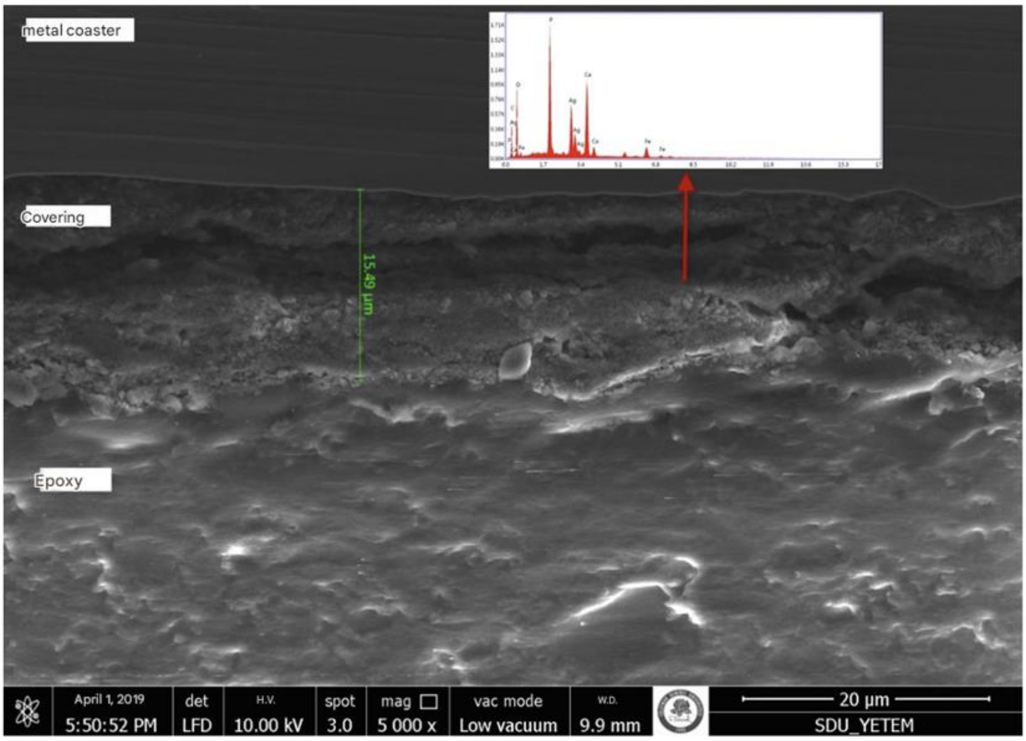

(a)

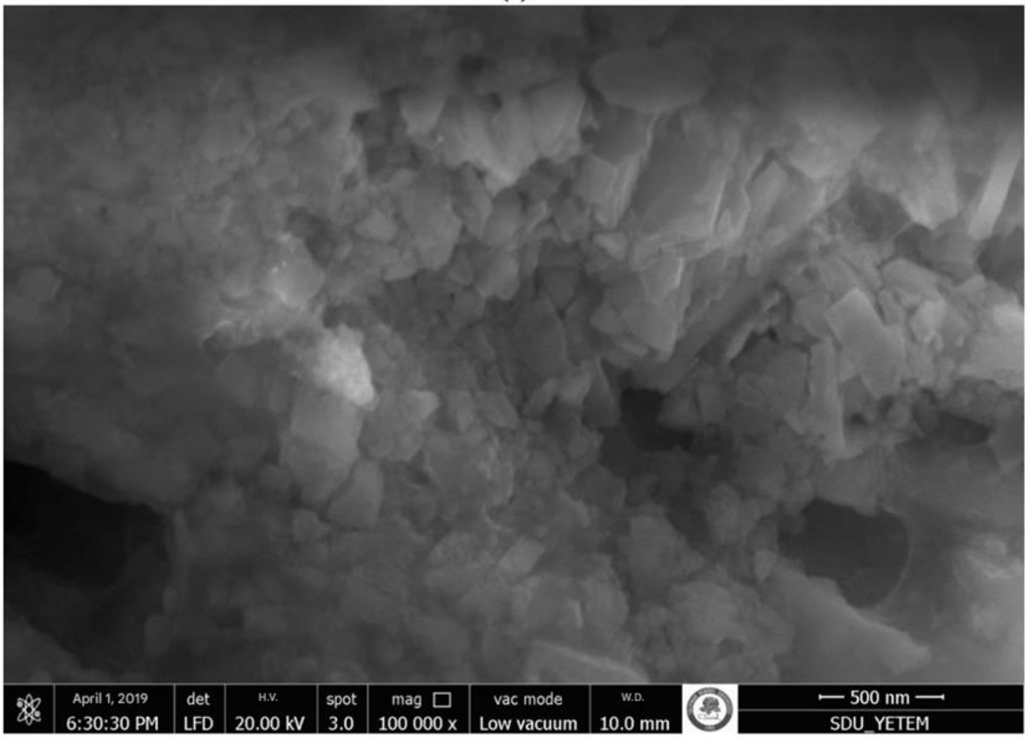

FIGURE 13: a) Cross-section b) Surface SEM image of the coating with a gas flow of 1,000 cm3/min.

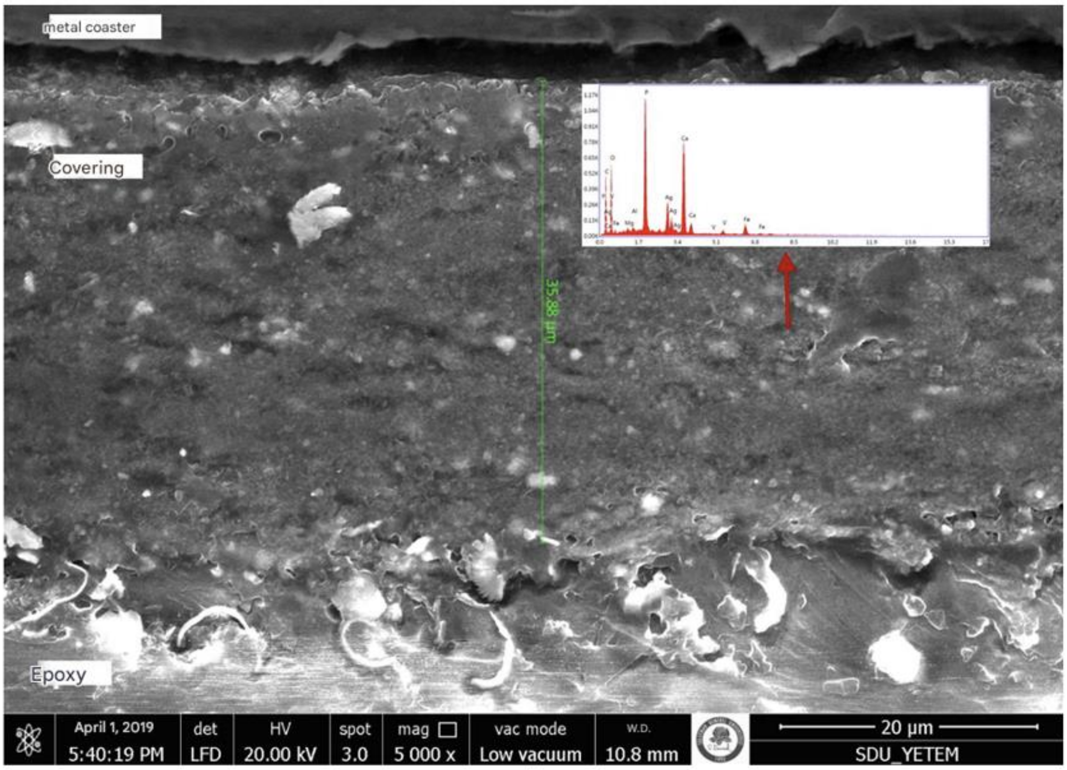

(a)

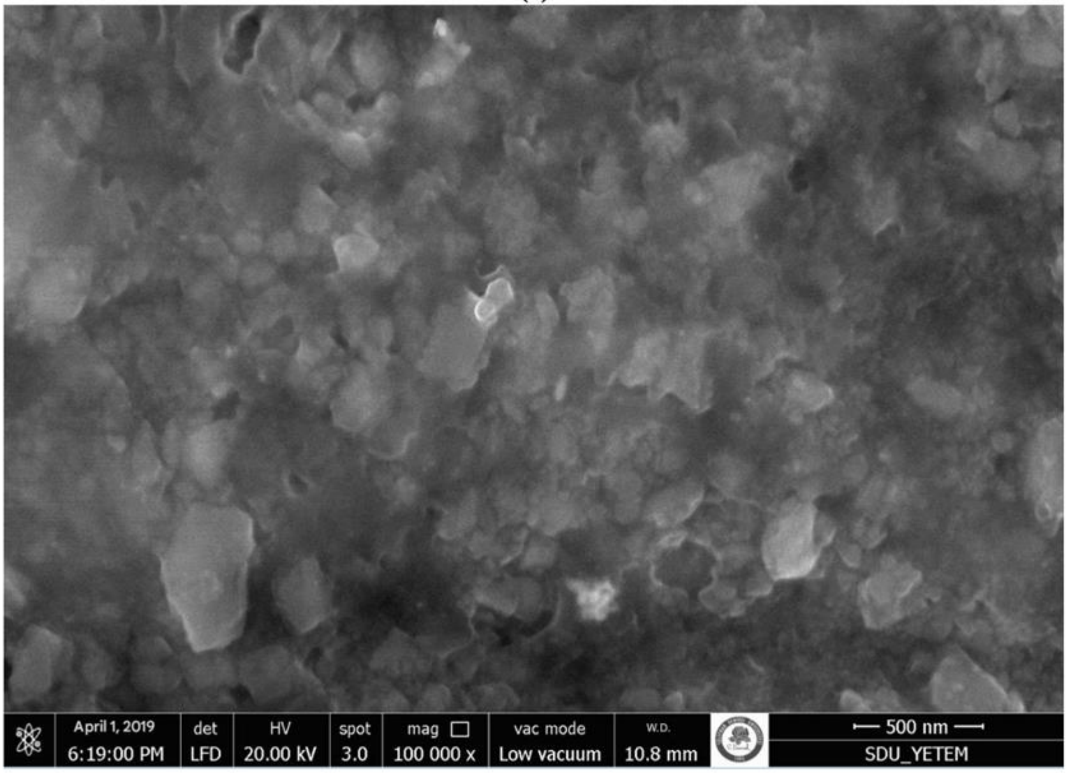

(b)

FIGURE 14: a) Cross-section b) Surface SEM image of the coating with a gas flow of 5,000 cm3/min.

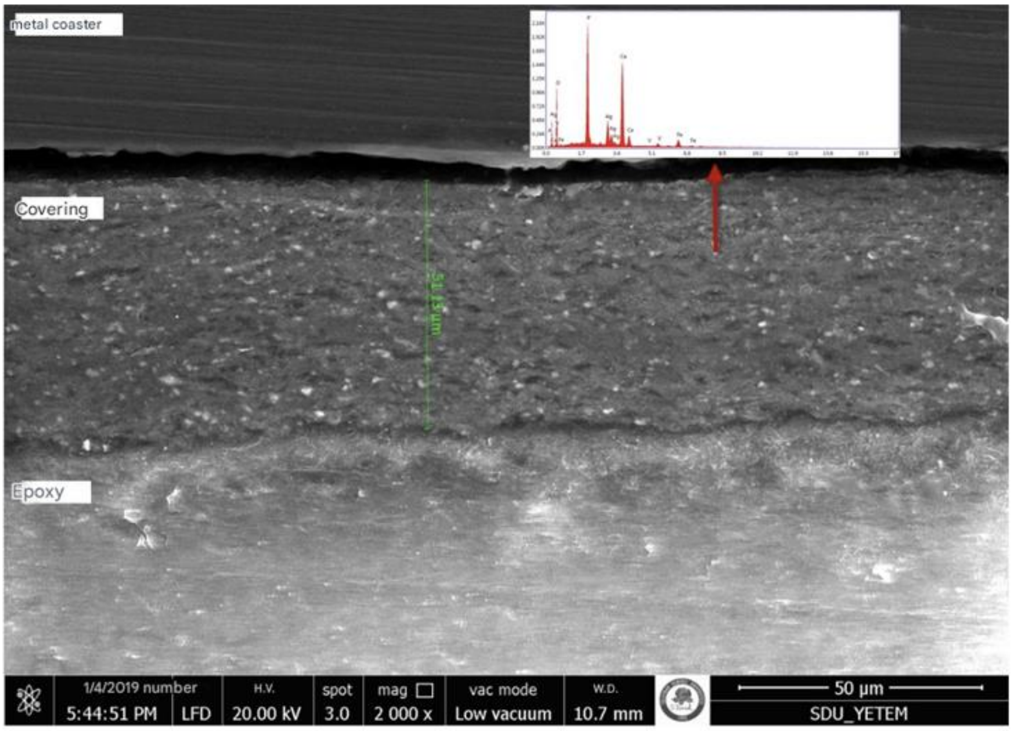

(a)

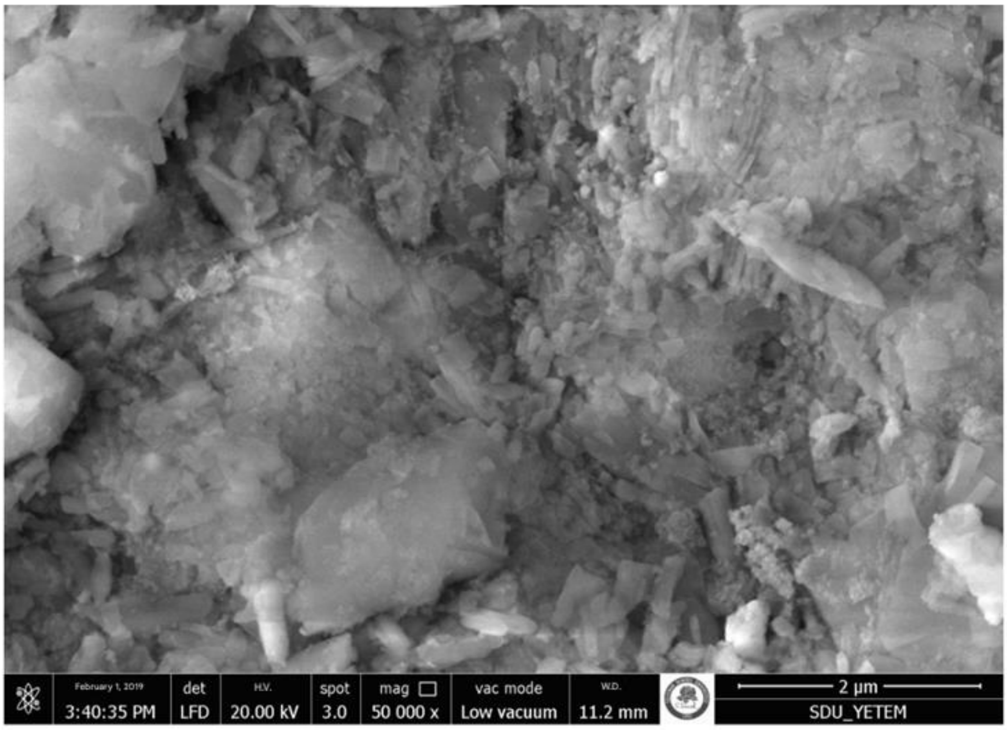

(b)

FIGURE 15: a) Cross-section b) Surface SEM image of the coating with a gas flow of 10,000 cm3/min.

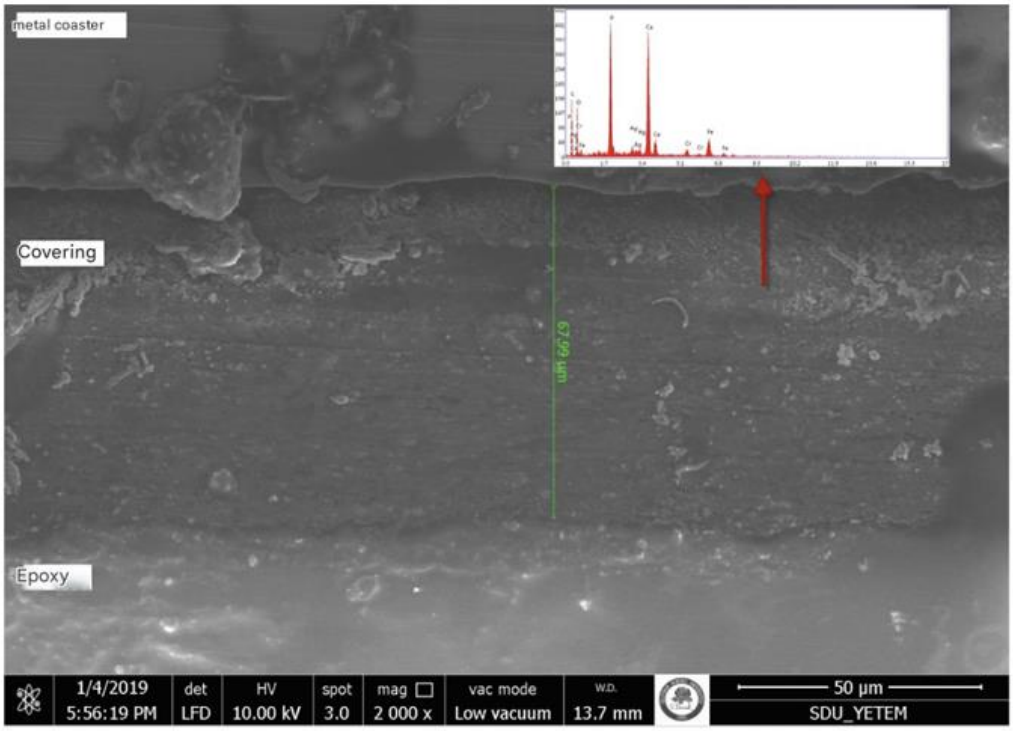

(a)

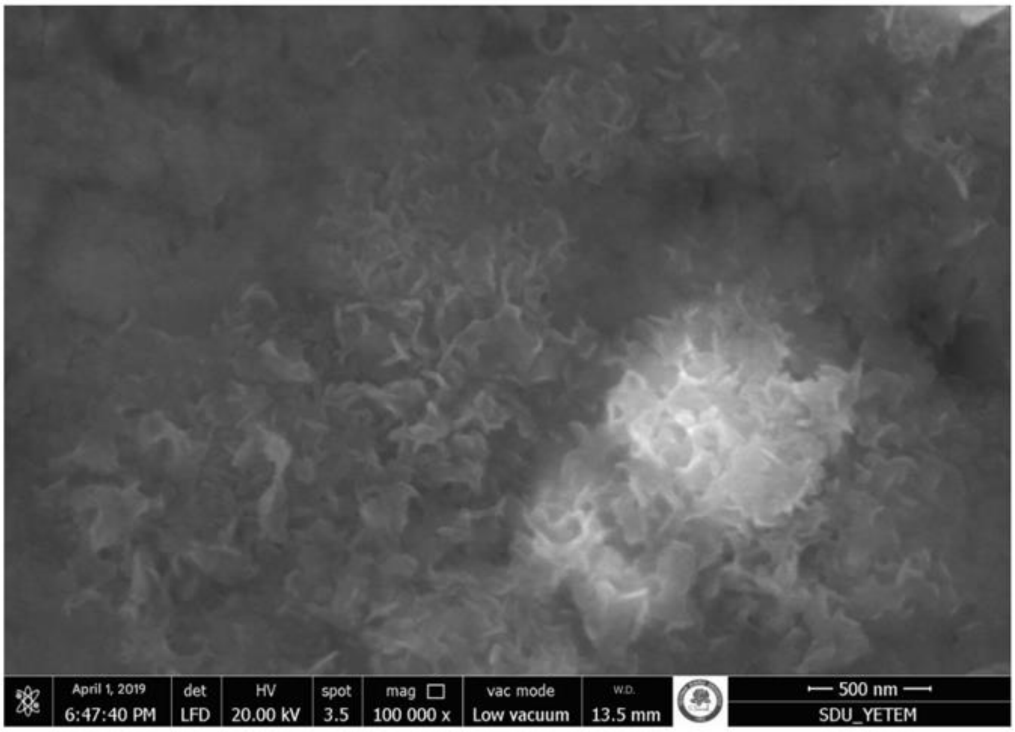

(b)

FIGURE 16: a) Cross-section b) Surface SEM image of the coating with a gas flow of 15,000 cm3/min.

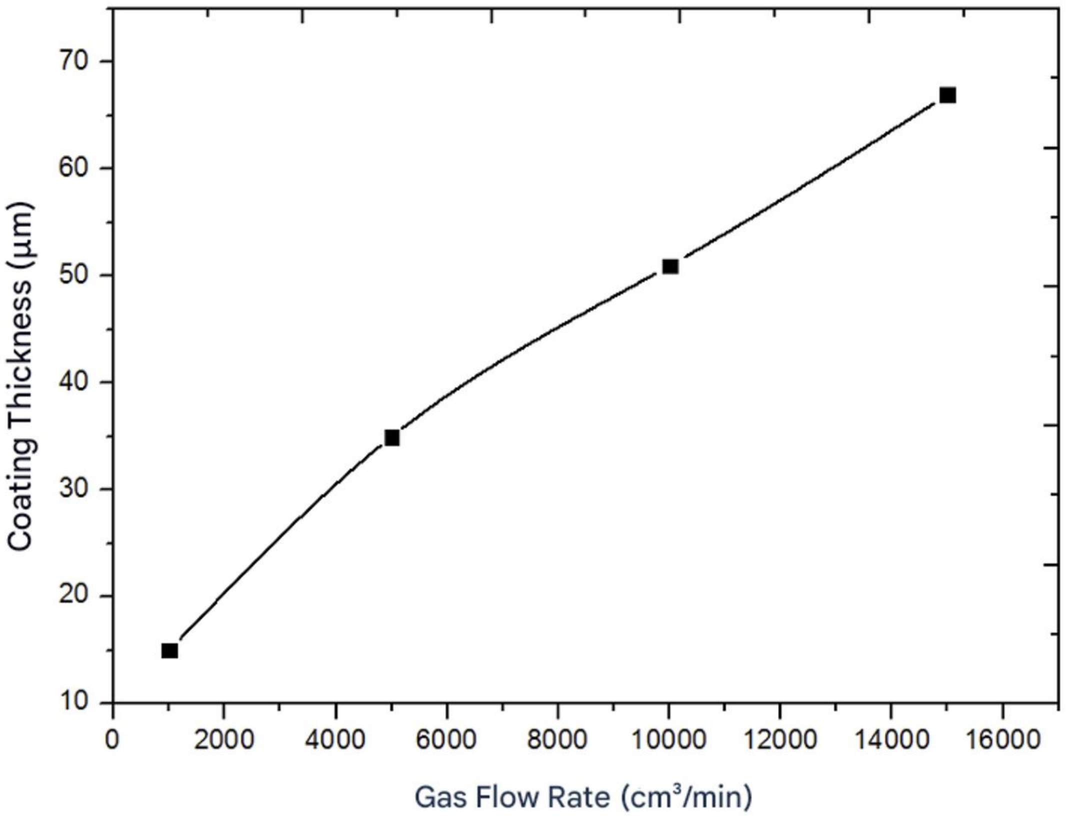

FIGURE 17: Effect of gas flow rate on coating thickness

The nozzle–substrate distance strongly influenced coating thickness and adhesion. SEM and cross-sectional images demonstrated that a 5 mm distance resulted in the thickest coatings (~12–15 µm), 10 mm provided a balanced thickness with good adhesion, and 15 mm led to thinner layers with partial delamination (Figure 18-23 ; Tablo 3).

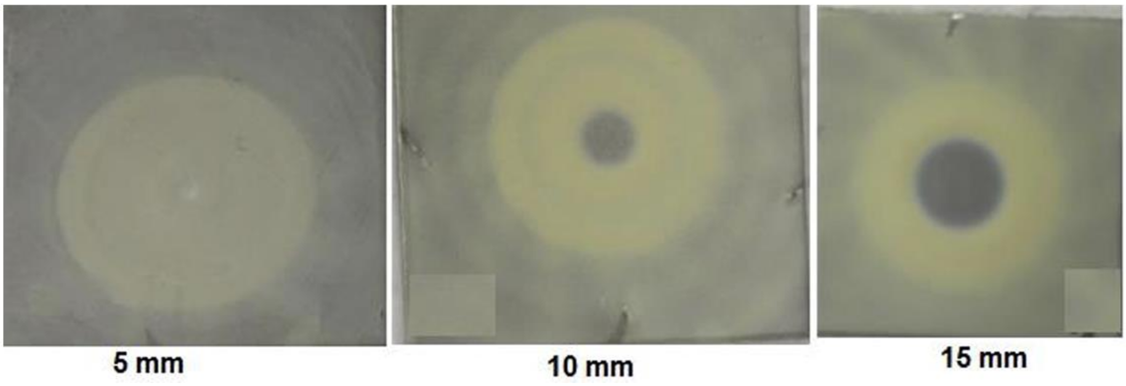

FIGURE 18: Images of substrates covered at different distances.

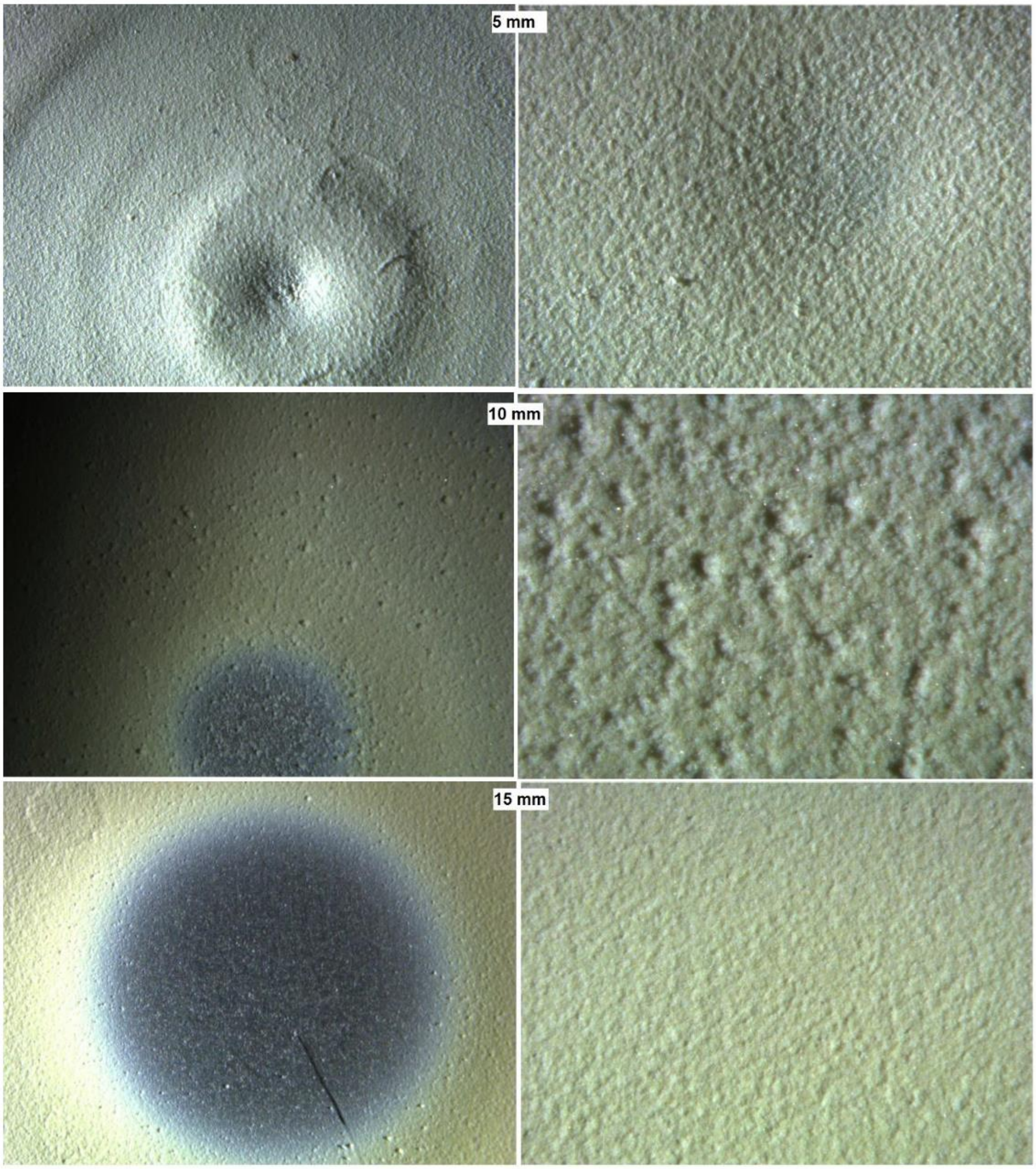

FIGURE 19: Optical images of coatings applied at different nozzle-to-substrate distances (5 mm, 10 mm, 15 mm).

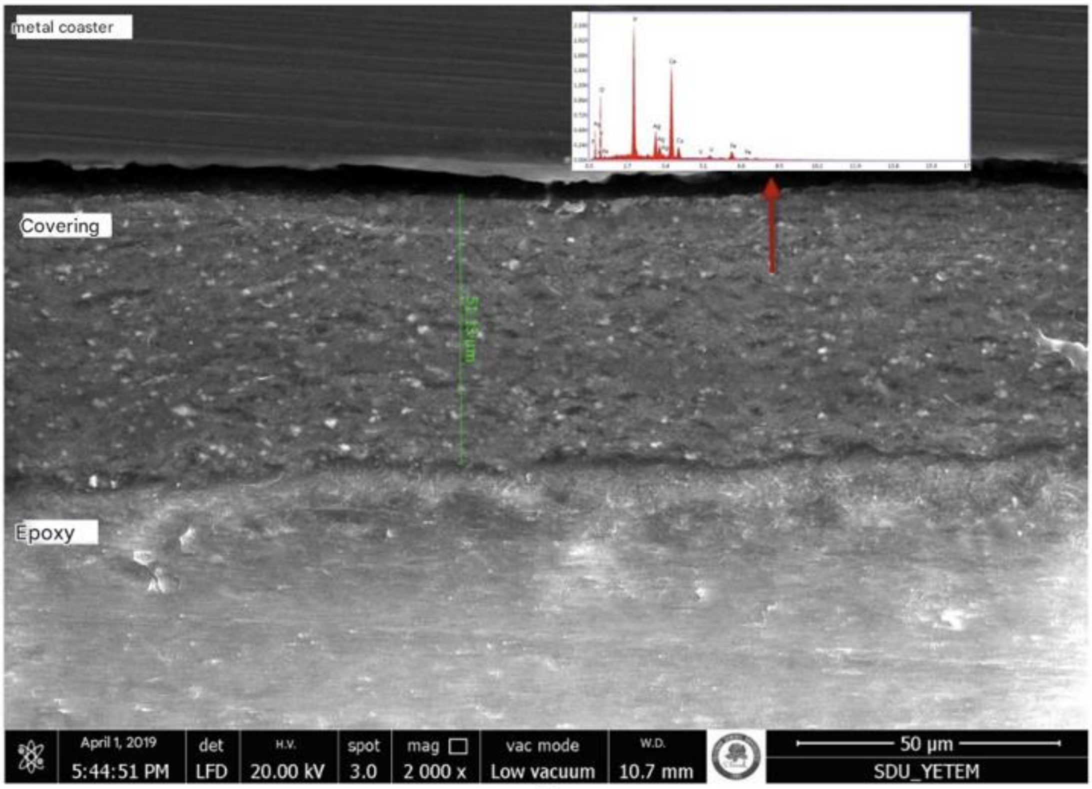

(a)

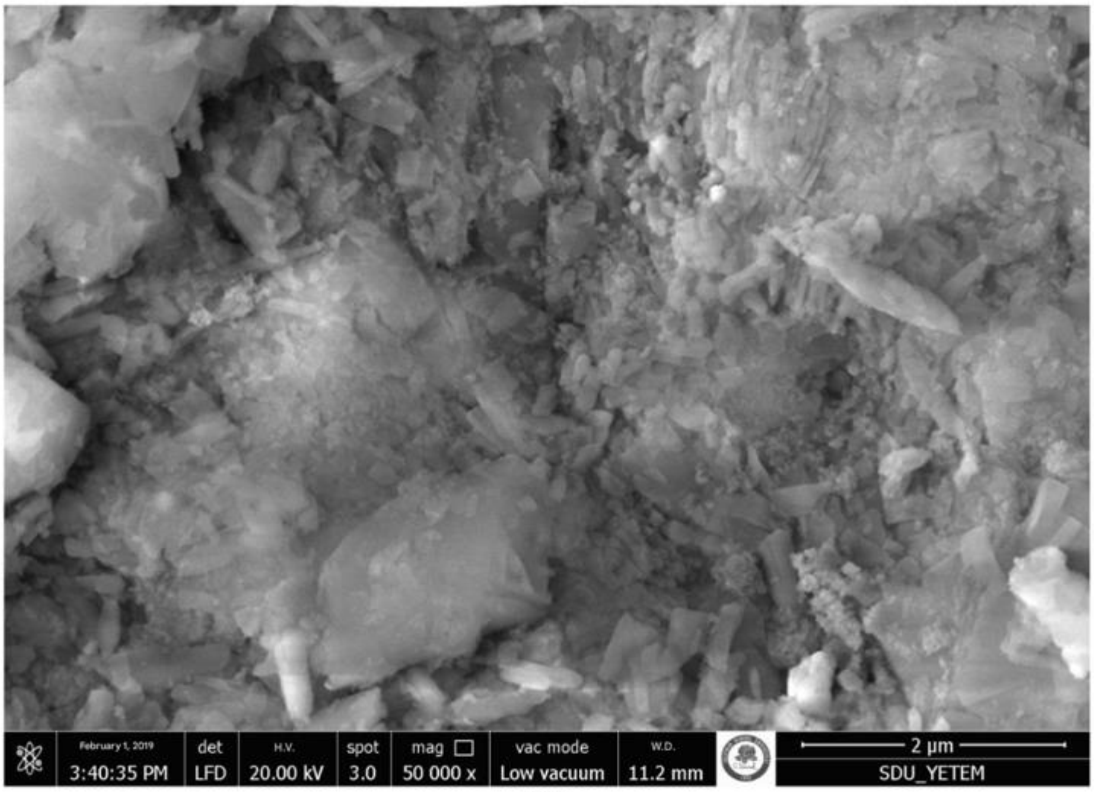

(b)

FIGURE 20: a) Cross-section b) Surface view of the coating made with a distance of 5 mm.

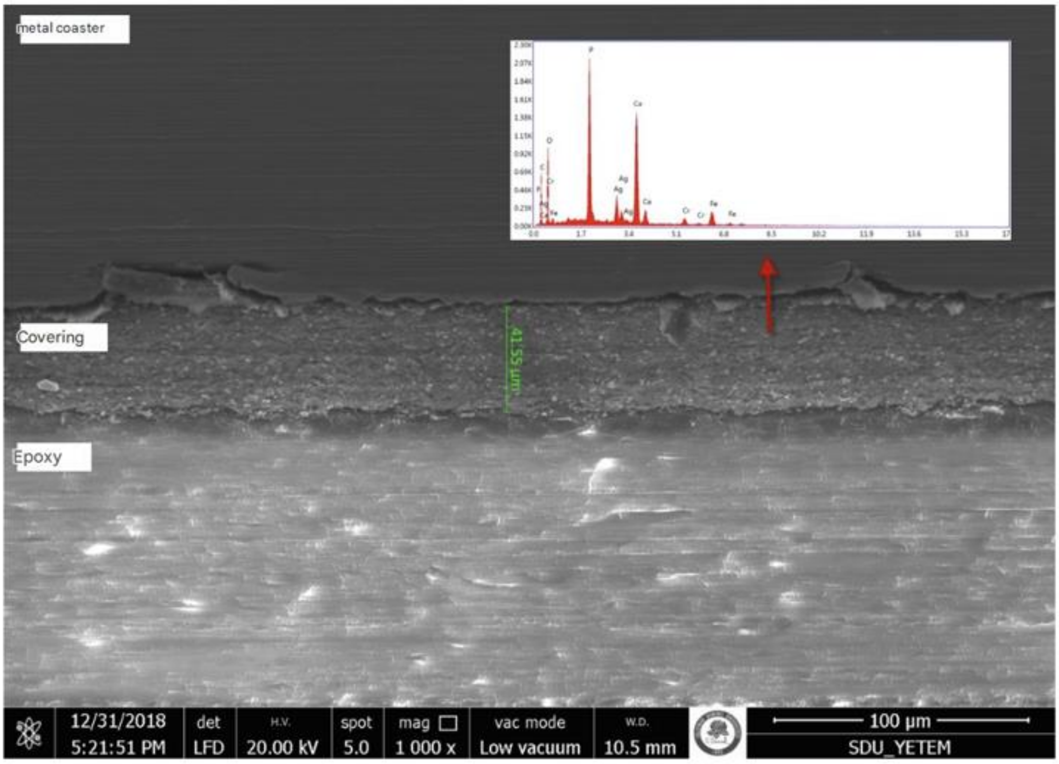

(a)

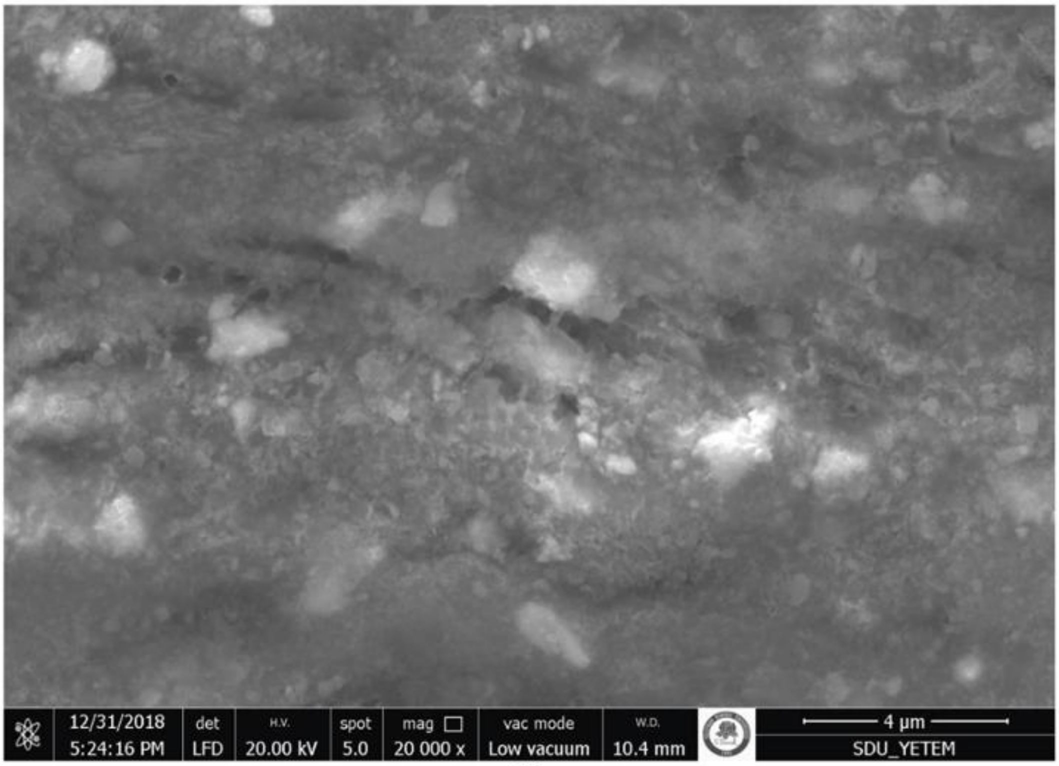

(b)

FIGURE 21: a) Cross-section b) Surface view of the coating made with a distance of 10 mm.

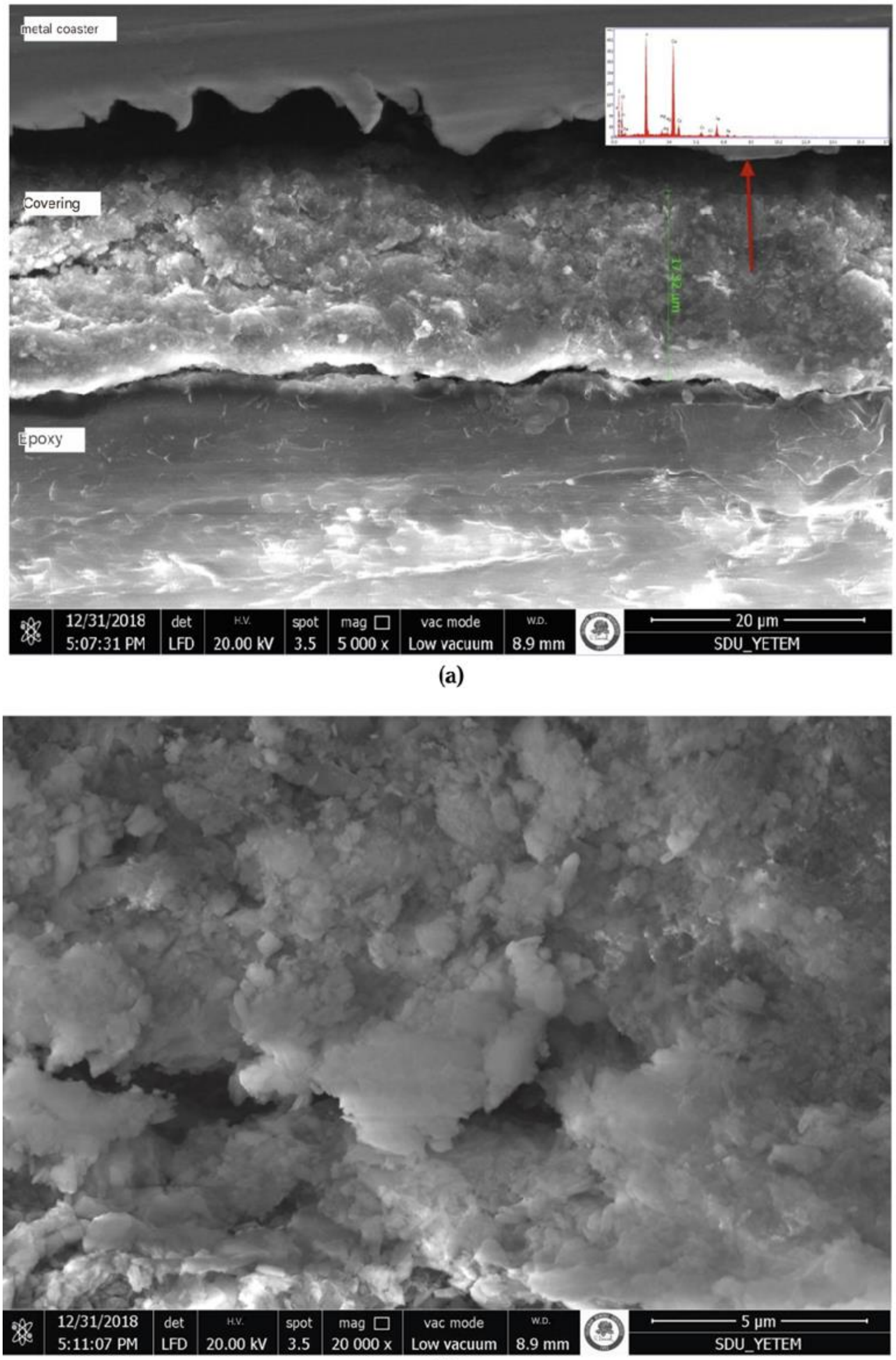

(b)

FIGURE 22: a) Cross-section b) Surface view of the coating made with a distance of 15 mm.

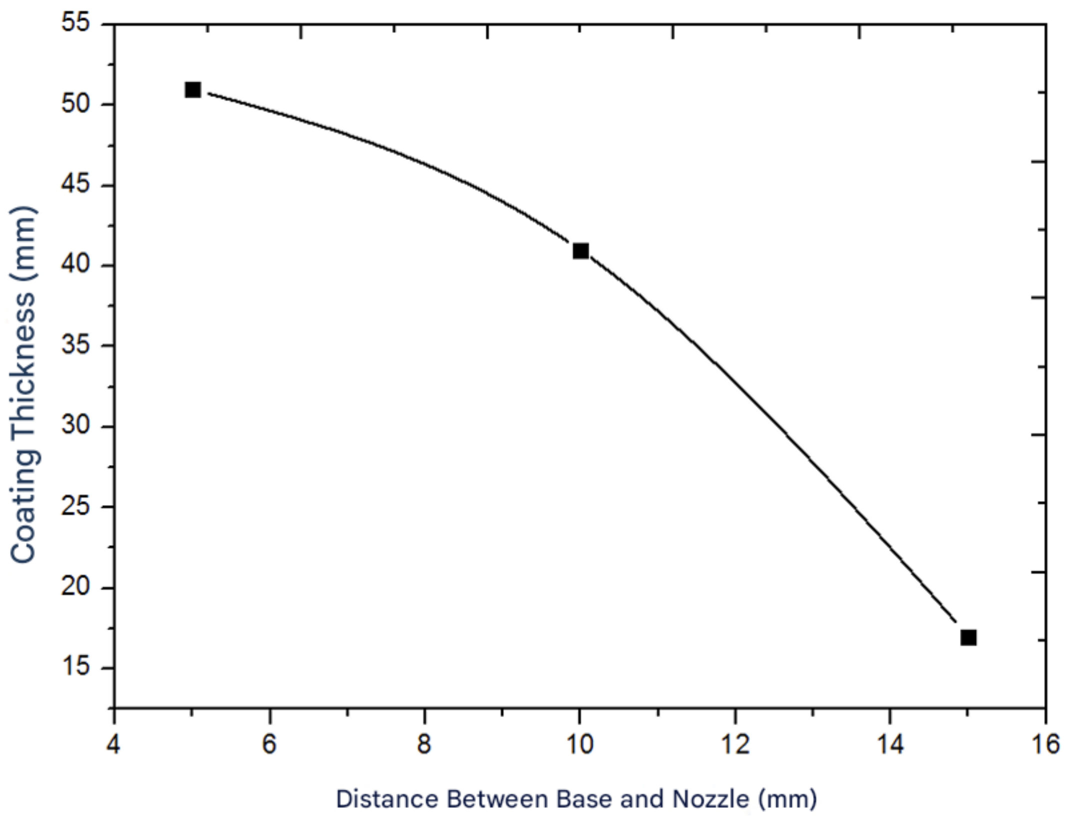

FIGURE 23: Effect of distance between substrate and nozzle on coating thickness.

**Parameter**	**Value(s)**
Gas flow rate	10,000 cm^3/min
Nozzle–substrate distance	5 mm, 10 mm, 15 mm
Number of passes	10
Nozzle traverse speed	2 mm/s
Substrate rotation speed	30 rpm
Powder particle size	0.06 µm

TABLE 3: Coating parameters at different distances.

Plasma assistance was used to enhance adhesion strength further. As observed in the SEM and optical images, 50 W plasma yielded partial bonding, 100 W provided optimal adhesion and coating uniformity, whereas 150 W induced microcracking due to overheating (Figure 24,25 ; Tablo 4).

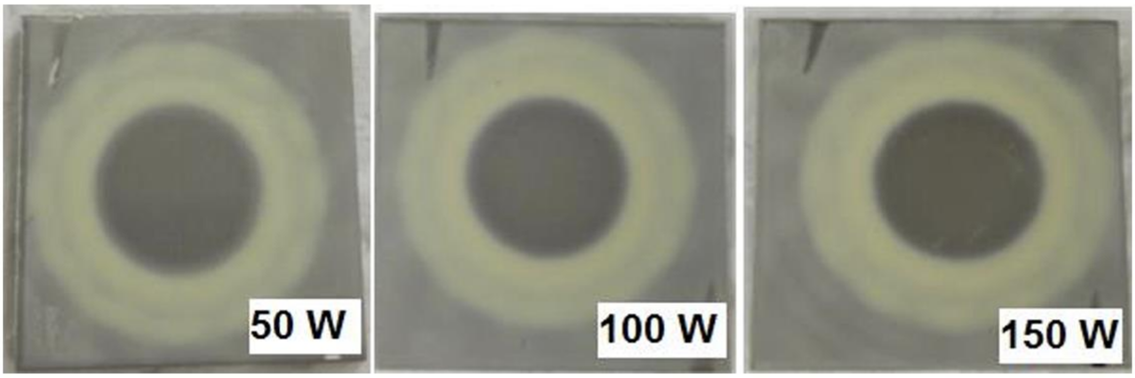

FIGURE 24: Image of coatings with different RF induction voltages applied.

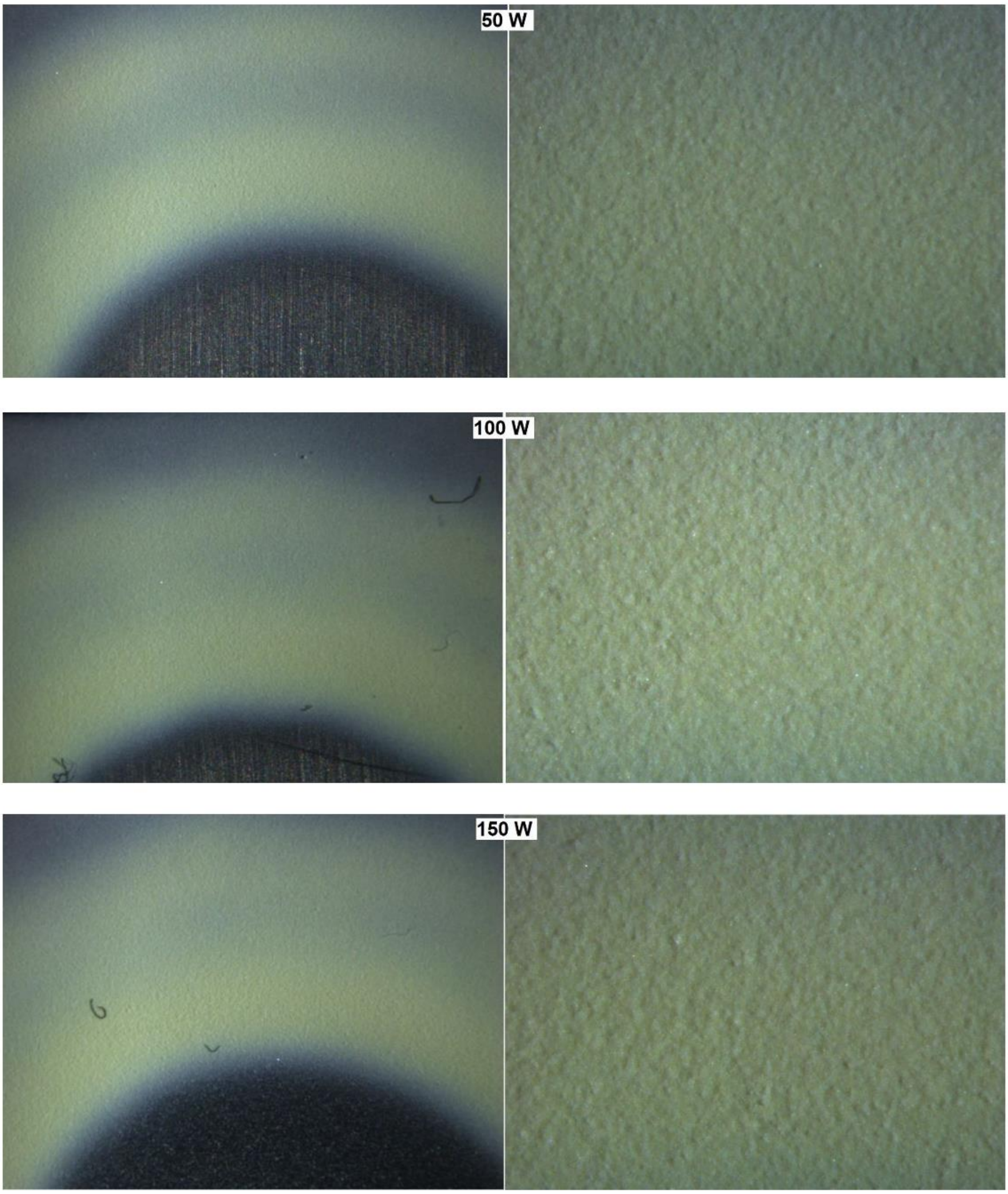

FIGURE 25: Optical images of coatings exposed to plasma at 50W, 100W and 150W.

Parameter	Value(s)
Gas flow rate	10,000 cm³/min
Nozzle–substrate distance	15 mm
Number of passes	10
Nozzle traverse speed	2 mm/s
Substrate rotation speed	30 rpm
Powder particle size	0.06 µm
RF induction	50 V – 5 min100 V – 5 min150 V – 5 min

TABLE 4: Coating parameters used to evaluate the effect of RF induction.

Surface roughness was quantified with the TESKOM TR200 profilometer, which is shown in Figure 26.

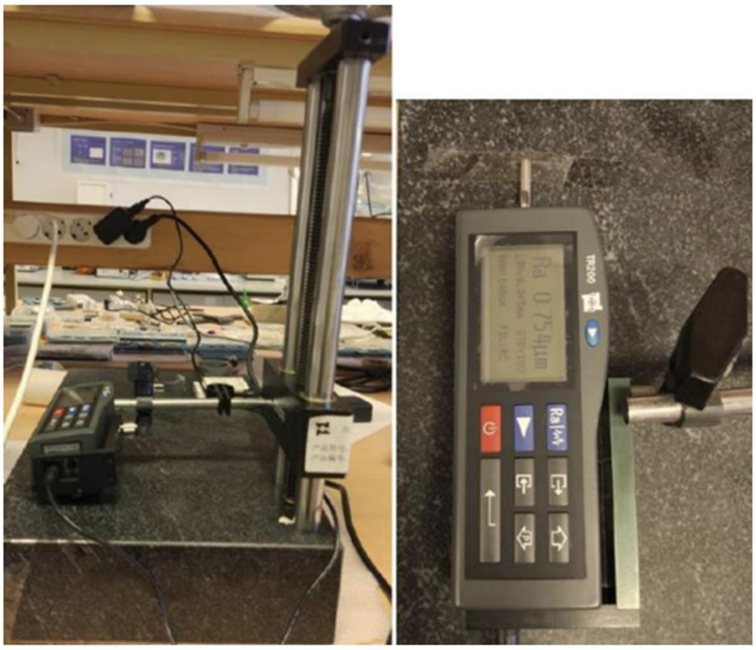

FIGURE 26: TESKOM TR200 surface roughness measuring device.

Measurements revealed Ra values between 0.25 and 1.2 µm, with the smoothest surfaces achieved under optimized flow (5000–10,000 cm³/min) and 10 mm nozzle distance (Tablo 3.6). Adhesion properties were tested using scratch testing, as illustrated by the schematic of the test setup (Figure 27-29).

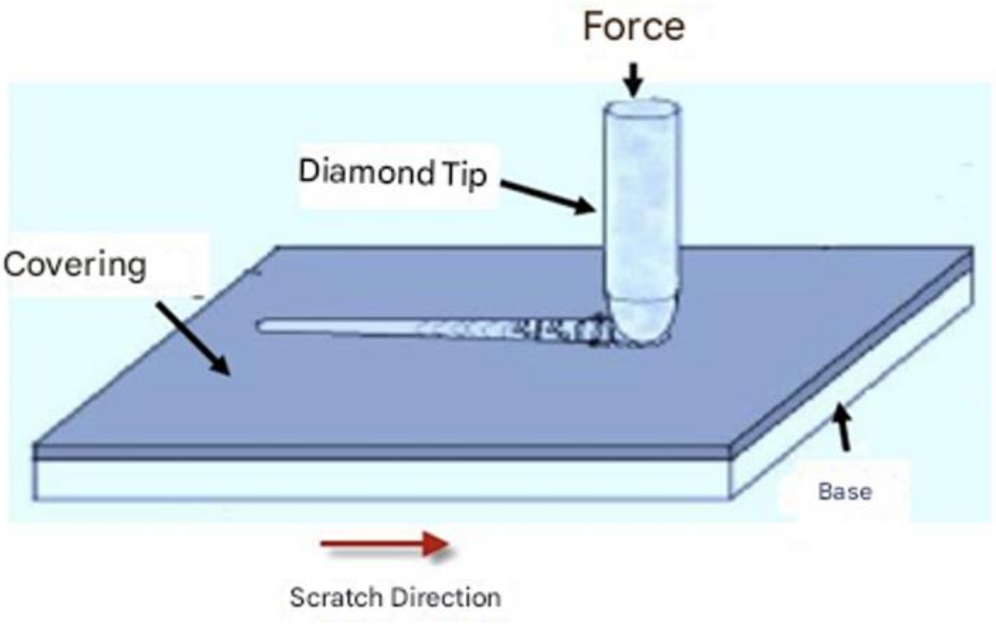

FIGURE 27: Schematic view of the scratch test.

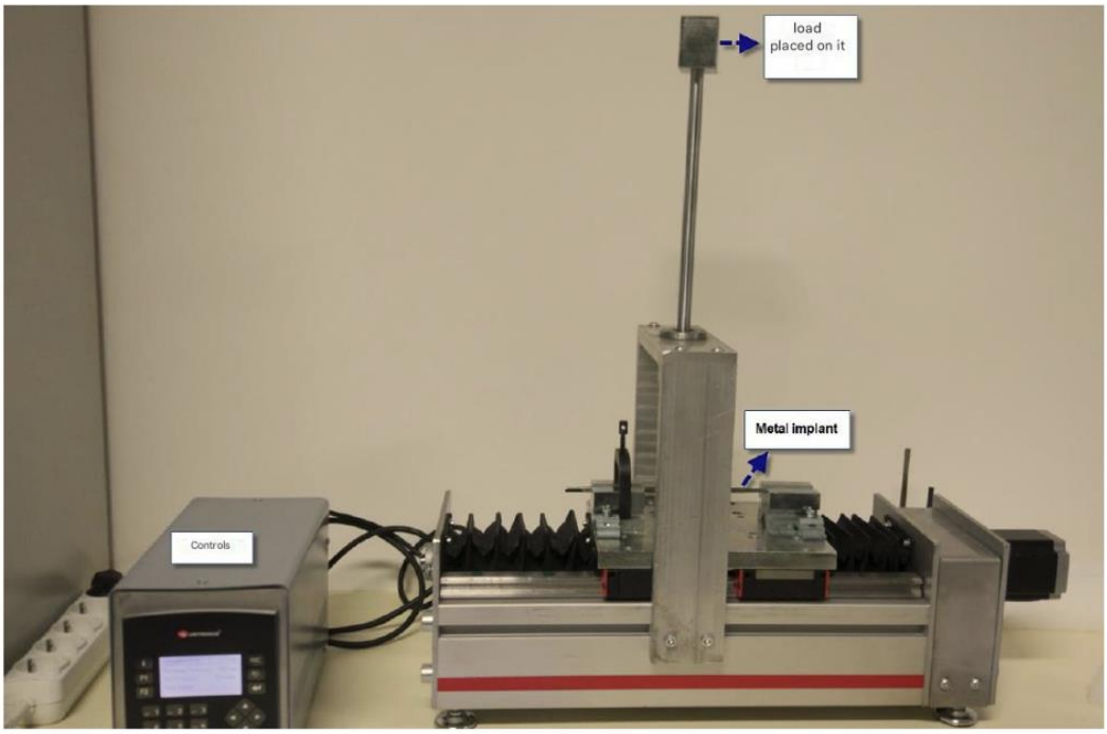

FIGURE 28: Scratch test setup

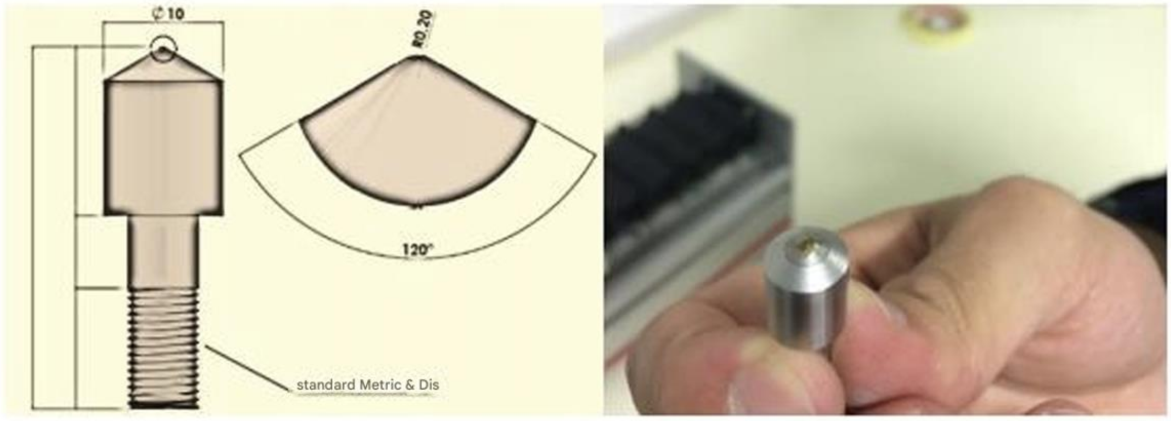

FIGURE 29: Standard diamond tip used for scratch testing.

Optical images of the scratches performed under different flow rates and nozzle distances demonstrated progressive damage modes, with cohesive cracking being more common than adhesive delamination (Figure 30,31).

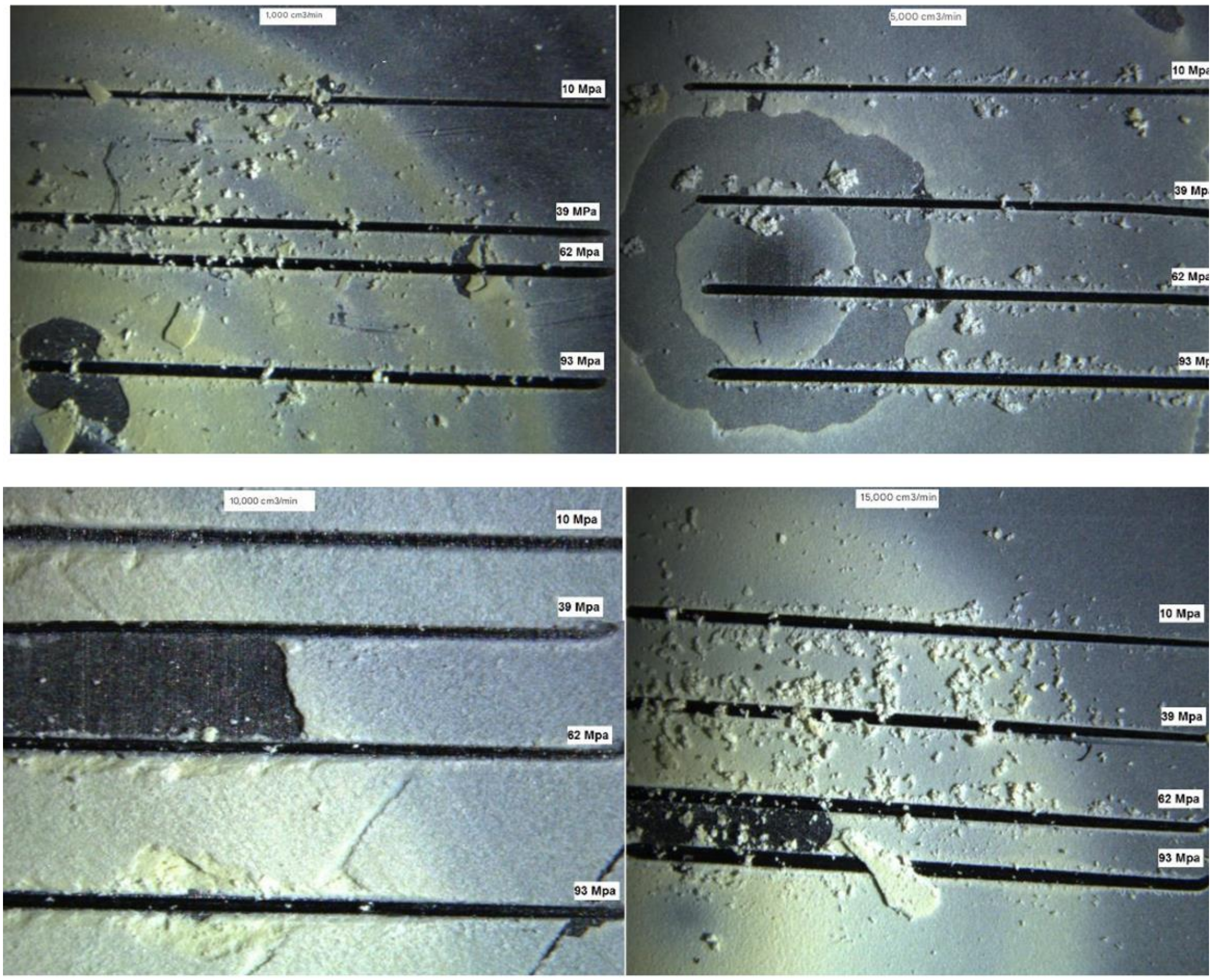

FIGURE 30: Optical images of scratch test coatings applied at different gas flows (1,000, 5,000, 10,000, 15,000 cm3/min)

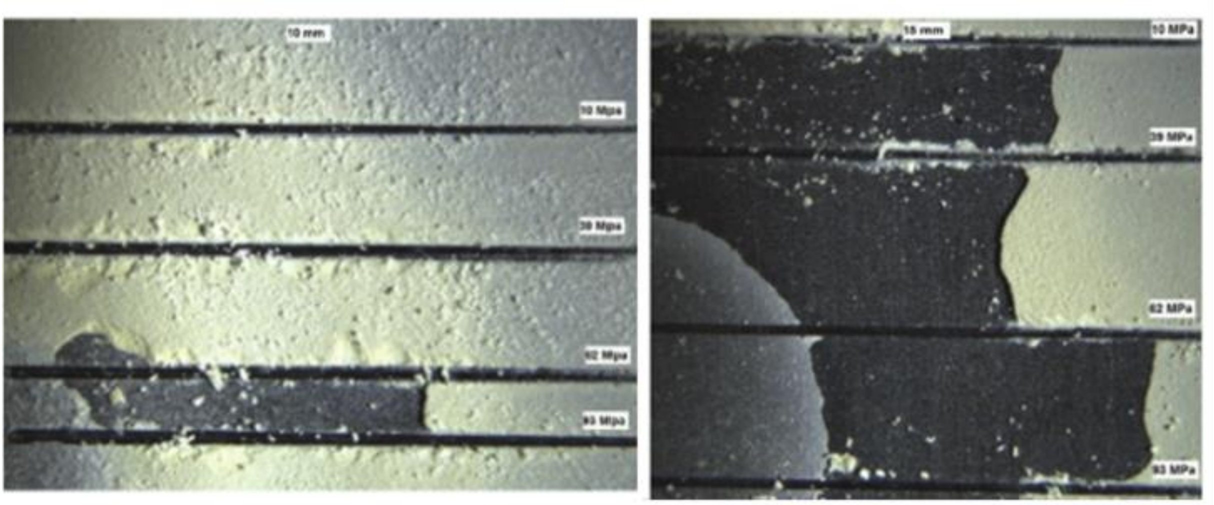

Figure 31. Optical images of scratch tests on coatings deposited at substrate–nozzle distances of 10 and 15 mm

Quantitative data on coating resistance to scratch loads were presented in graphical form (Figure 32-40) and supported by numerical analysis.

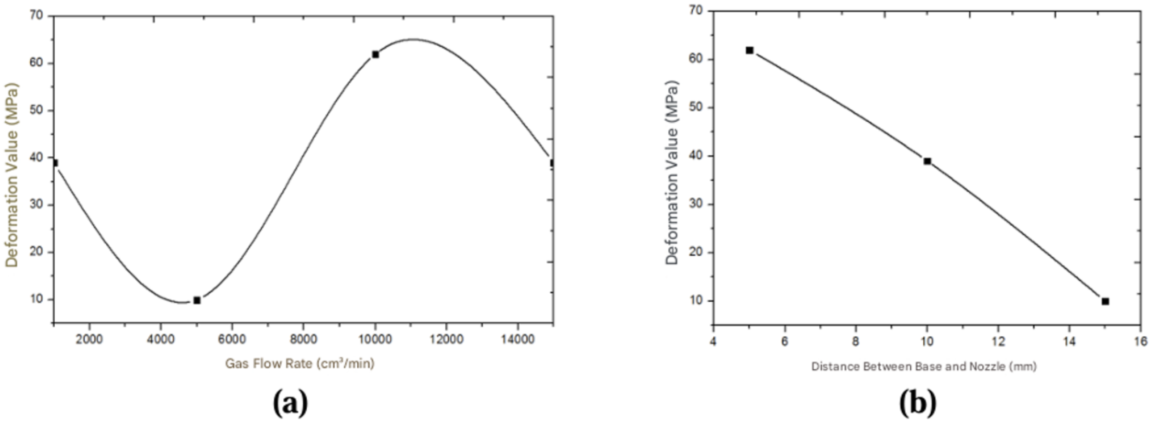

FIGURE 32: a) Effect of gas flow rate and b) Nozzle-to-base distance on coating strength

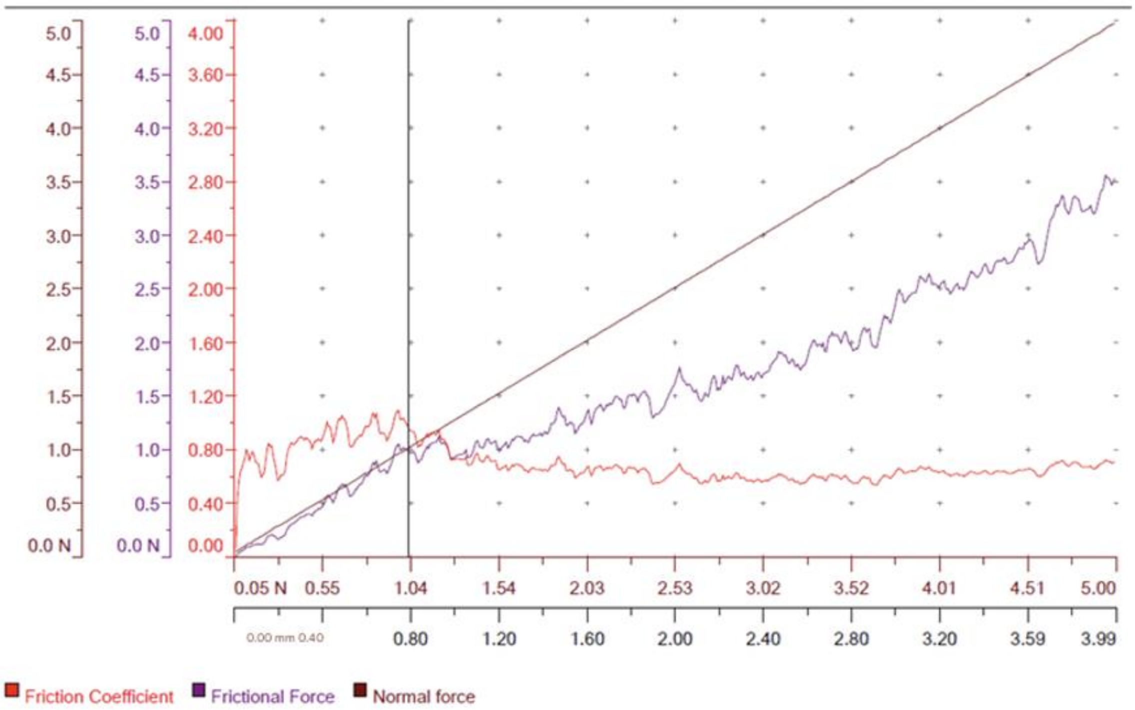

(a)

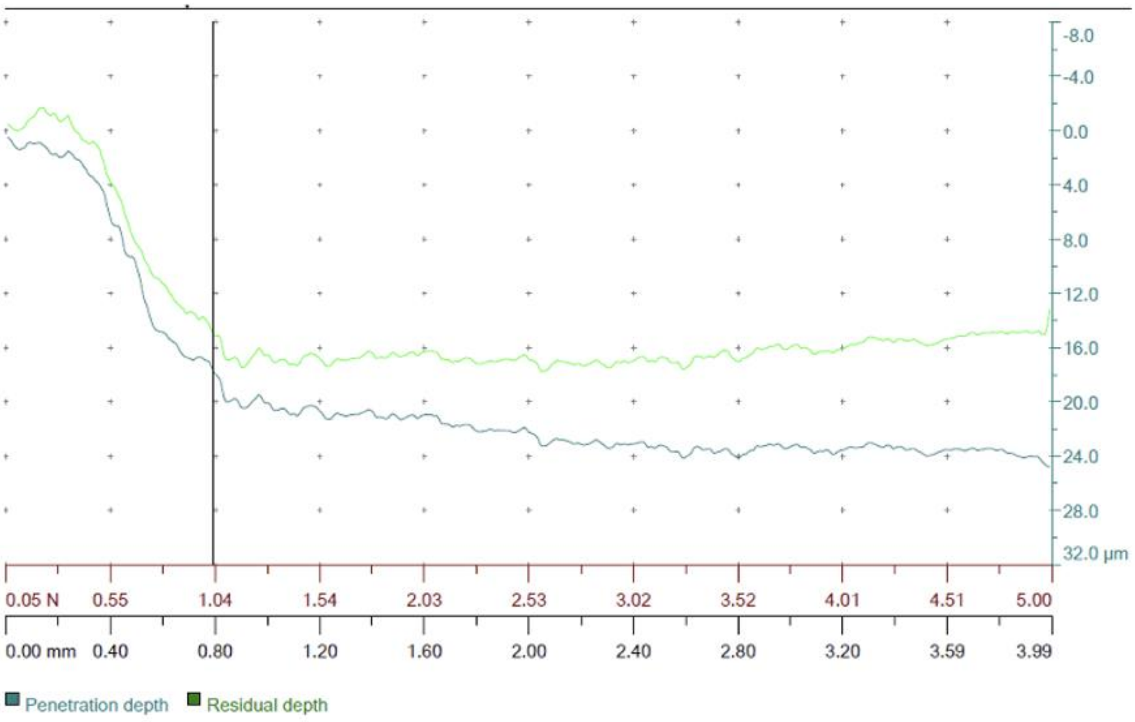

(b)

FIGURE 33: Scratch test results of the coating at 5 mm nozzle-substrate distance and 10,000 cm3/min gas flow a) friction and normal force graph b) penetration graph.

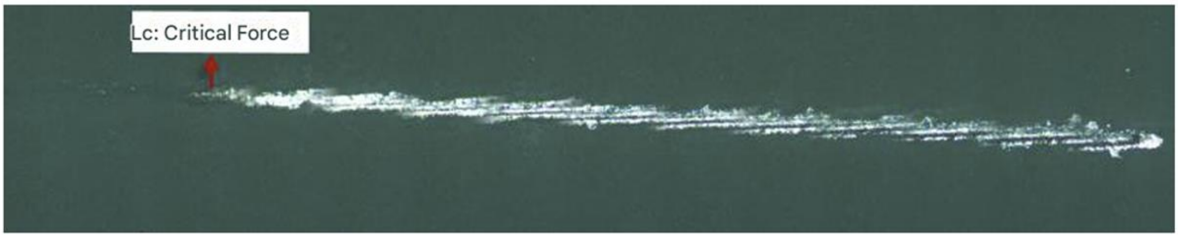

FIGURE 34: Optical image of the scratch.

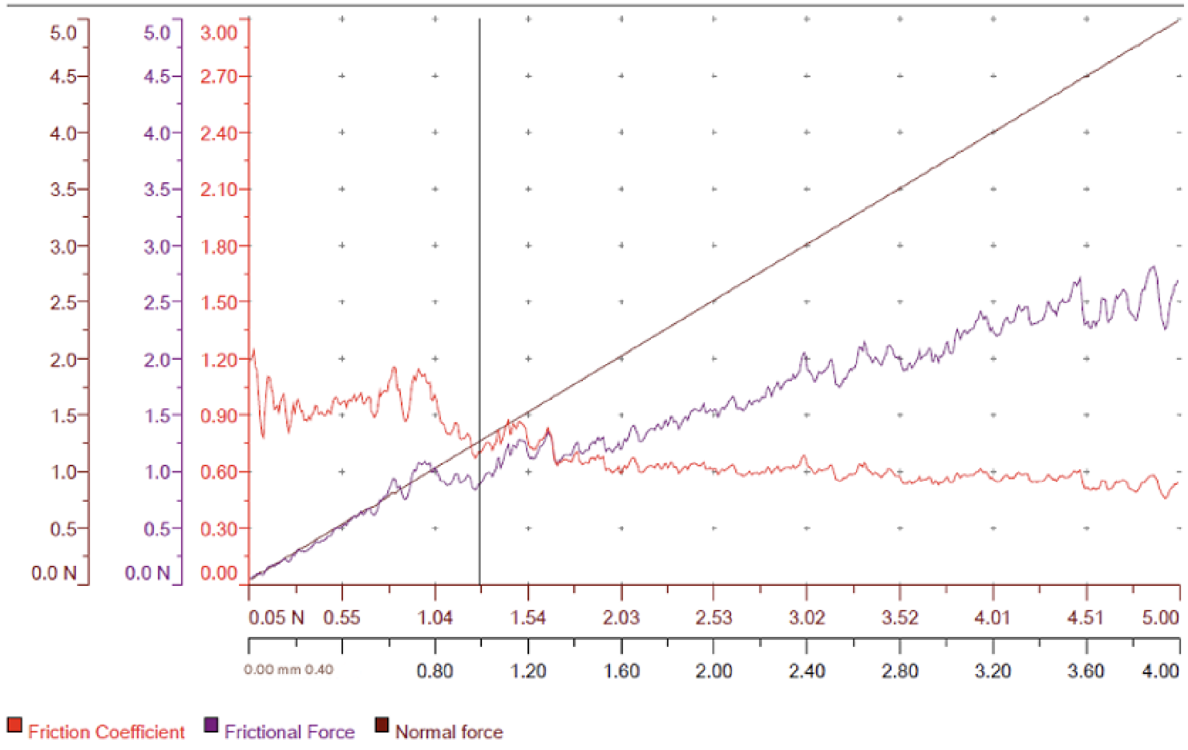

(a)

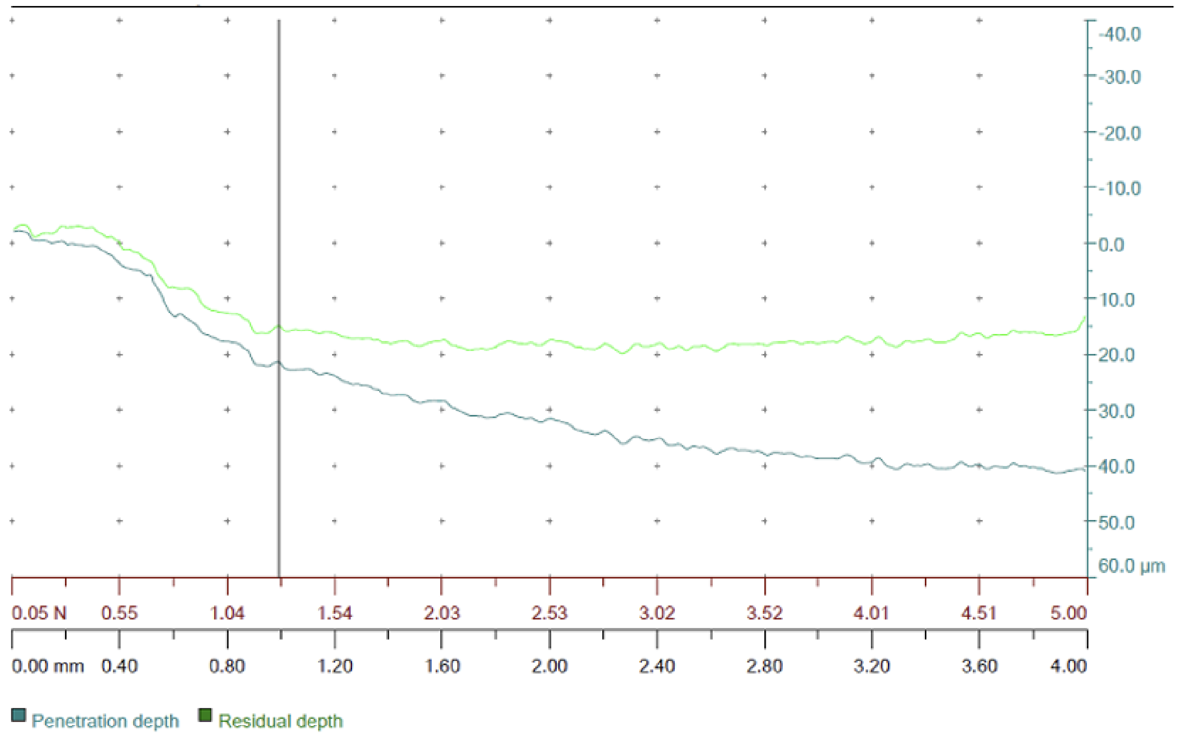

(b)

FIGURE 35: Scratch test results of the coating applied at a 5 mm nozzle-to-substrate distance and a gas flow of 10,000 cm3/min after 50 W plasma application. a) Friction and normal force graph b) Penetration graph.

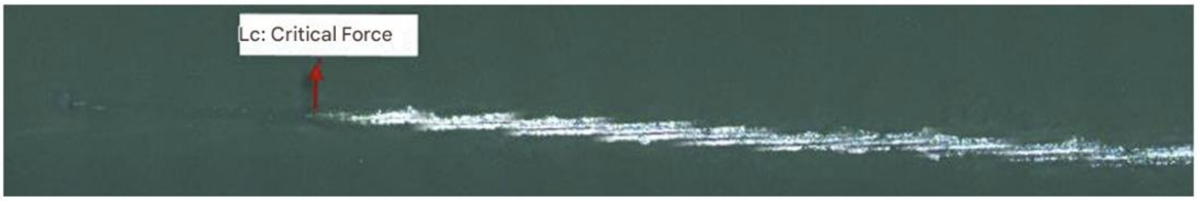

FIGURE 36: Optical image of the scratch created on the coating surface exposed to 50W plasma.

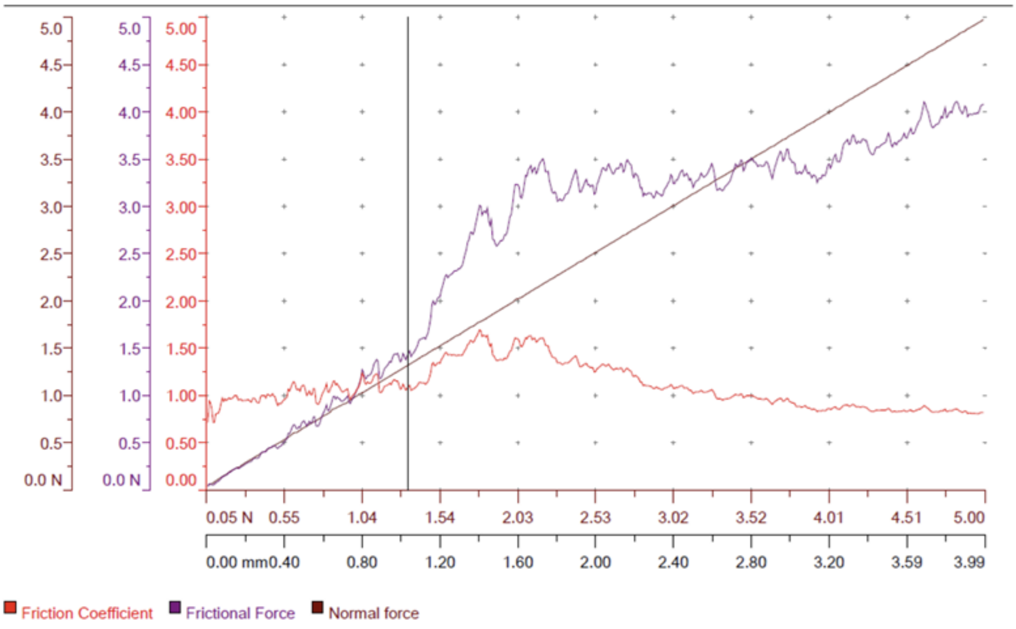

(a)

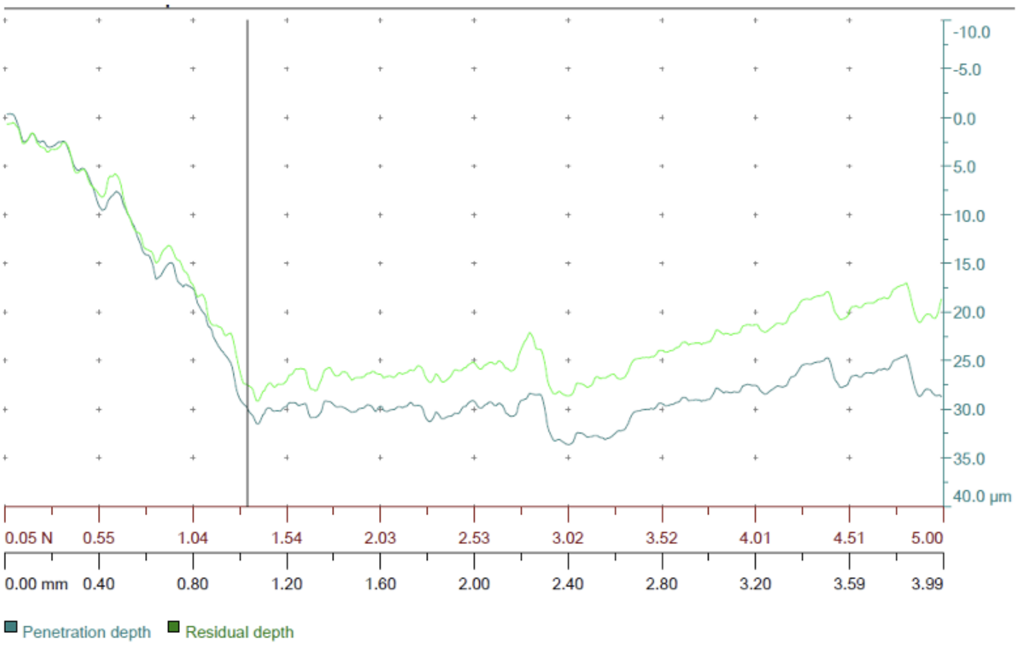

(b)

FIGURE 37: Scratch test results of the coating applied at a 5 mm nozzle-to-substrate distance and a gas flow of 10,000 cm3/min after 100 W plasma application. a) Friction and normal force graph b) Penetration graph.

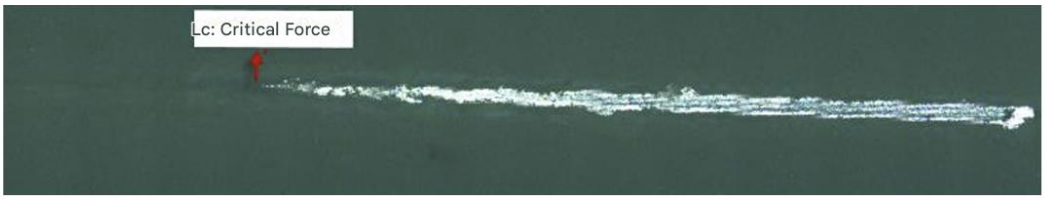

Figure 3.37. Optical image of the scratch created on the coating surface exposed to 100W plasma.

FIGURE 38: Optical image of the scratch created on the coating surface exposed to 100W plasma.

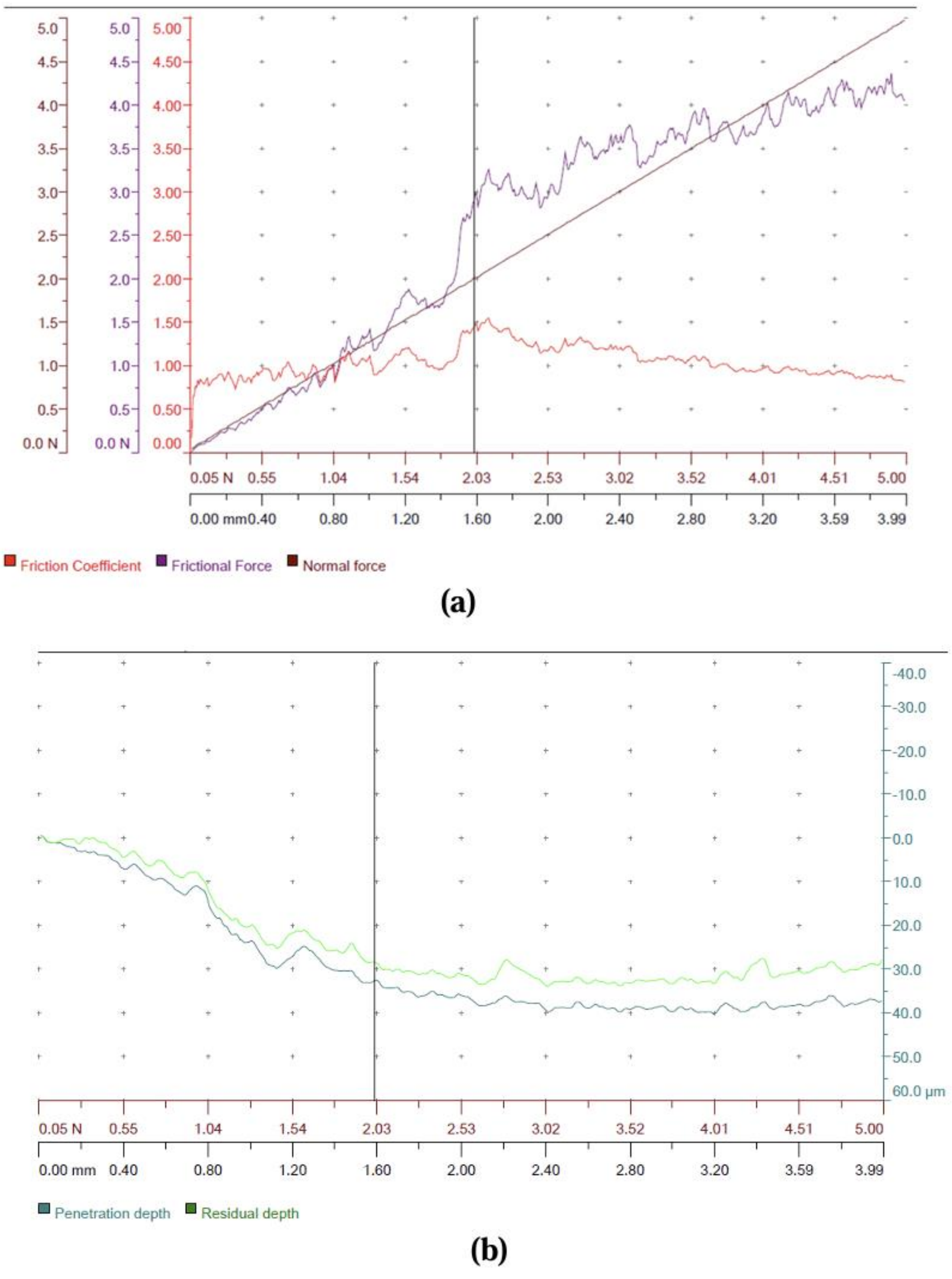

FIGURE 39: Scratch test results of the coating applied at a 5 mm nozzle-to-substrate distance and a gas flow of 10,000 cm3/min after 150 W plasma application. a) Friction and normal force graph b) Penetration graph.

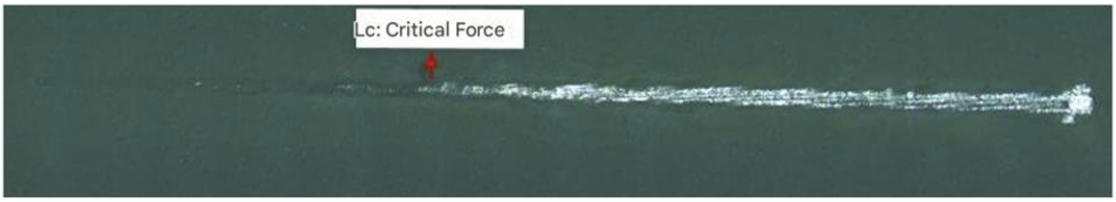

FIGURE 40: Optical image of the scratch created on the coating surface exposed to 150W plasma.

Coating	Substrate	Microscopic observation
Brittle	Ductile	Chipping or fragmentation failures are observed.
Ductile	Ductile	Coating and substrate deform together; buckling failures are observed.
Ductile	Brittle	The coating frequently cracks and tears; conformal failures are observed.
Brittle	Brittle	After tensile cracking, chipping or fragmentation failures are observed.

TABLE 5: Scratch test failure modes according to coating–substrate combinations.

Aerosol deposition parameters	Coating deformation value (MPa)
**Gas flow rate (cm^3/min)**	
1,000	≤39
5,000	≤10
10,000	≤62
15,000	≤39
**Nozzle–substrate distance (mm)**	
5	≤62
10	≤39
15	≤10
**Plasma power (Watt)**	
50	≤10
100	≤39
150	≤62

TABLE 6: Scratch test results according to process parameters.

Parameter	Value
Applied load	Progressive
Initial load (N)	0.05

Parameter	Value
Final load (N)	5
Loading rate (N/min)	4.95
Speed (mm/min)	4
Length (mm)	4
Indenter type	Rockwell
Indenter diameter (μm)	100

TABLE 7: Experimental conditions of the micro-scratch test.

Overall, the AD process enabled the deposition of nanoscale CaP–Ag coatings on metallic implants with high adhesion and a controlled microstructure. The combination of fine particle size (≤0.2 μm), gas flow between 5000 and 10,000 cm^3/min, a nozzle distance of 10 mm, and RF plasma assistance at 100 W yielded the most favorable results, as confirmed by microstructural, roughness, and scratch test data.

4. Electrospray Deposition of Metal Implants

Electrospray deposition was performed to generate nanoscale silver-ion doped calcium phosphate coatings on metallic implant surfaces. The general setup of the electrospray system, including the syringe pump, high-voltage power supply, nozzle, and substrate holder, is shown schematically in Figure 41. Photographs of the coated external fixators, intramedullary nails, and hip prostheses following deposition are presented in Figure 42, demonstrating the applicability of the method to implants with complex geometries.

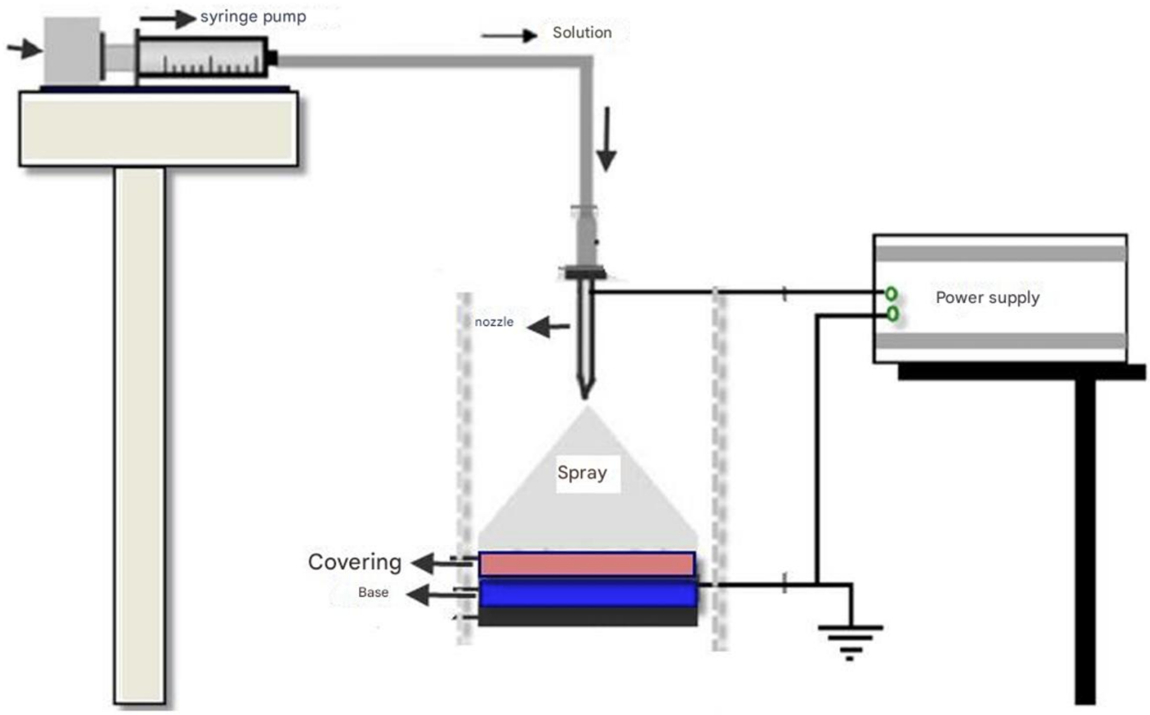

FIGURE 41 : Schematic representation of the electrospray method.

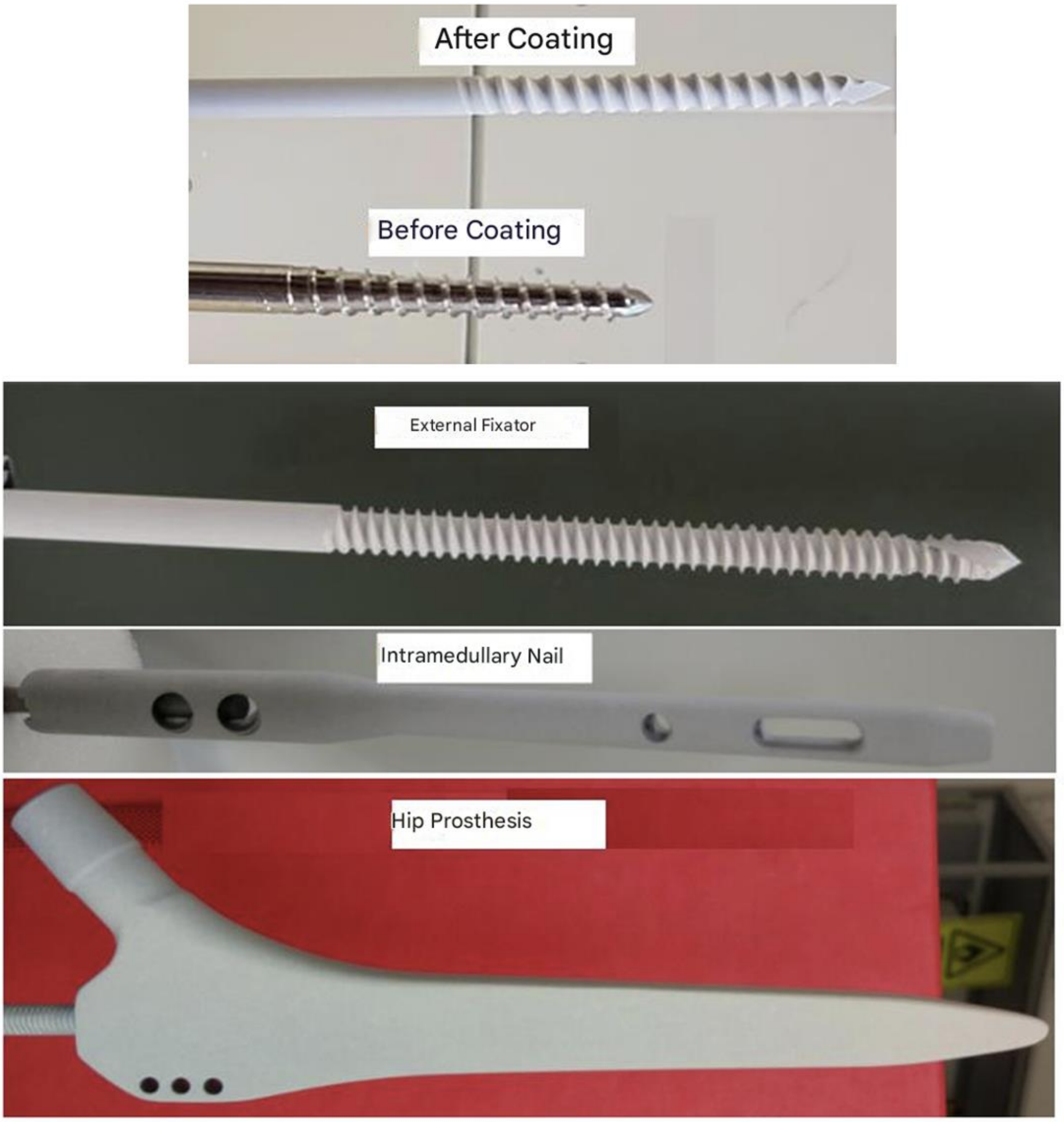

FIGURE 42: Image of metal implants coated with the electrospray method.

For the preparation of stable suspensions, silver-doped calcium phosphate powders were dispersed in methanol–water mixtures at concentrations ranging from 1 to 5 wt.% (Tablo 4.3). Polyvinylpyrrolidone (PVP, 1 wt.%) was added to prevent agglomeration, and the suspensions were ultrasonicated for 30 minutes. Sedimentation behavior was visually assessed (Figure 43), and zeta potential measurements confirmed the electrostatic stability of the dispersions (Figure 44-46).

**Ag^{+}-doped calcium phosphate**			**6P57 Bioglass**
**Water (H_2O)**	**Methanol (CH_3OH)**	**Ethanol (C_2H_5OH)**	**Metanol (CH3OH)**
1%	1%	1%	1%
3%	3%	3%	
5%	5%	5%	

TABLE 8: Weight concentrations (%) of suspensions prepared for coating.

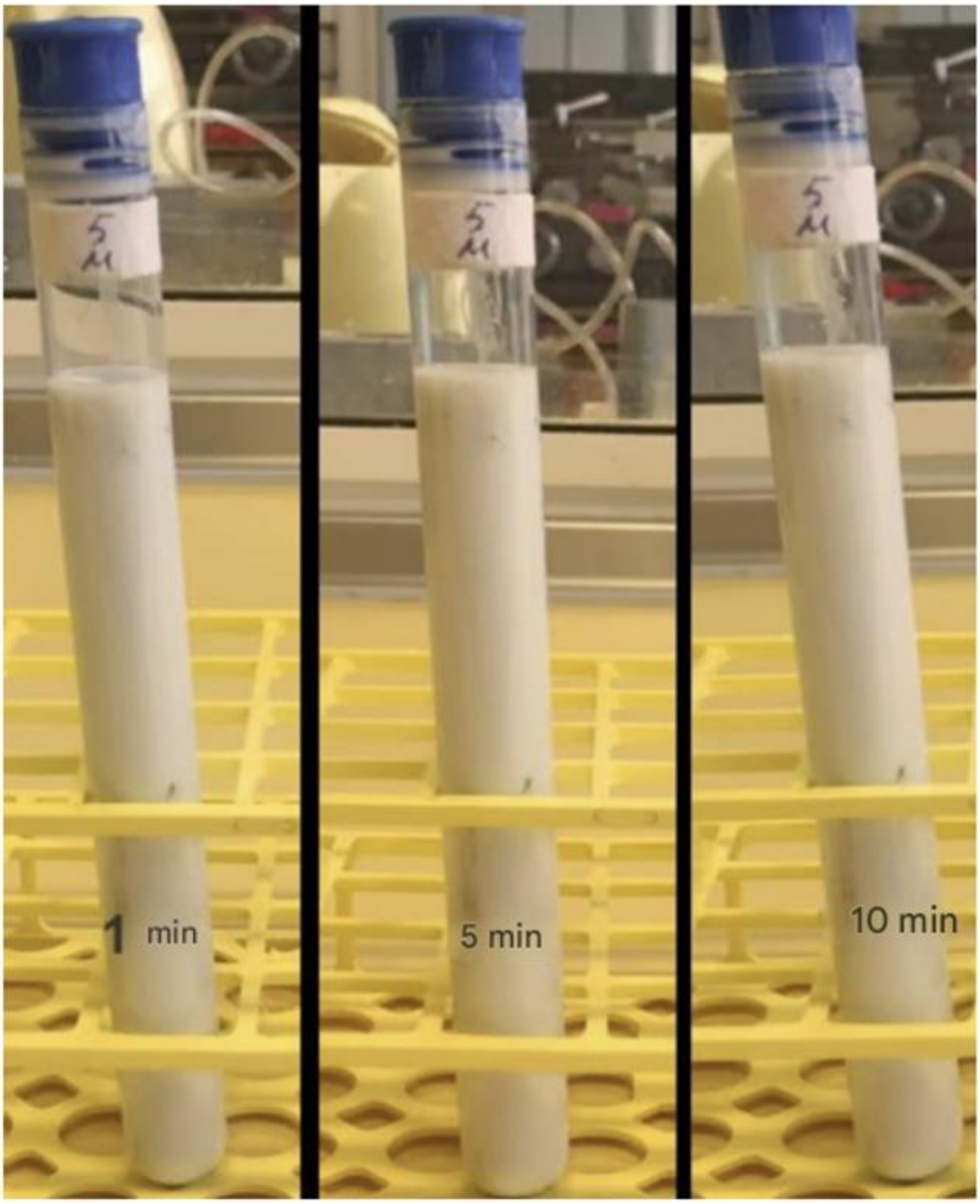

FIGURE 43: Sedimentation test of suspension prepared from 6P57 bioglass.

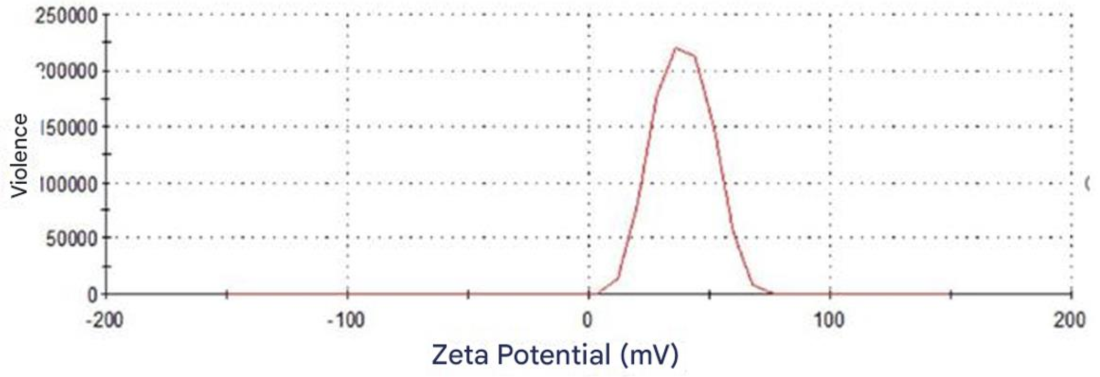

FIGURE 44: Zeta potential curve of the suspension prepared in 1% methanol.

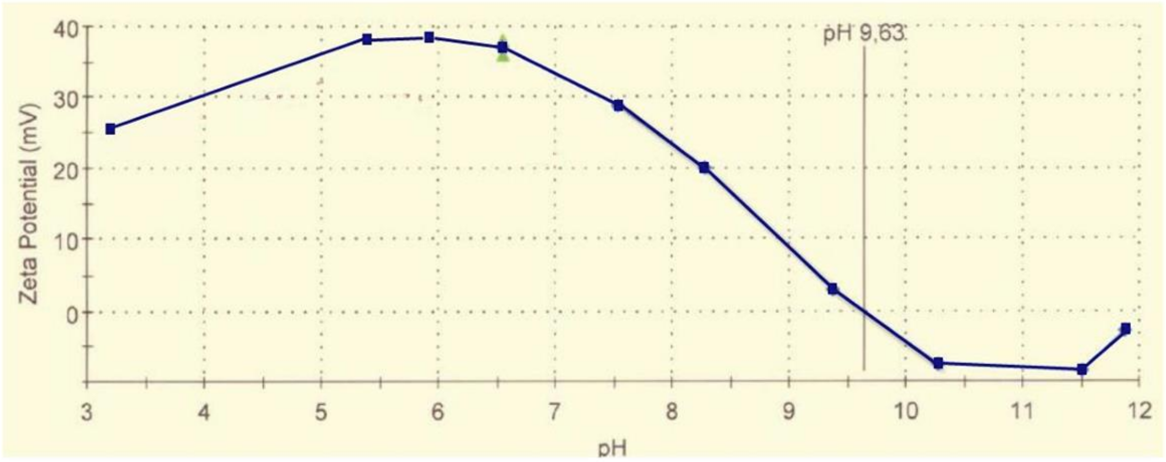

FIGURE 45: Zeta Potential curve of the prepared suspension depending on Ph.

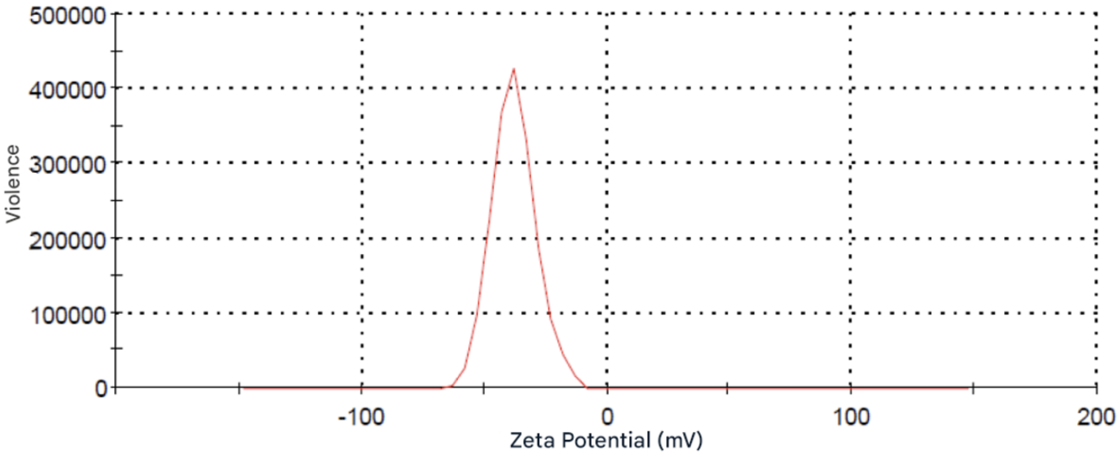

FIGURE 46: Zeta potential curve of the suspension prepared from bioglass.

Coating was performed under optimized electrospray conditions: a flow rate of 0.3–0.5 mL/h, an applied voltage of 10–15 kV, a nozzle–substrate distance of 30–50 mm, and deposition times between 20 and 40 minutes. Coating parameters and their effects on deposition efficiency are summarized in Table 4.4**.** After deposition, implants were subjected to sintering in an RF induction furnace (Figure 47) at 600–800 °C under argon atmosphere for 1–2 hours (Tablo 4.5). This post-treatment step enhanced adhesion strength and promoted crystallization of the CaP phases.

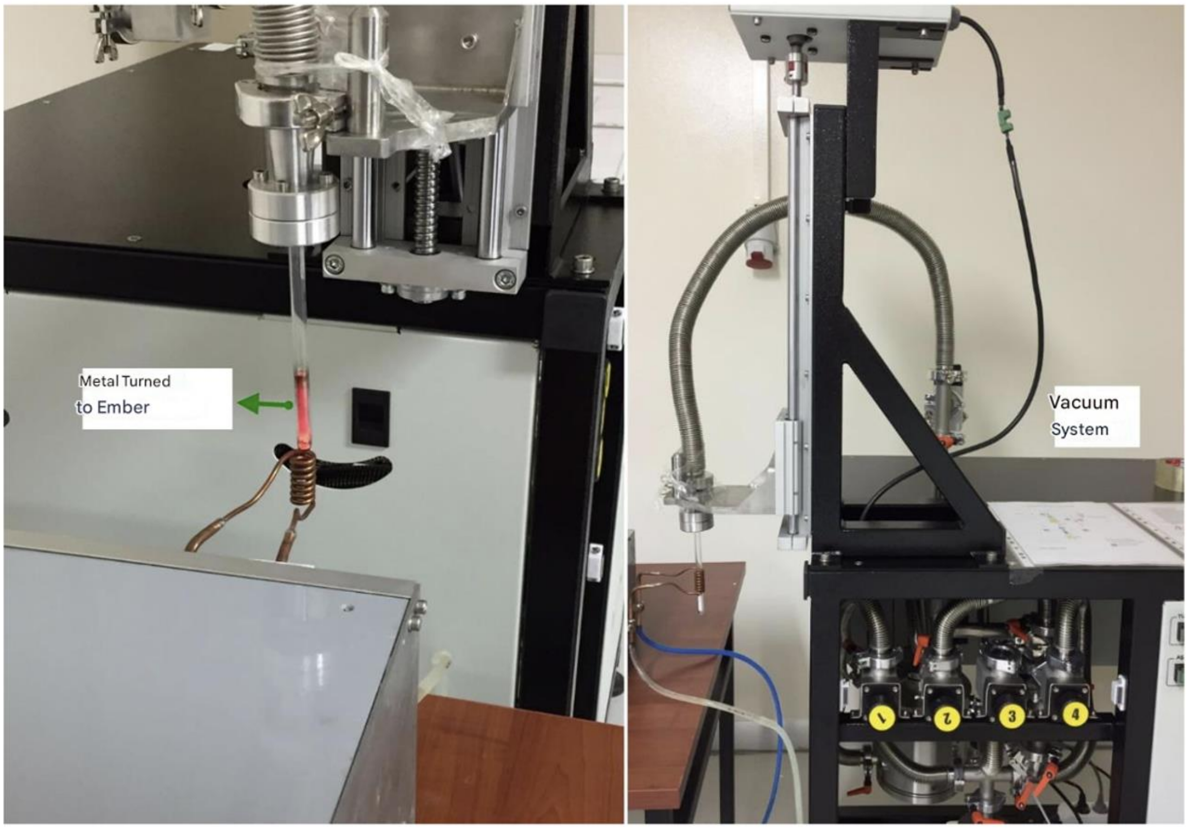

FIGURE 47: Image of the RF sintering technique and the vacuum system

**Implant group**	**Applied voltage (kV)**	**Suspension flow rate (mL/s)**	**Nozzle–substrate distance (cm)**	**Coating time (min)**	**Implant movement and rotation speed (rpm)**
External fixator	14	5	4	Variable	15
Intramedullary nail	14	5	4	Variable	15
Hip prosthesis	14	5	4	Variable	15

TABLE 9: Electrospray coating parameters of metal implants.

Microstructural evaluation demonstrated the formation of continuous, uniform coatings across implant surfaces. SEM images revealed nanoscale layers with thicknesses between 200 and 500 nm and intimate bonding with the substrate (Figure 48,49). Cross-sectional SEM confirmed that no delamination occurred, while AFM measurements showed surface roughness values in the range of 0.3–0.6 µm (Table 10).

**Implant material**	**Implant type**	**Surface roughness before coating (µm)**	**Surface roughness after coating (µm)**
316 L Stainless steel	External fixator	0.082	0.455
Ti6Al4V	Intramedullary nail	0.328	0.476
Ti6Al4V	Hip prosthesis	0.256	0.389

TABLE 10: urface roughness values before and after coating.

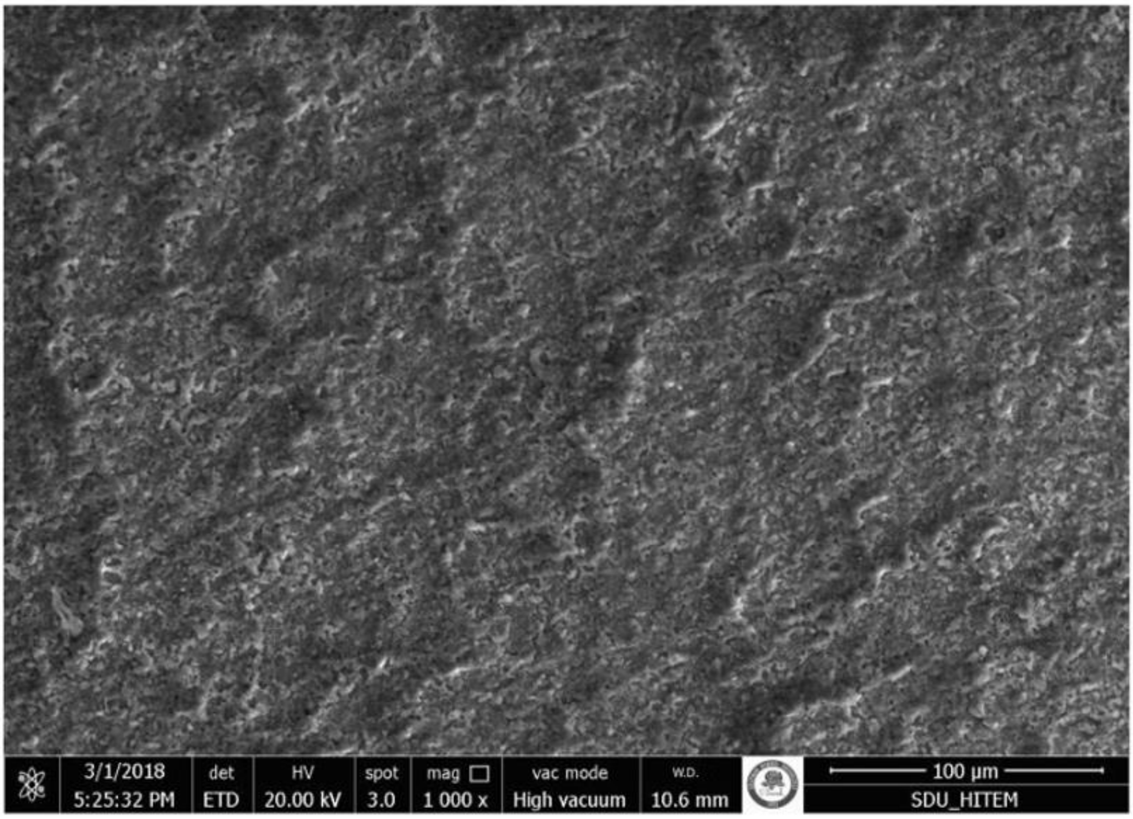

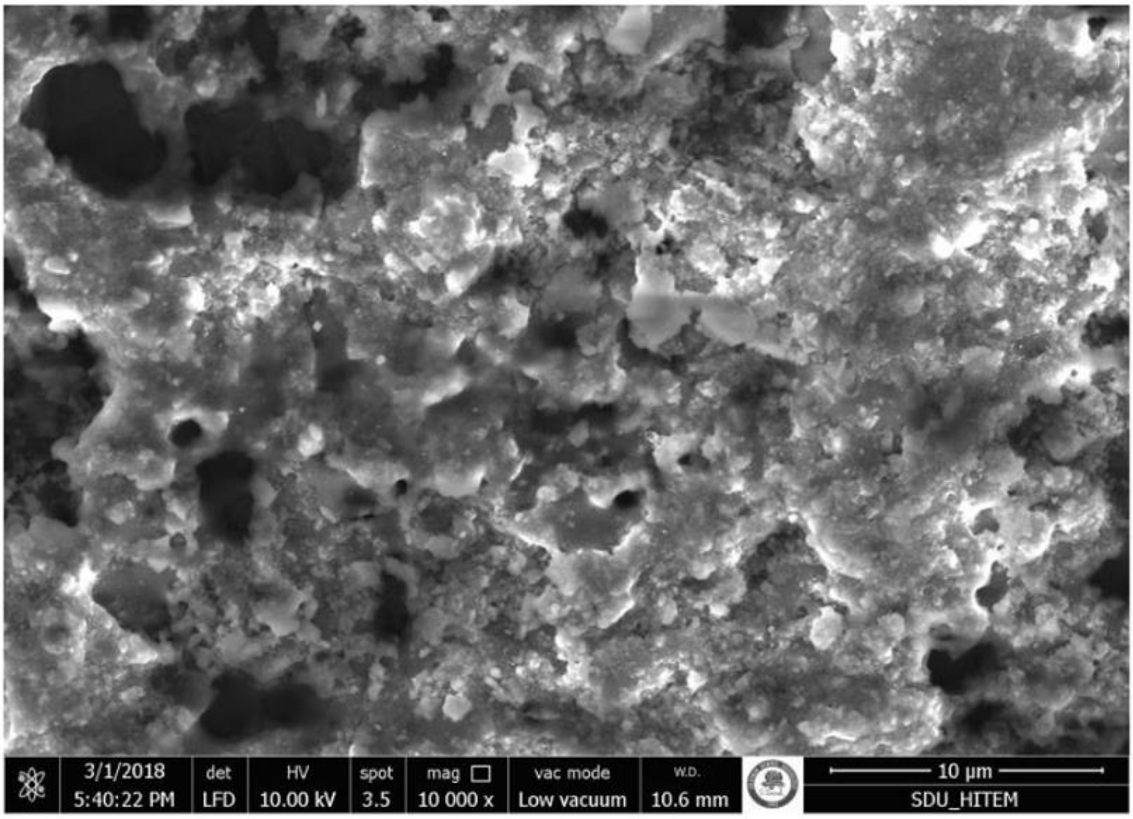

FIGURE 48: Microstructure of the coating on the metal implant surface after heat treatment.

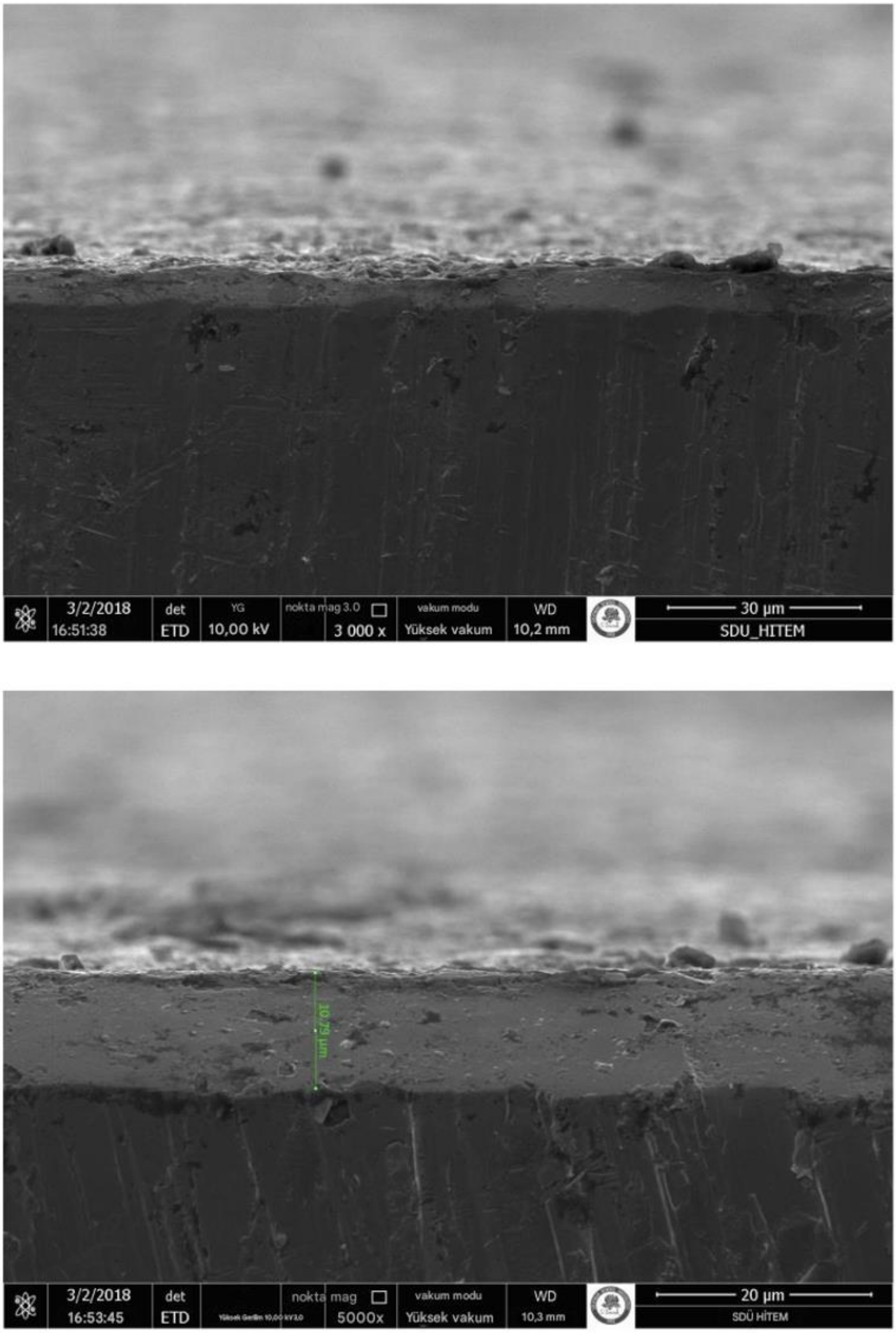

FIGURE 49: SEM images taken from the interface of the metal implant and the coating.

Chemical analysis with EDX confirmed the uniform distribution of calcium, phosphorus, oxygen, and silver throughout the coating (Figure 50). XRD patterns showed that both hydroxyapatite and tricalcium phosphate phases were present, depending on the initial powder composition and processing parameters (Figures 51,52).

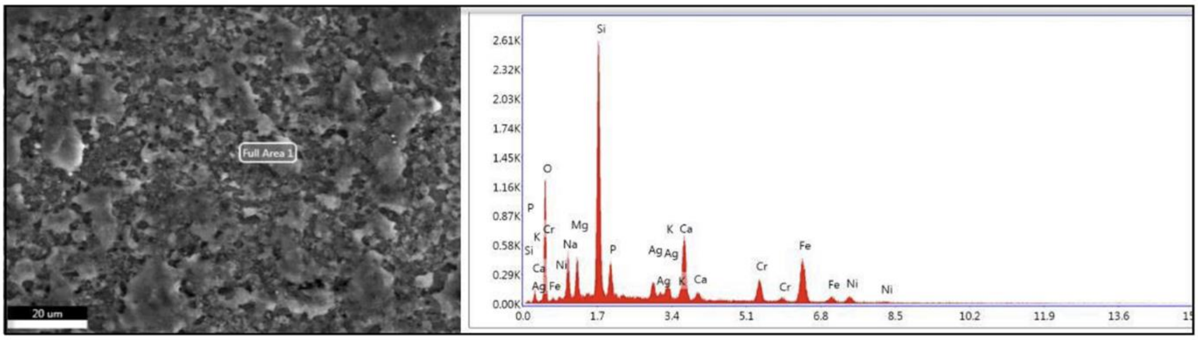

FIGURE 50: EDX analysis result from the heat-treated coating surface.

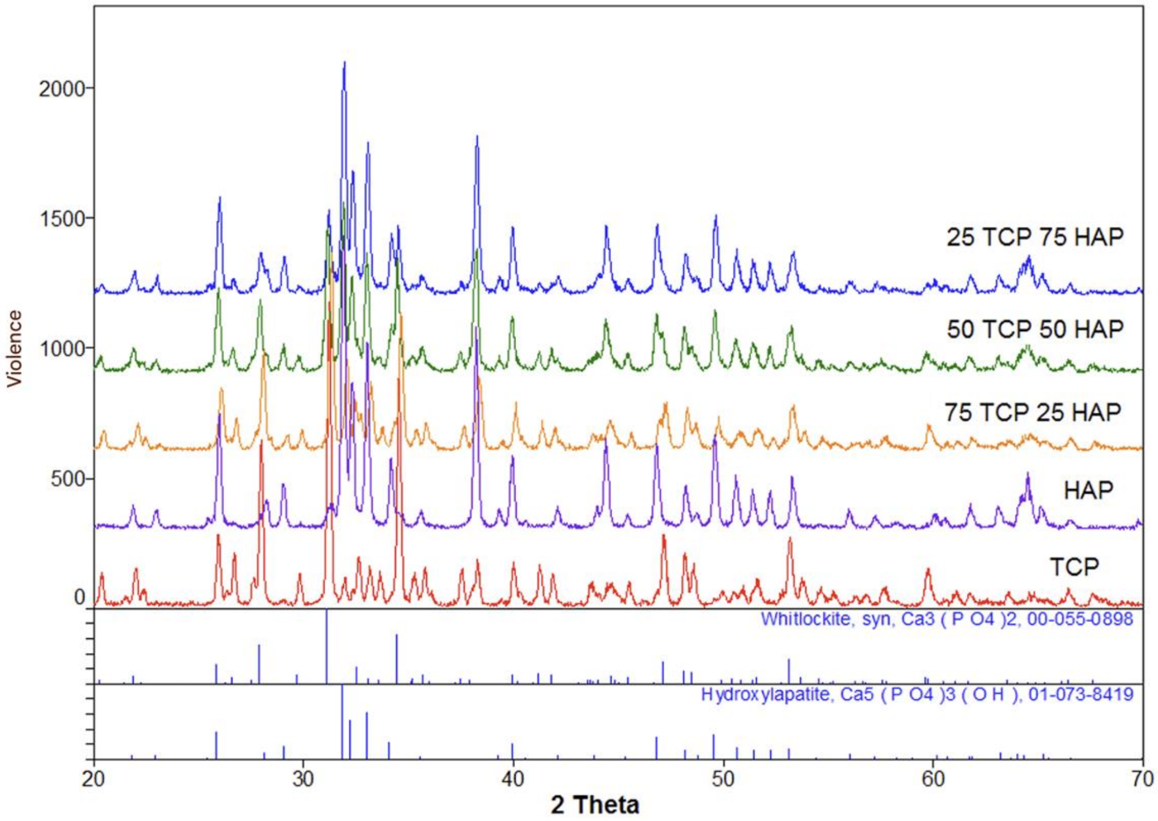

FIGURE 51: HAP and TCP phases prepared at different ratios.

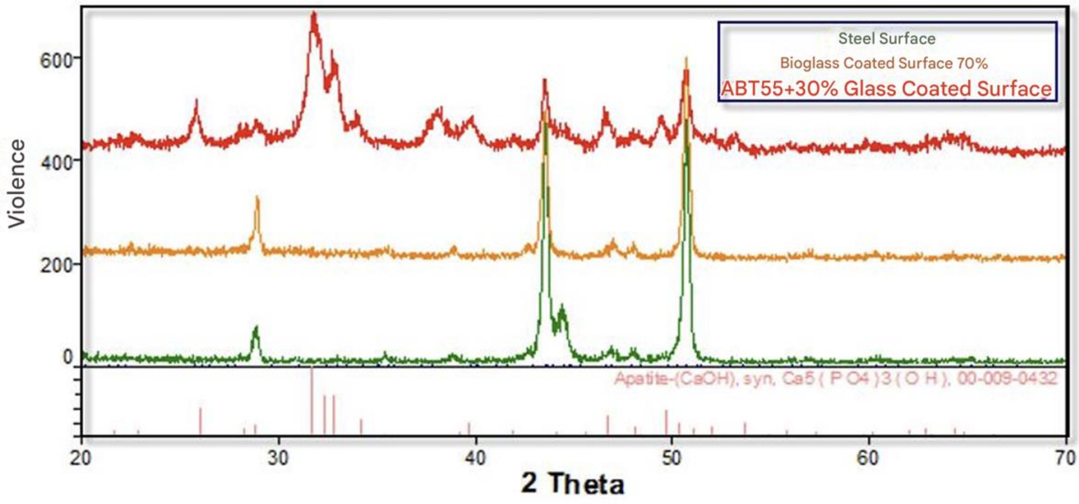

FIGURE 52: XRD lattice patterns obtained from the metal surface, the bioglass-coated surface, and the bioglass+HAP-coated surface.

Mechanical characterization provided additional validation. Mechanical properties (including microhardness) before and after heat treatment are summarized in Table 11. Micro-scratch testing on steel and titanium alloy substrates is shown in Figures 53,54. The test yielded friction/normal force and penetration curves (Figure 55), while Figure 56 presents the optical image of the scratch track. The experimental conditions for the micro-scratch tests are listed in Table 12.

**Implant type**	**Elastic modulus (GPa) Before heat treatment**	**Elastic modulus (GPa) After heat treatment**
External fixator	145.85	124
Intramedullary nail	32.8	34.77
Hip prosthesis	27.8	26.4

TABLE 11: Mechanical properties of metal implants before and after heat treatment

Parameter	Value
Applied load	Progressive
Initial load (N)	0.05
Final load (N)	5
Loading rate (N/min)	4.95
Speed (mm/min)	4
Length (mm)	4
Indenter type	Rockwell
Indenter diameter (μm)	100

TABLE 12: Experimental conditions of the micro-scratch test.

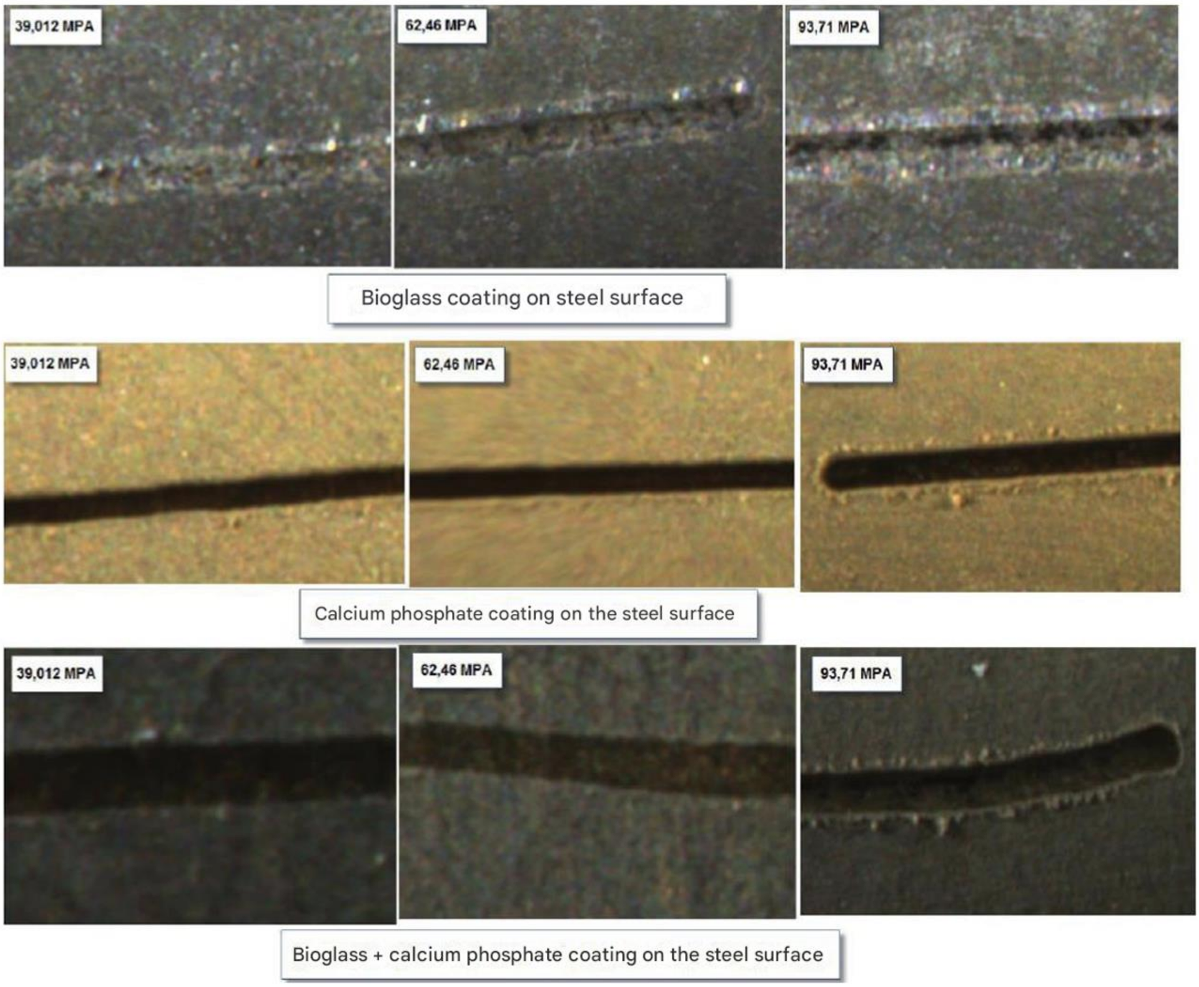

FIGURE 53:Scratch test images of the coating applied to the steel surface.

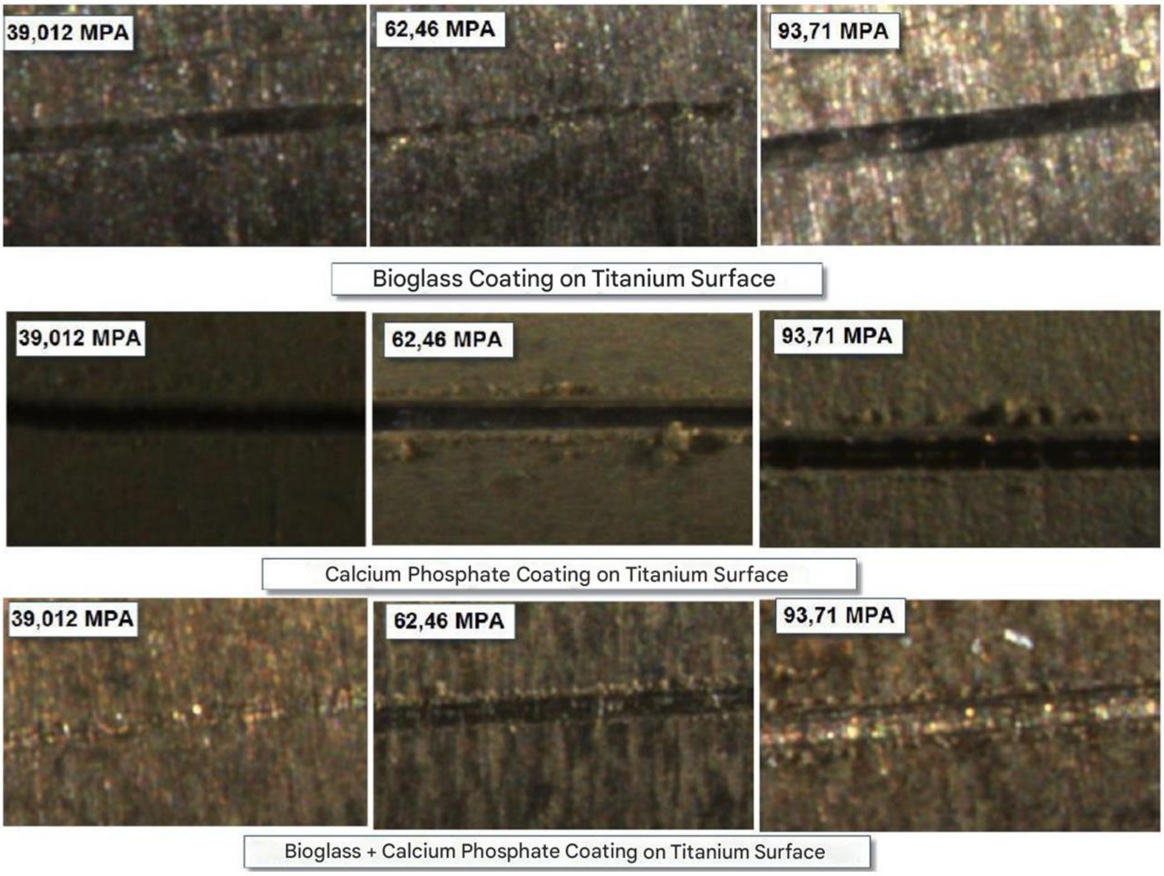

FIGURE 54: Scratch test images of the coating applied to the titanium alloy surface.

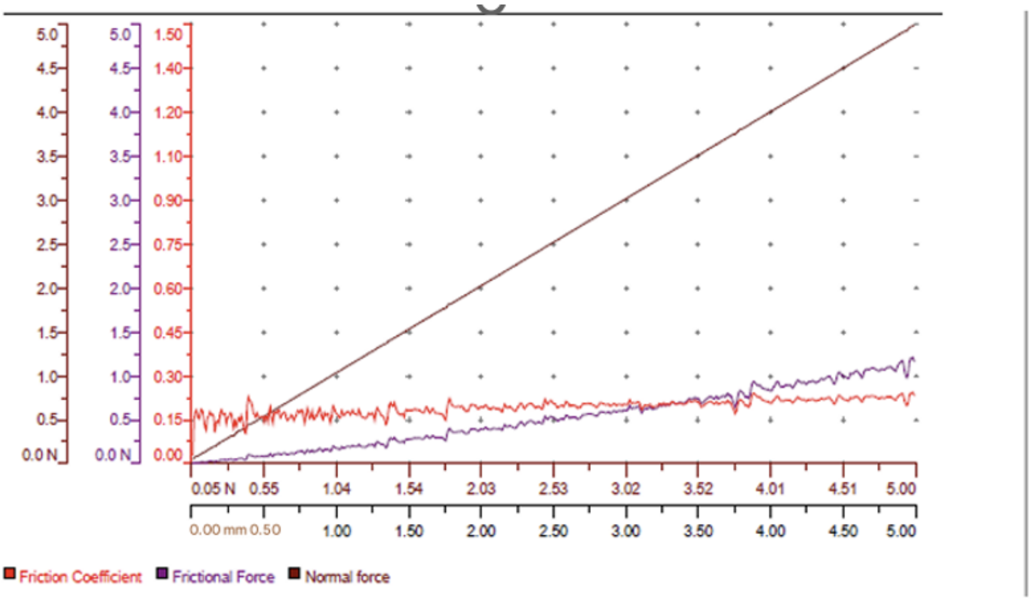

(a)

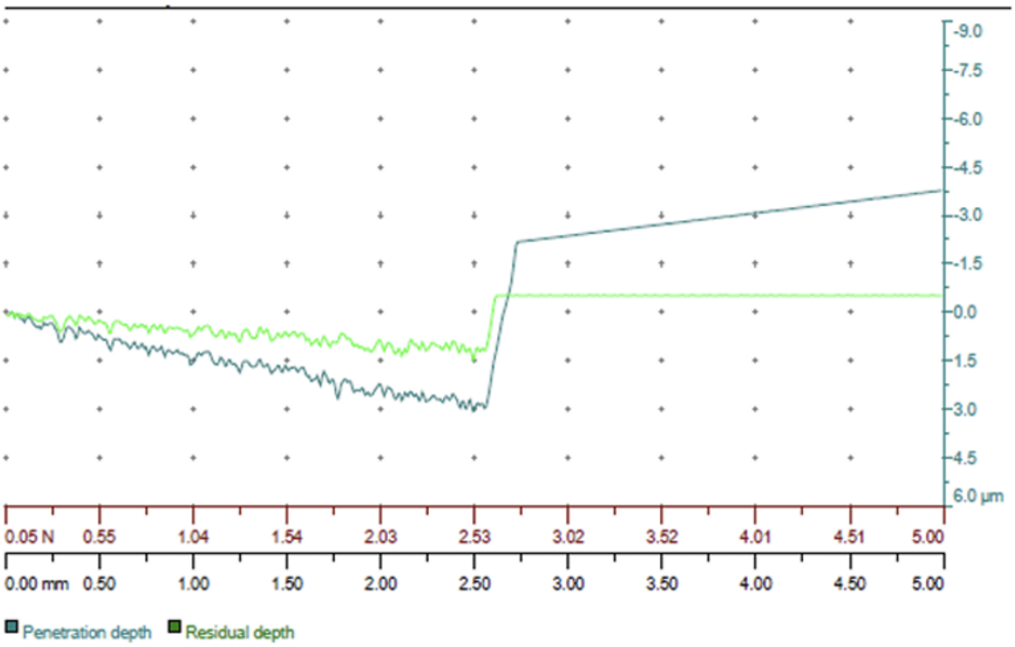

(b)

FIGURE 55: a) Friction and normal force graph b) Penetration graph of the coating on the external fixator surface.

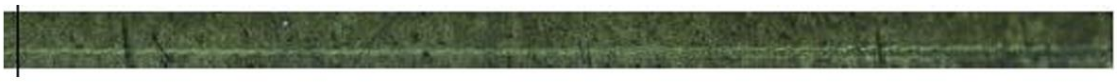

FIGURE 56: Optical image of the drawn test.

Altogether, electrospray deposition enabled the formation of homogeneous nanoscale coatings with strong adhesion, stable crystalline phases, and low surface roughness. The method proved versatile for different implant geometries and, after sintering at 700 °C, produced coatings that were mechanically robust and chemically stable.

5. In-vitro Dissolution and Stability Tests

The dissolution behavior and long-term stability of the coatings were evaluated under both static and dynamic conditions in human plasma at 37 °C. The experimental setups for these tests are presented in Figure 57, showing the immersion system for static assays and the circulation loop for dynamic assays. Measurements included pH monitoring, mass loss determination, surface morphological evaluation, and chemical composition analysis over 16 weeks. Data are summarized in Tables 13-15**.**

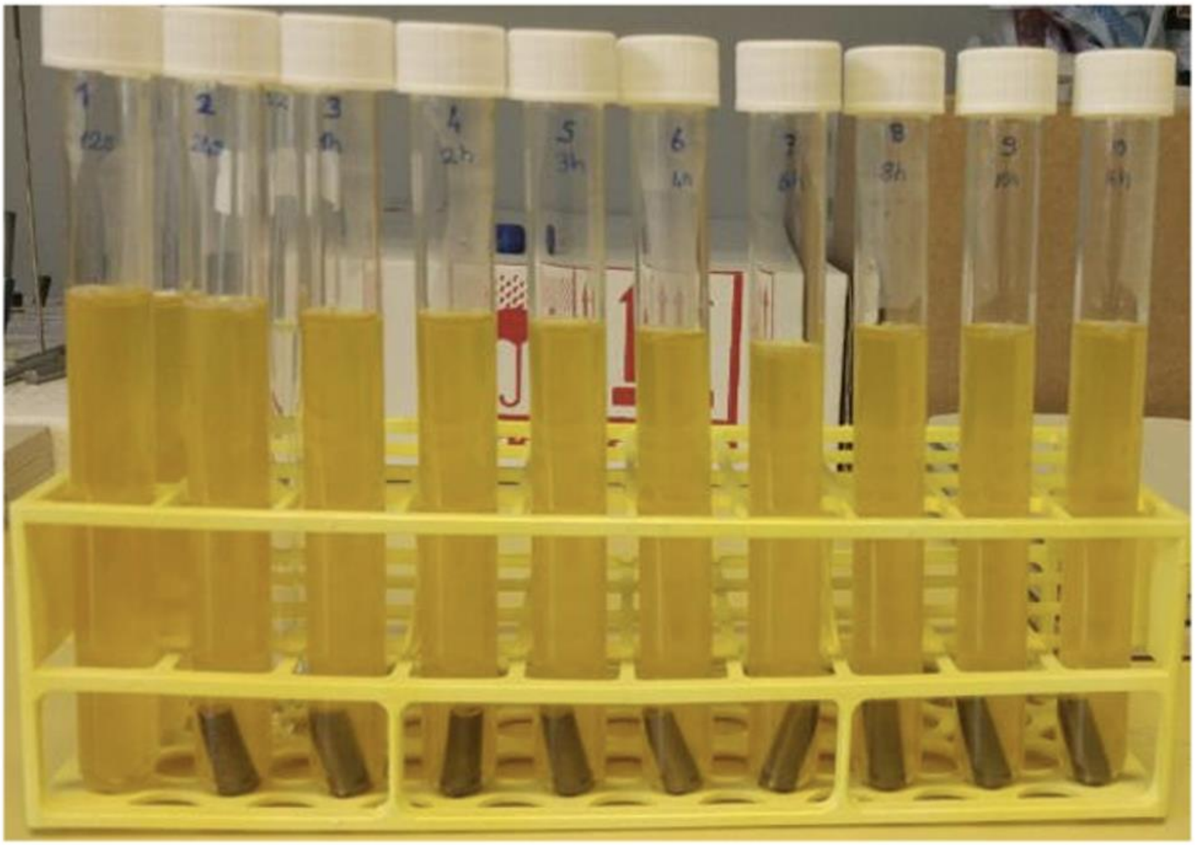

FIGURE 57: Initial image of the static plasma experiment.

**Time**	**pH (plasma)**			**Metal implant + coating weight (g)**			
	Initial	Final	Difference	Initial	Final	Difference	% Change
12 h	7.47	7.55	0.08	4.2494	4.249	0.0004	0.009
24 h	7.47	7.89	0.42	4.5117	4.5111	0.0006	0.013
1 week	7.47	7.47	0.00	4.211	4.2099	0.0011	0.026
2 weeks	7.47	6.35	-1.12	4.6487	4.6474	0.0013	0.028
3 weeks	7.47	6.18	-1.29	4.6447	4.643	0.0017	0.037
4 weeks	7.47	6.74	-0.73	4.171	4.1604	0.0017	0.038
6 weeks	7.47	6.00	-1.47	4.5398	4.5381	0.0017	0.037
8 weeks	7.47	8.25	0.78	4.6113	4.6096	0.0017	0.037
10 weeks	7.47	7.99	0.52	4.6273	4.6258	0.0015	0.032
16 weeks	7.47	8.45	0.98	4.3173	4.3159	0.0014	0.032

TABLE 13: Changes in plasma pH value and implant + coating weight during the static dissolution test.

**Time**	**pH**																		
	Initial	1	2	3	4	5	6	7	8	9	10	11	12	13	14	15	16	17	18
12 h	7.47	7.64																	
24 h	7.47	7.65																	
1 week	7.47	7.15	6.20																
2 weeks	7.47	7.14	7.42	6.63	5.99														
3 weeks	7.47	6.55	6.19	6.20	6.16	6.17	6.45												
4 weeks	7.47	6.90	6.96	6.15	6.46	6.31	6.25	6.35	6.30										
6 weeks	7.47	6.46	6.05	5.87	6.07	5.98	6.20	5.92	6.09	6.56	5.81	5.79	5.72						
8 weeks	7.47	7.51	7.08	6.58	6.66	6.38	6.17	6.36	7.43	6.05	6.30	5.88	5.54	5.50	5.49				
10 weeks	7.47	7.16	6.25	6.36	5.96	6.09	5.89	6.13	6.00	5.98	6.09	5.98	5.90	6.00	6.04	5.88	6.15	6.26	
16 weeks	7.47	7.94	6.42	6.41	5.80	6.24	6.14	6.62	6.61	5.80	6.07	6.00	5.74	5.86	6.25	5.78	6.37	5.82	6.20

TABLE 14: Changes in plasma pH values after the dynamic dissolution test.

Time	Weight (g)			
	Initial	Final	Difference	% Change
12 h	2.2883	2.288	0.0006	0.0262203
24 h	2.3888	2.388	0.0004	0.0167448
1 week	2.3418	2.341	0.0008	0.0341618
2 weeks	2.2444	2.244	0.0009	0.0400998
3 weeks	2.1949	2.194	0.0005	0.0227801
4 weeks	2.1378	2.137	0.0009	0.0420994
6 weeks	2.3370	2.336	0.0010	0.0427899
8 weeks	2.2044	2.204	0.0007	0.0317547
10 weeks	2.1881	2.188	0.0006	0.0274211
16 weeks	2.0974	2.096	0.0014	0.0667493

TABLE 15: Changes in implant weights after the dynamic dissolution test.

In static dissolution assays, coated implants were immersed in 50 mL of plasma without agitation. pH values showed a slight increase during the first 48 hours but stabilized thereafter (Table 13). Cumulative mass loss remained below 0.1% after 16 weeks, confirming the stability of the coatings (Figure 58). Optical microscopy images demonstrated the preservation of surface morphology over the test duration (Figures 59,60). SEM images revealed progressive smoothing of the surface but no detachment or peeling (Figures 61,62). Elemental mapping by EDX confirmed the continued presence of calcium, phosphorus, and silver across the surface (Figures 63-65).

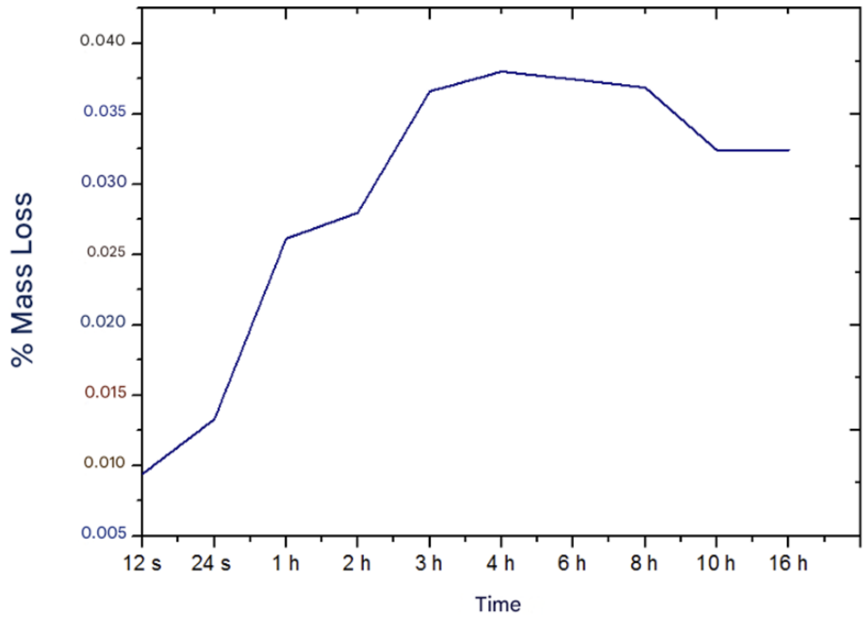

FIGURE 58: Curve of change in weight of coating as a result of static test.

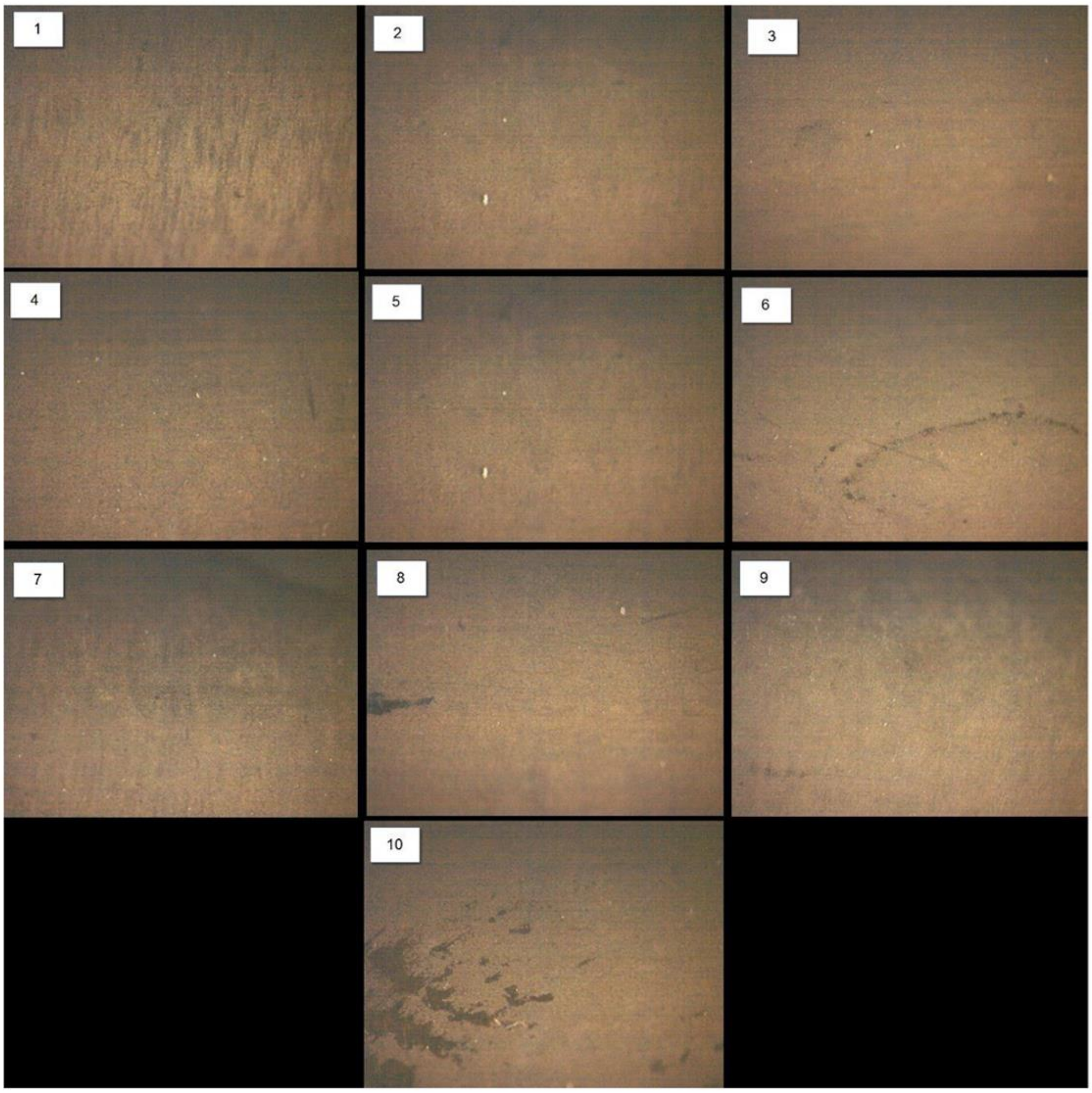

FIGURE 59: Optical surface images of coatings before static testing.

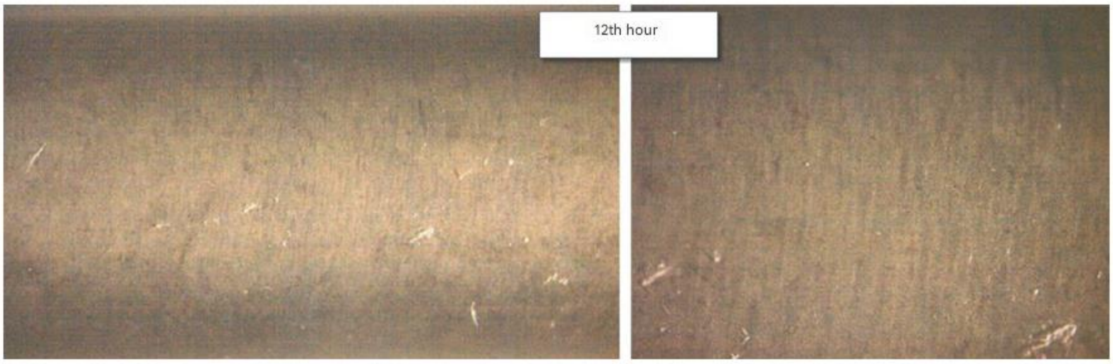
12th hour

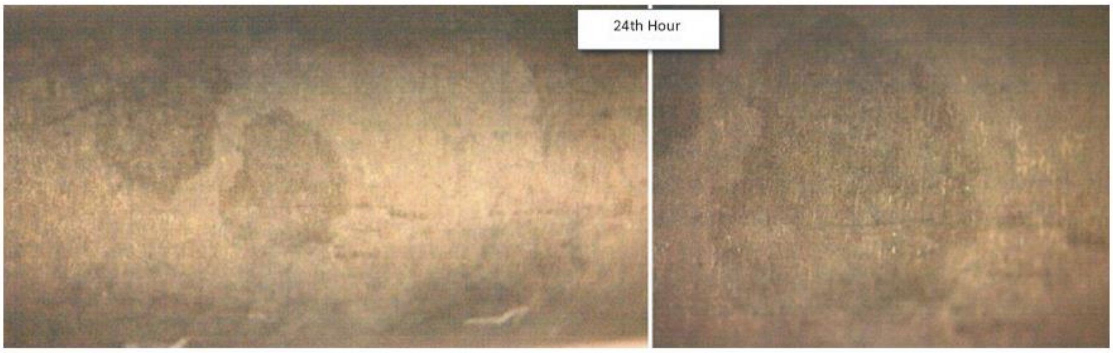
24th Hour

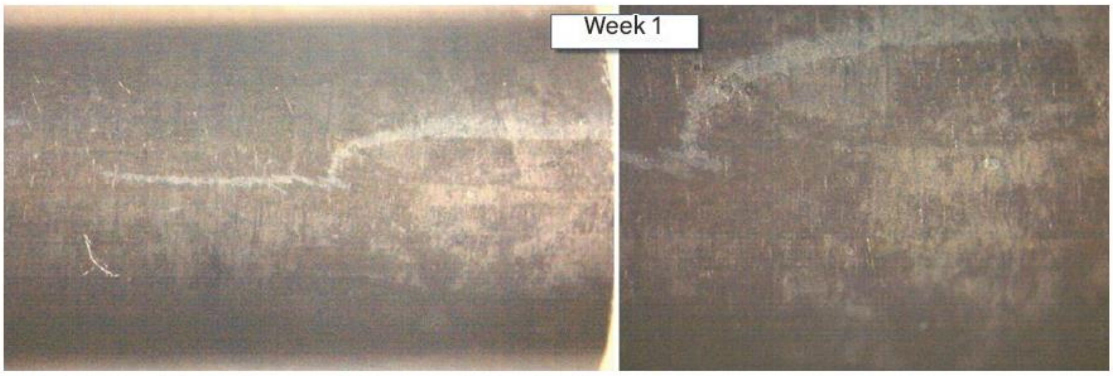
Week 1

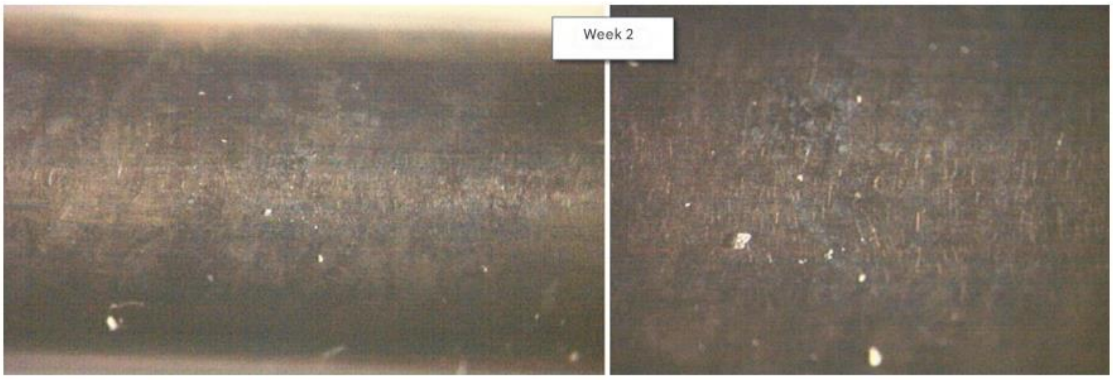
Week 2

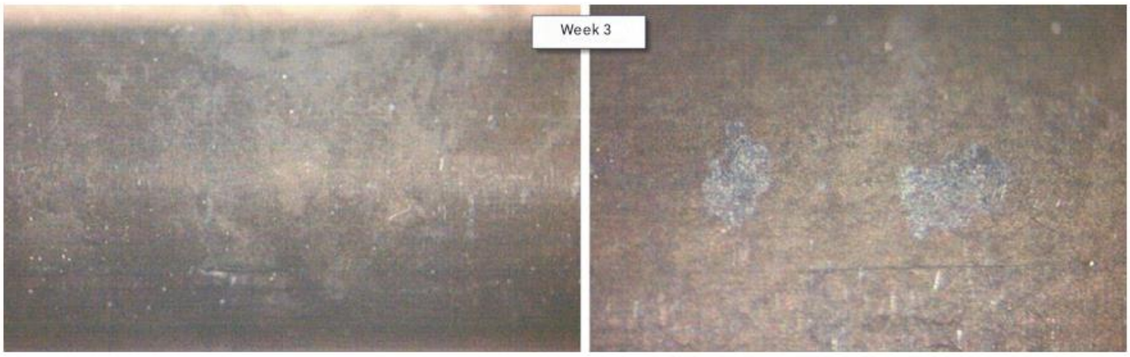
Week 3

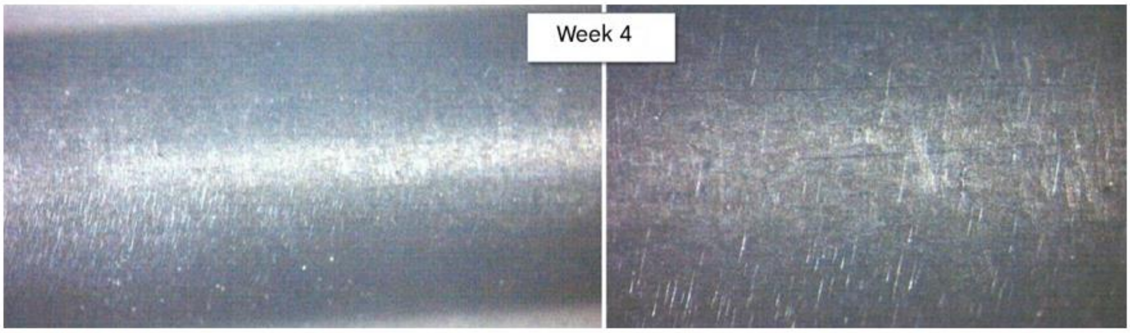
Week 4

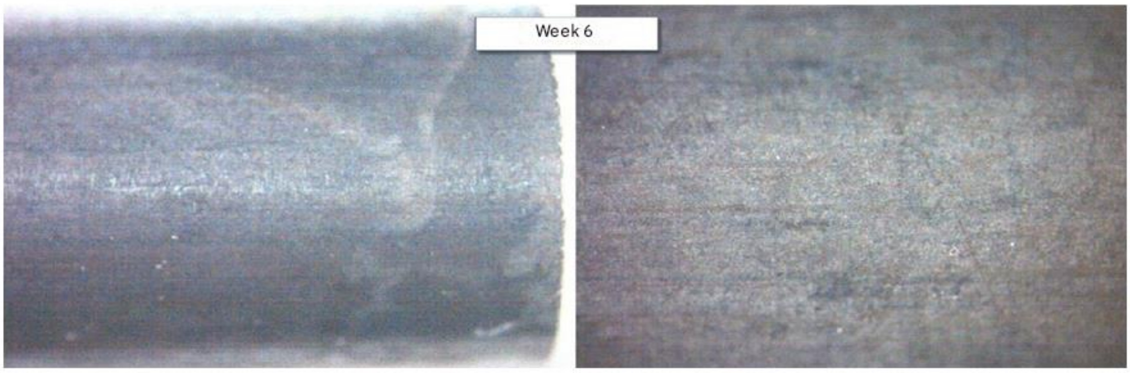
Week 6

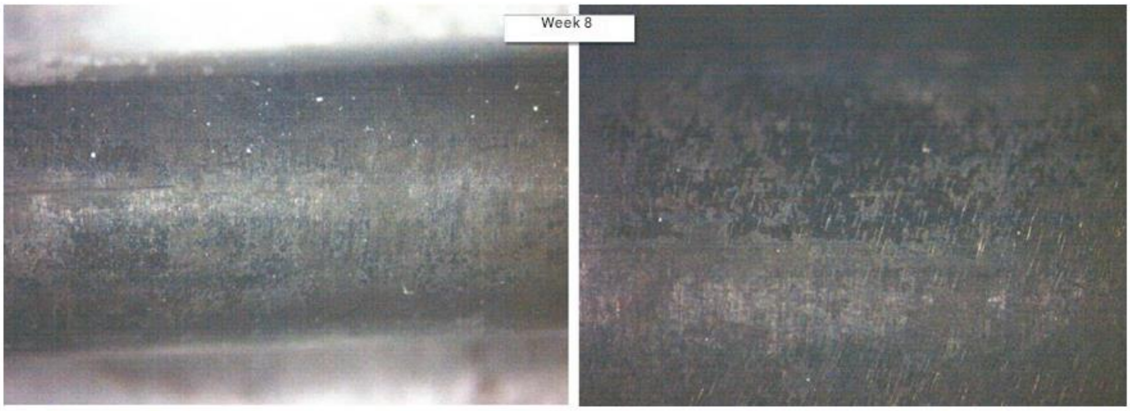
Week 8

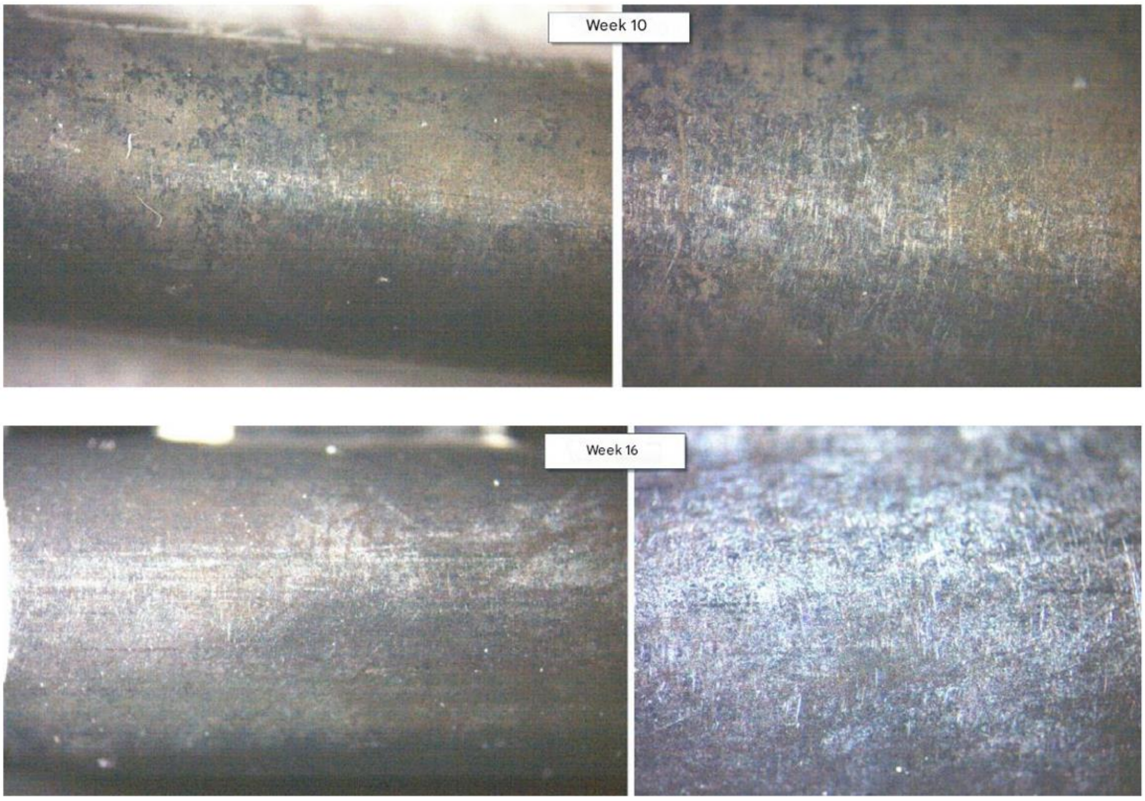

FIGURE 60: Optical images of surfaces statically held in plasma liquid for different periods of time.

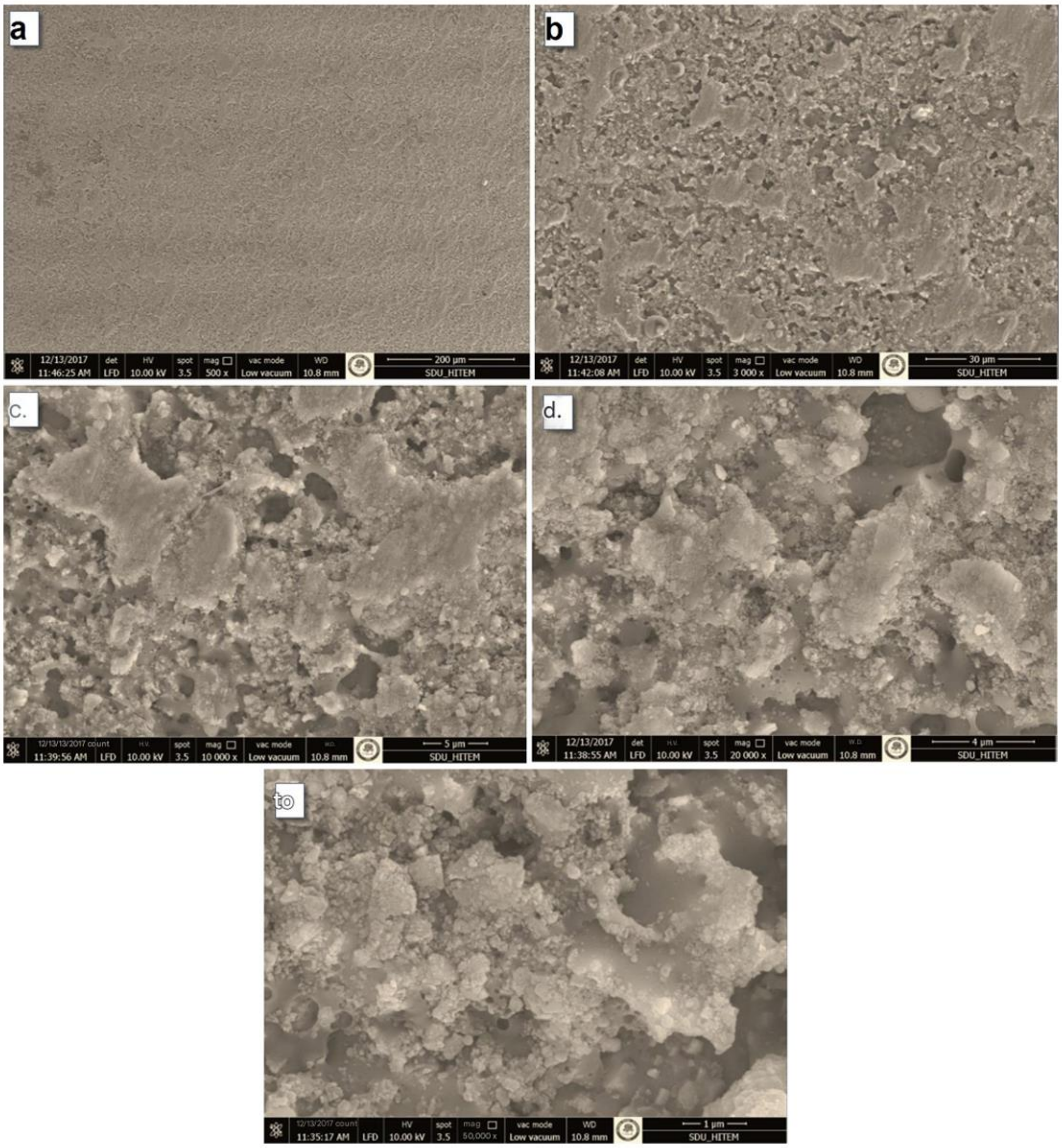

FIGURE 61: SEM images of the coating before the experiment a) 0.5 kX b) 3 kX c) 10 kX d) 20 kX e) 50 Kx.

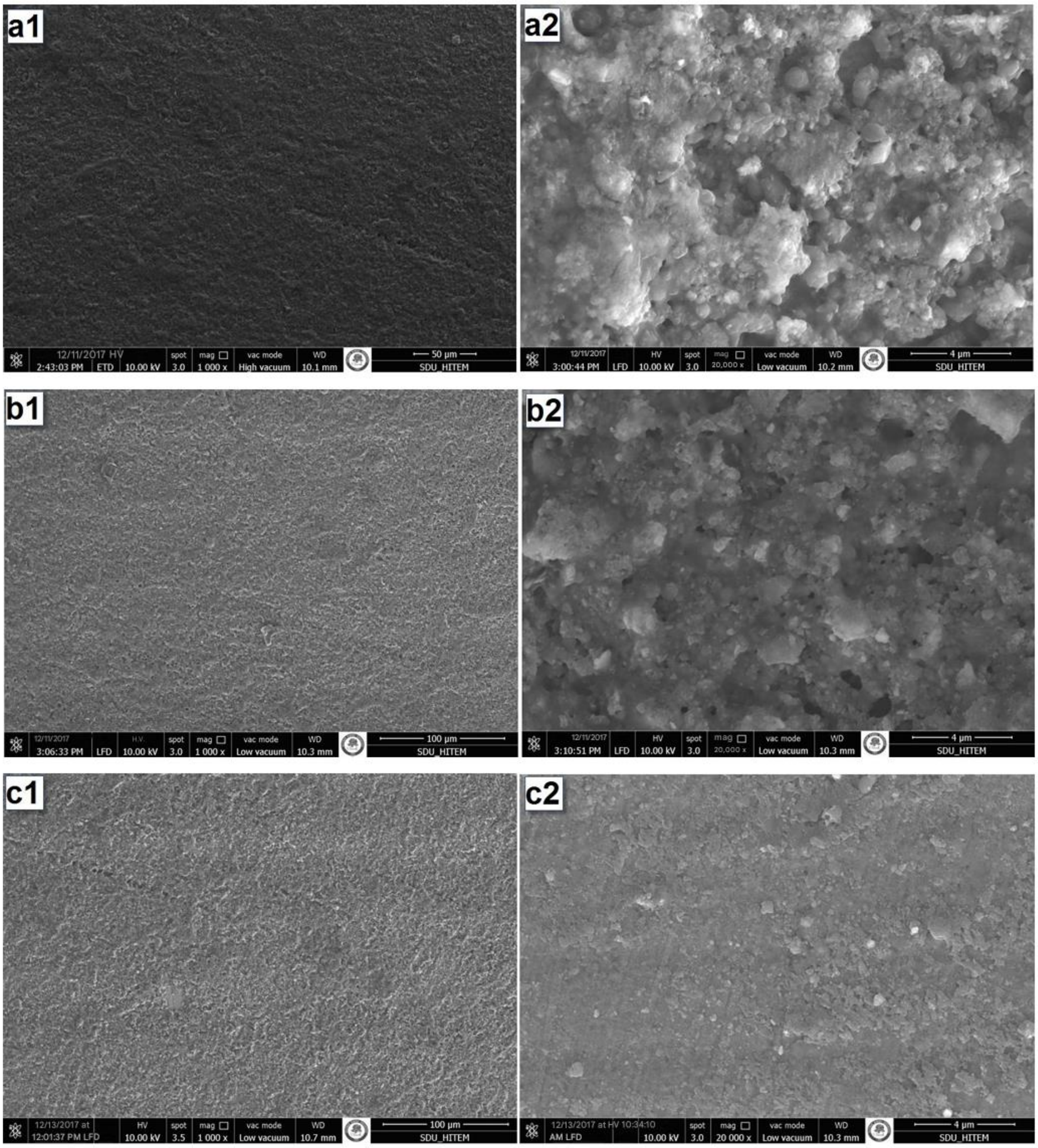
a1
50 µm
SDU_HITEM
a2
4 µm
SDU_HITEM
b1
100 µm
SDU_HITEM
b2
4 µm
SDU_HITEM
c1
100 µm
SDU_HITEM
c2
4 µm
SDU_HITEM

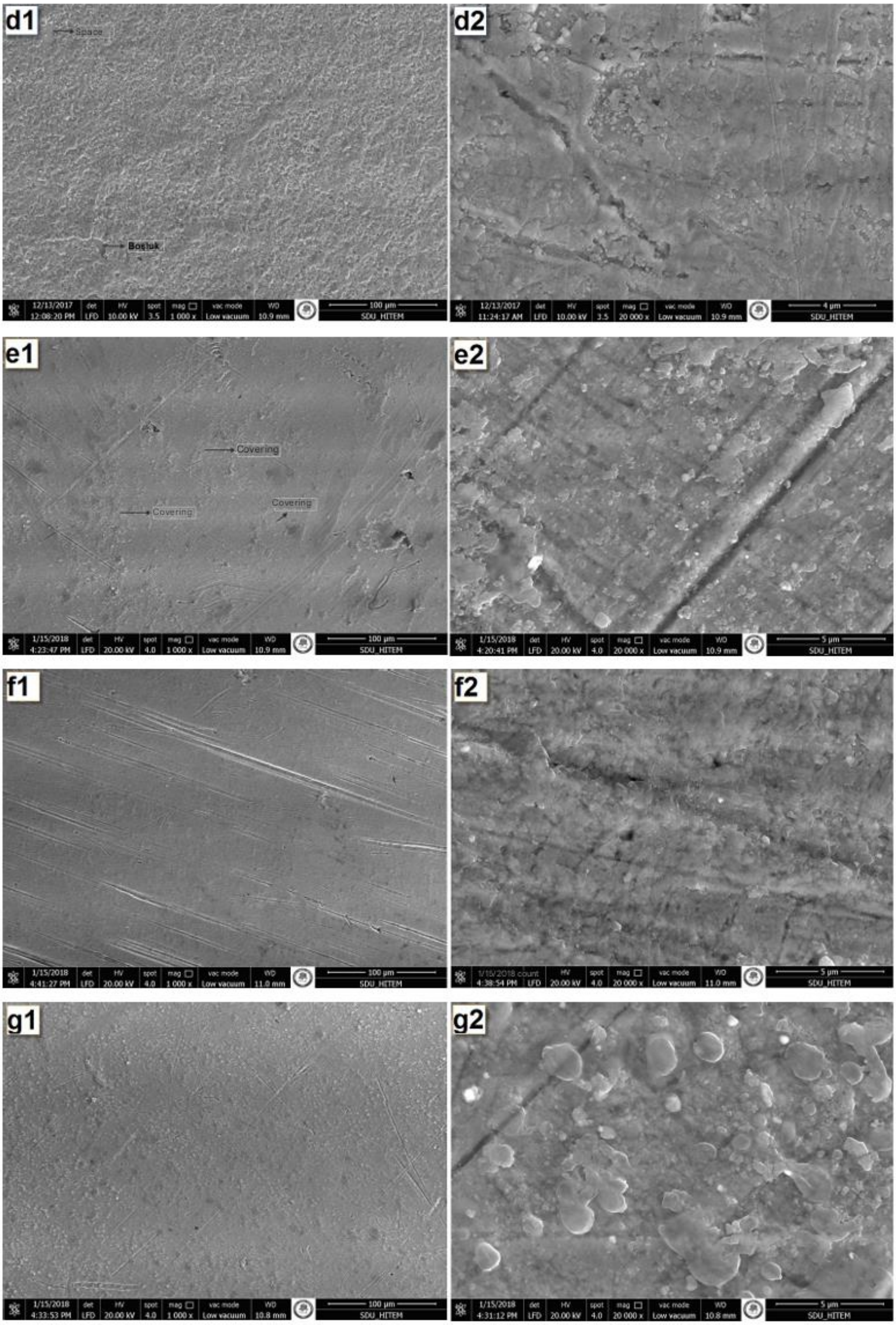
d1
Space
Boşluk
d2
e1
Covering
Covering
Covering
e2
f1
f2
g1
g2

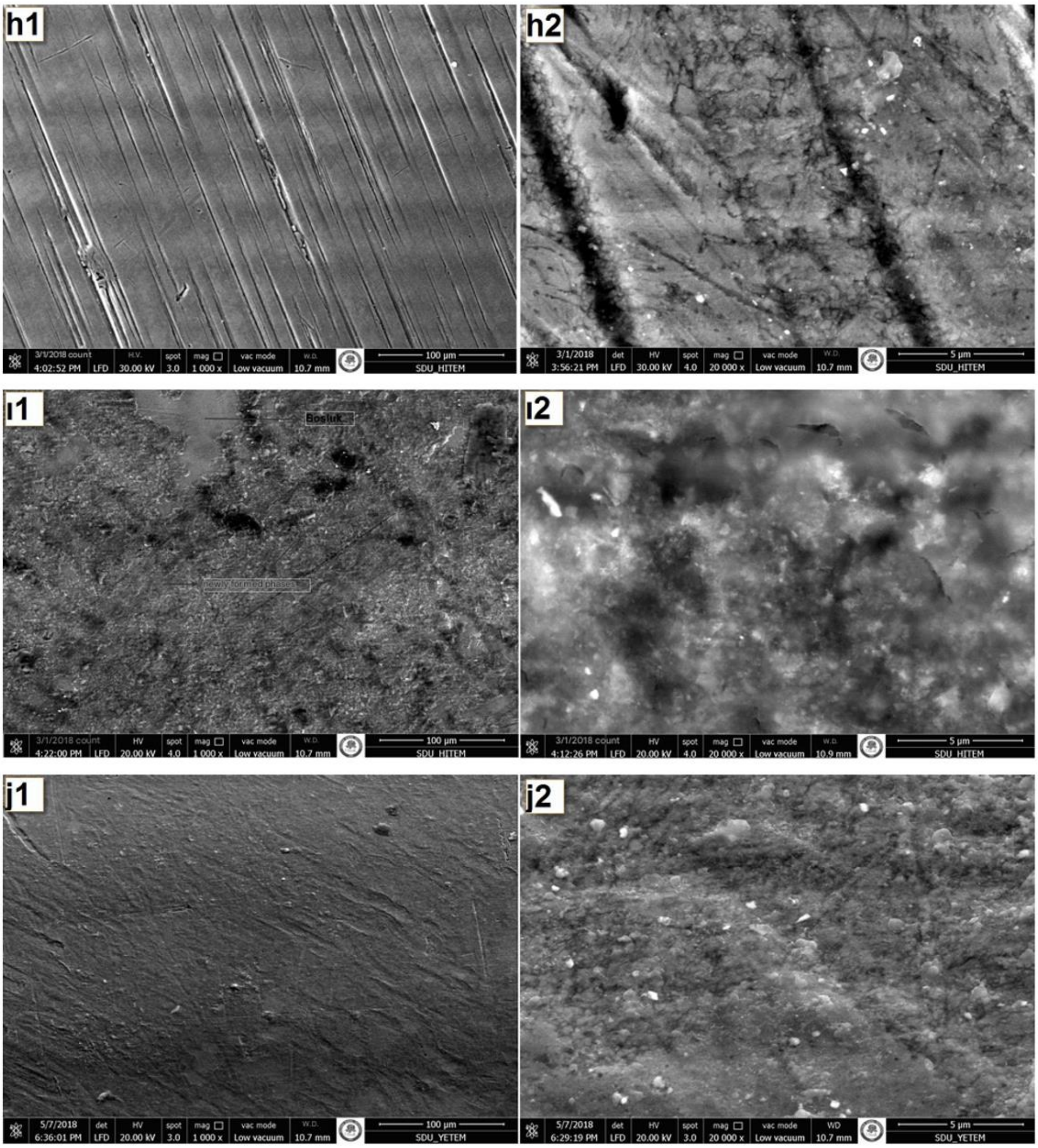

FIGURE 62: **Static conditions:**
a-1) 12 h, 1KX a-2) 12 h, 20KX b-1) 24 h, 1KX b-2) 24 h, 20KX
c-1) 1 week, 1KX c-2) 1 week, 20KX d-1) 2 weeks, 1KX d-2) 2 weeks, 20KX
e-1) 3 weeks, 1KX e-2) 3 weeks, 20KX f-1) 4 weeks, 1KX f-2) 4 weeks, 20KX
g-1) 6 weeks, 1KX g-2) 6 weeks, 20KX h-1) 8 weeks, 1KX h-2) 8 weeks, 20KX
i-1) 10 weeks, 1KX i-2) 10 weeks, 20KX j-1) 16 weeks, 1KX j-2) 16 weeks, 20KX*Microstructures of the coatings kept at different time intervals.*

.

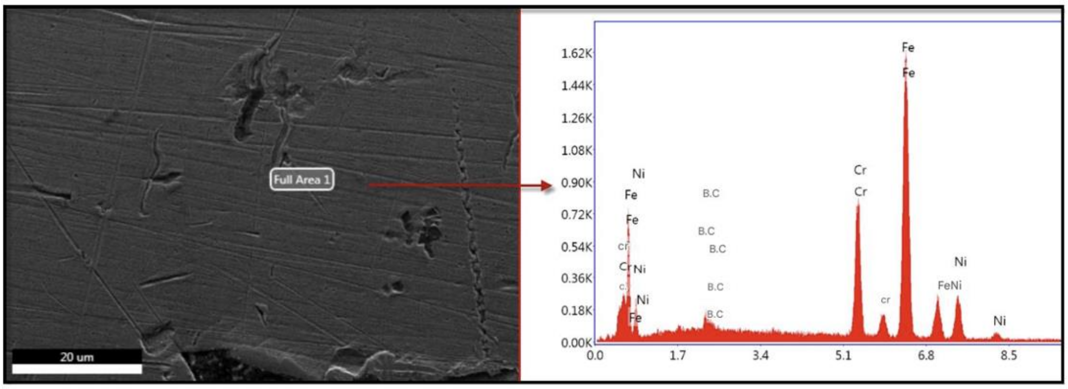

(a)

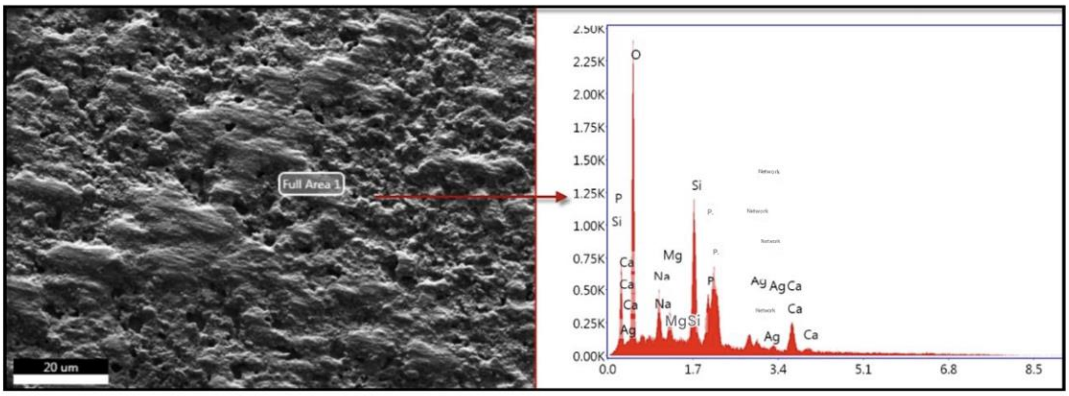

(b)

FIGURE 63: a) Chemical analysis of the metal implant and b) the coated surface

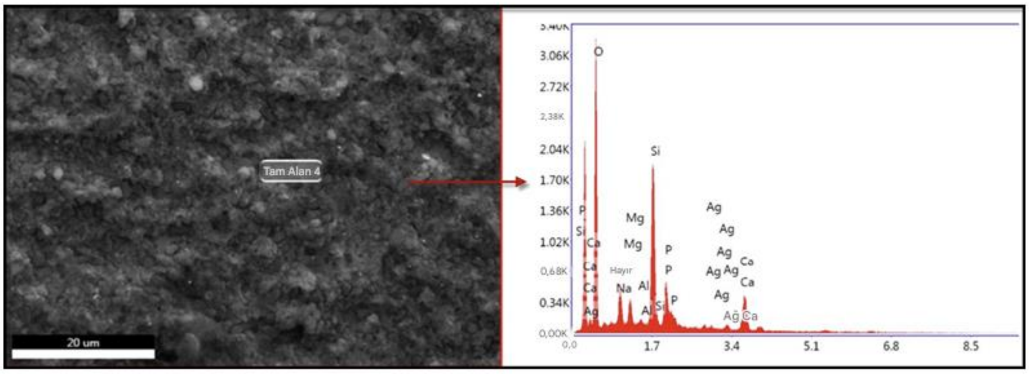
Tam Alan 4
20 um
O
Si
Mg
P
Na
Al
Ca
Ag
Hayır
3.06K
2.72K
2,38K
2.04K
1.70K
1.36K
1.02K
0,68K
0.34K
0,00K
0,0
1.7
3.4
5.1
6.8
8.5

(A)

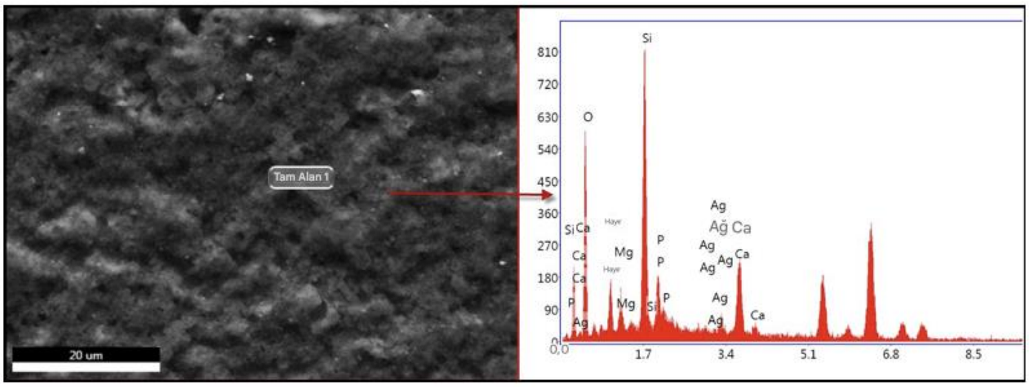
Tam Alan 1
20 um
Si
O
Mg
P
Ca
Ag
Ağ Ca
Hayır
810
720
630
540
450
360
270
180
90
0
0,0
1.7
3.4
5.1
6.8
8.5

(B)

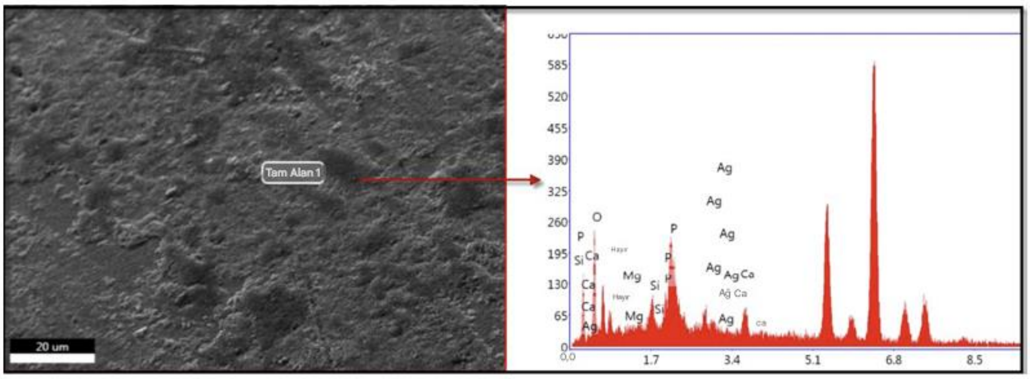
Tam Alan 1
20 um
O
P
Si
Ca
Mg
Ag
Ağ Ca
Hayır
585
520
455
390
325
260
195
130
65
0
0,0
1.7
3.4
5.1
6.8
8.5

(C)

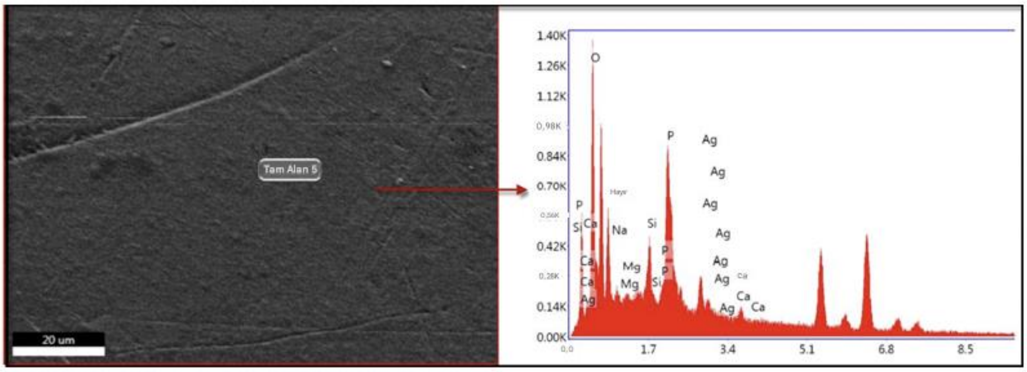
Tam Alan 5
20 um
O
P
Si
Ca
Na
Mg
Ag
Hayır
1.40K
1.26K
1.12K
0,98K
0.84K
0.70K
0.42K
0.14K
0.00K
0,0
1.7
3.4
5.1
6.8
8.5

(D)

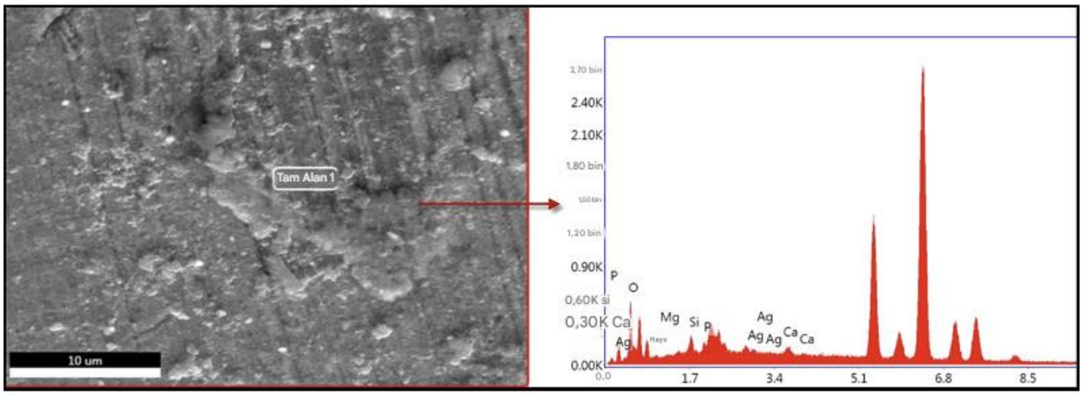

Tam Alan 1
10 um
2,70 bin
2.40K
2.10K
1,80 bin
1,20 bin
0.90K
0,60K
0,30K
0.00K
P
O
Si
Ca
Mg
Si
P
Ag
Ag
Ag
Ca
Ca
0,0
1.7
3.4
5.1
6.8
8.5

(e)

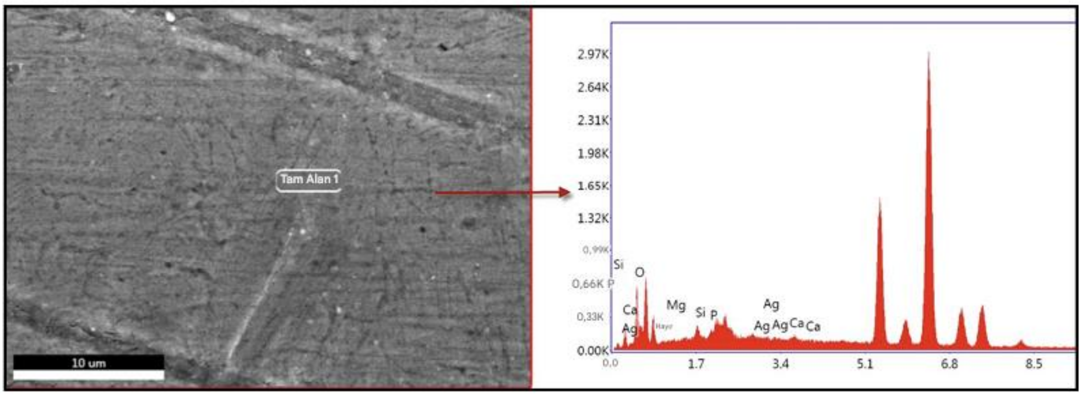

Tam Alan 1
10 um
2.97K
2.64K
2.31K
1.98K
1.65K
1.32K
0,99K
0,66K
0,33K
0.00K
Si
O
P
Ca
Ag
Mg
Si
P
Ag
Ag
Ag
Ca
Ca
0,0
1.7
3.4
5.1
6.8
8.5

(F)

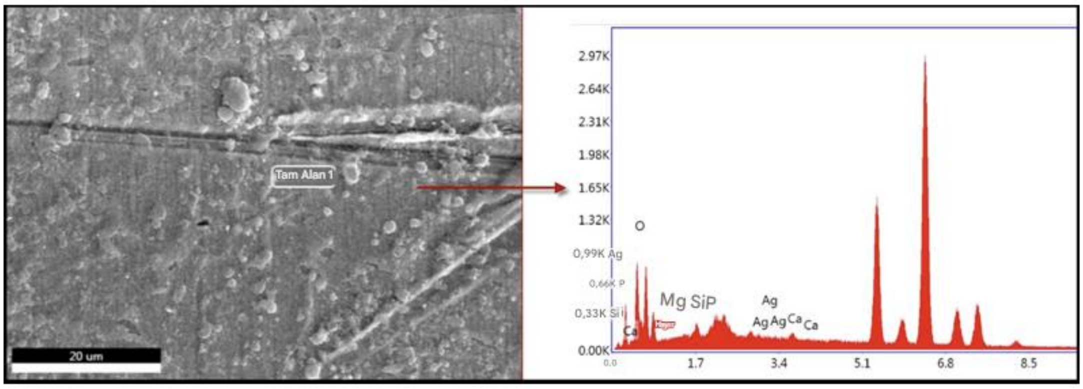

Tam Alan 1
20 um
2.97K
2.64K
2.31K
1.98K
1.65K
1.32K
0,99K
0,66K
0,33K
0.00K
O
Ag
P
Si
Ca
Mg
SiP
Ag
Ag
Ag
Ca
Ca
0,0
1.7
3.4
5.1
6.8
8.5

(G)

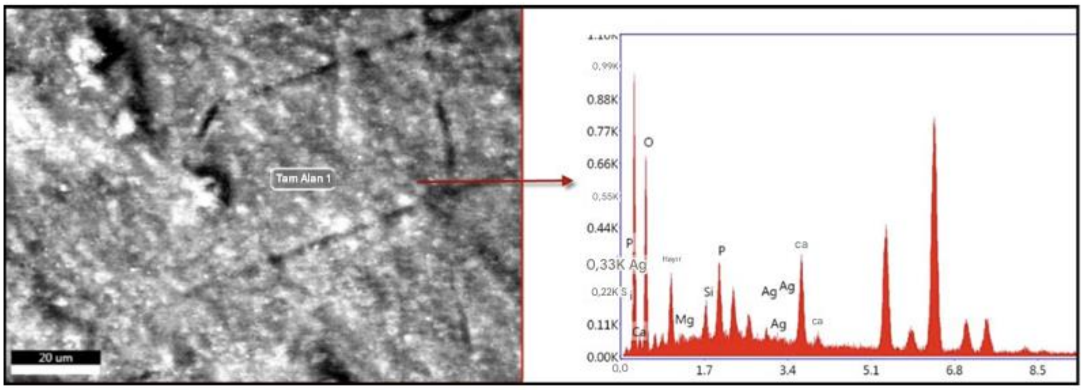

Tam Alan 1
20 um
0,99K
0.88K
0.77K
0.66K
0,55K
0.44K
0,33K
0,22K
0.11K
0.00K
O
P
Ag
S
Ca
Mg
Si
P
Ag
Ag
Ag
ca
ca
0,0
1.7
3.4
5.1
6.8
8.5

(H)

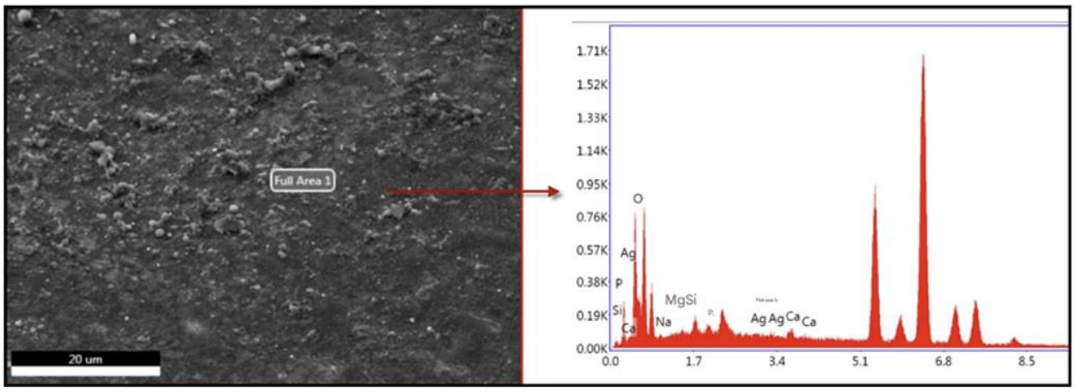

(ı)

FIGURE 64: Chemical analysis results of metal implants kept statically in plasma fluid for different time periods: a) 12 hours, b) 24 hours, c) 1 week, d) 2 weeks, e) 3 weeks, f) 4 weeks, g) 6 weeks, h) 10 weeks, i) 16 weeks

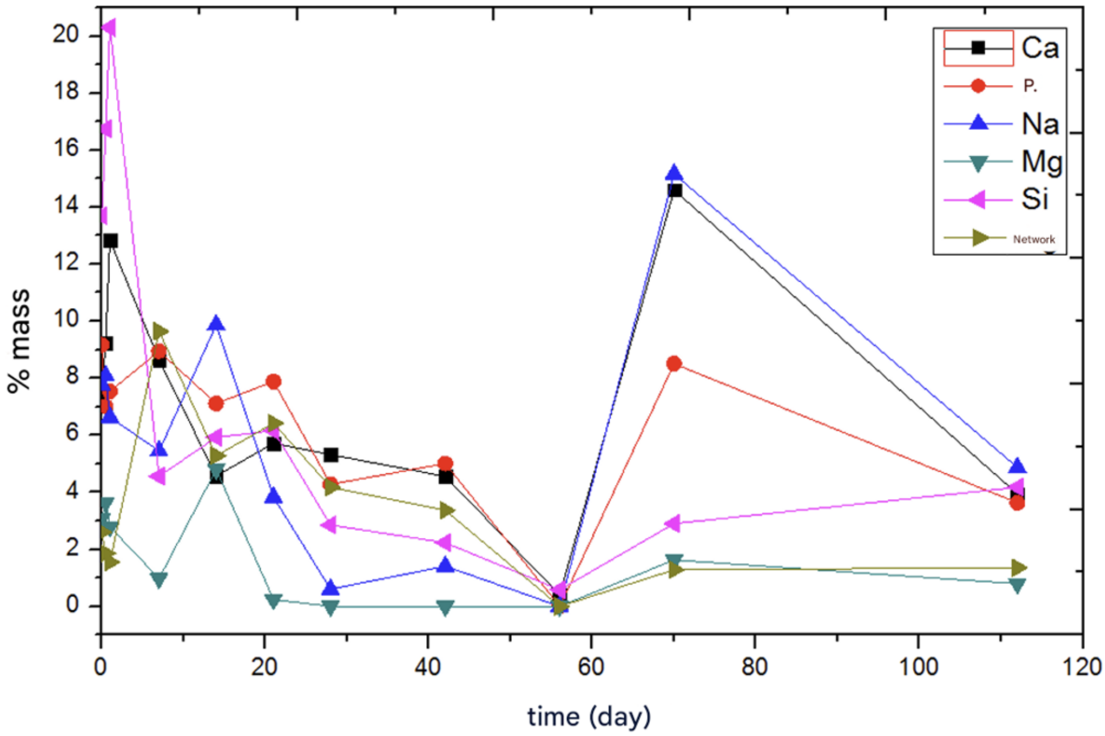

FIGURE 65: Changes in the elements on the metal implant depending on time after the static test.

Dynamic dissolution assays were conducted using a peristaltic pump that maintained continuous plasma flow at ~5 mL/min. This setup produced slightly higher ion release compared with static conditions, but structural integrity was maintained. pH values remained stable in the physiological range of 7.3–7.5 (Table 14). Mass loss again remained below 0.1% over 16 weeks (Table 15). Optical images indicated a polishing effect on the surface due to plasma circulation (Figures 66,67). SEM images confirmed that the coating microstructure was

preserved without delamination (Figure 68). EDX analyses further revealed sustained silver release with Ca and P continuing to be detectable at the surface (Figures 69,70).

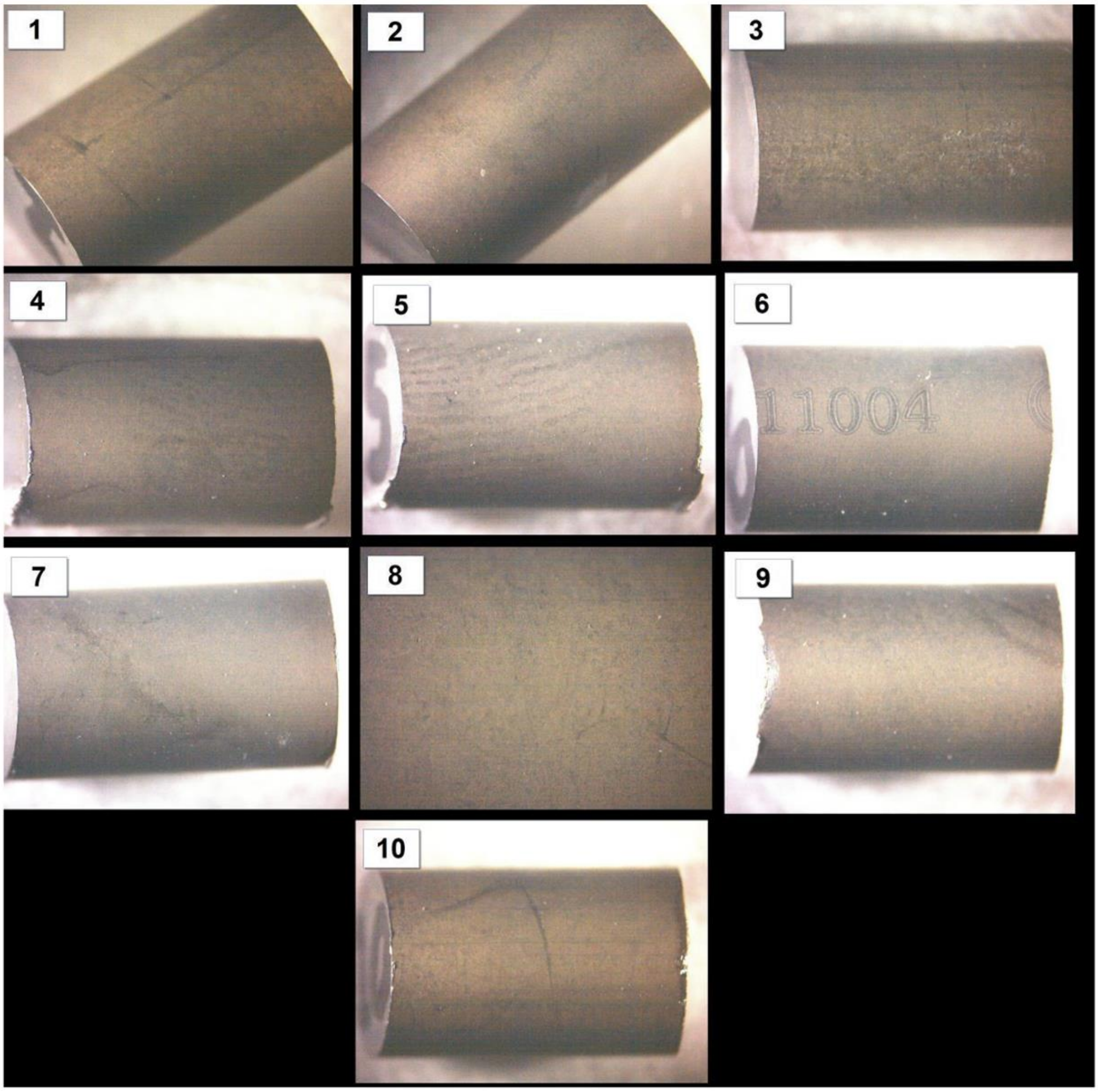

FIGURE 66: Optical surface images of coatings before dynamic testing.

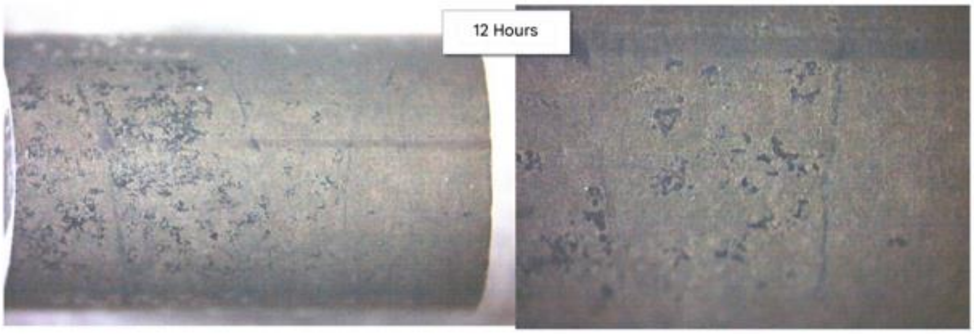
12 Hours

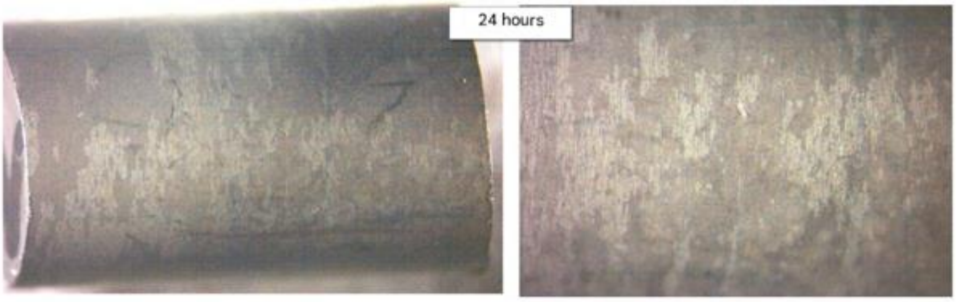
24 hours

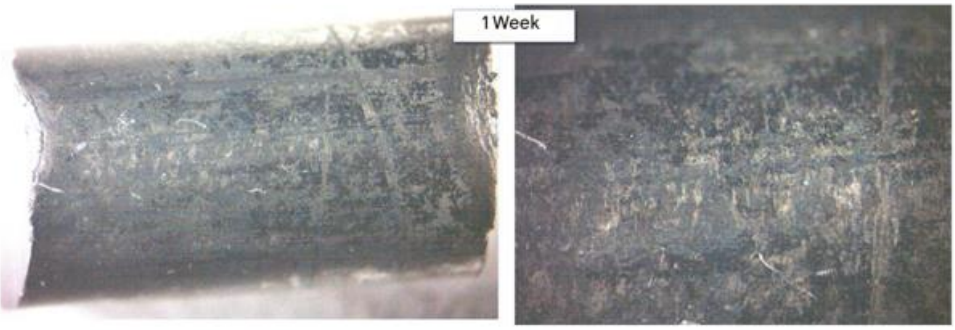
1 Week

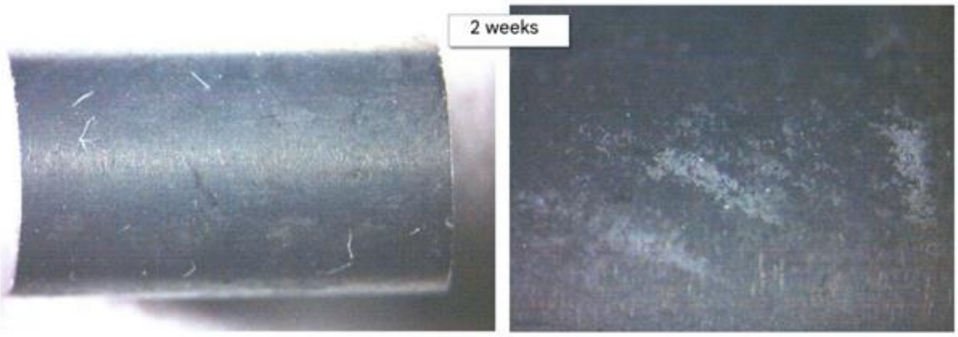
2 weeks

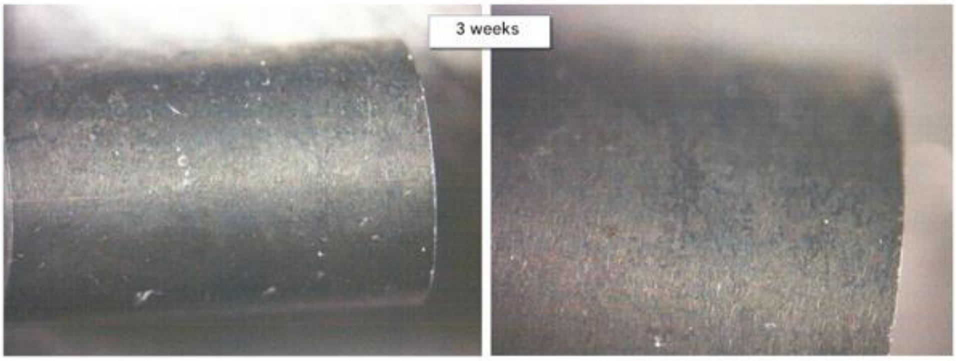
3 weeks

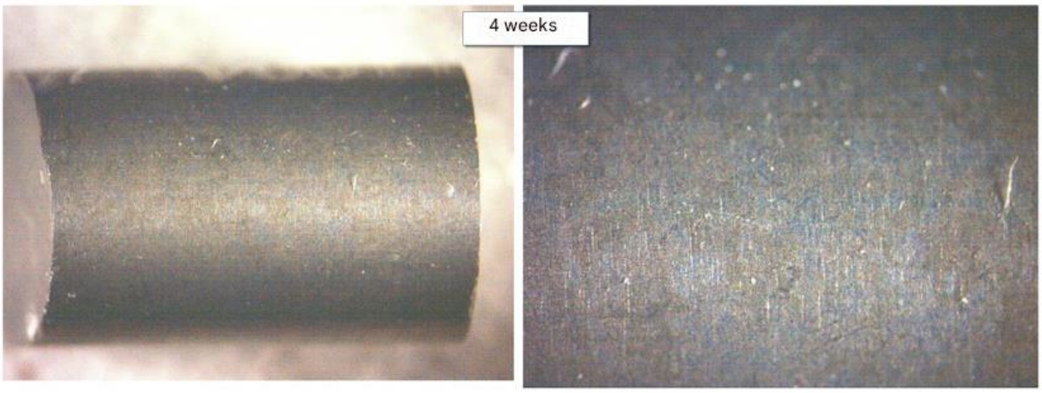
4 weeks

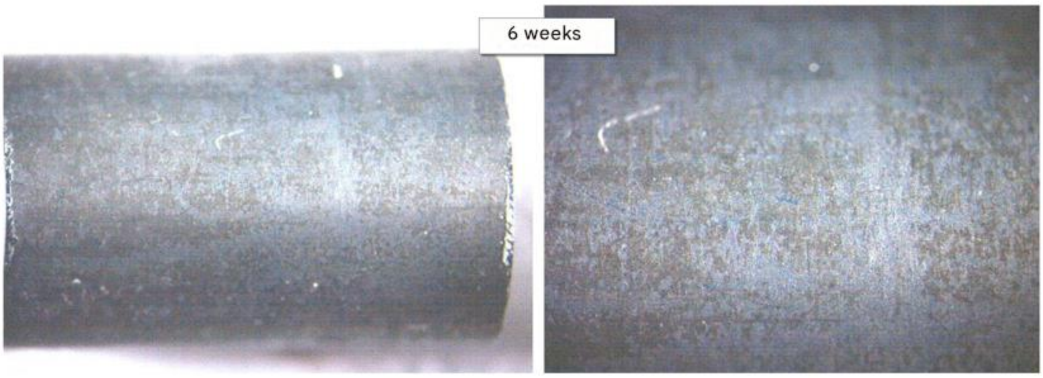
6 weeks

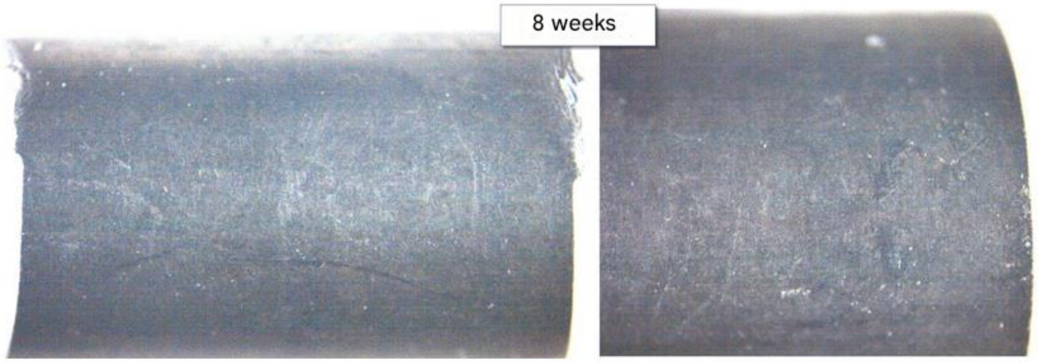
8 weeks

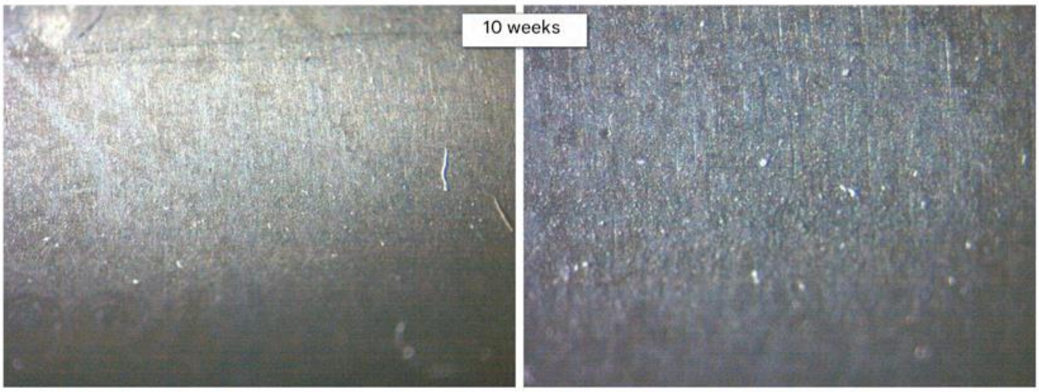
10 weeks

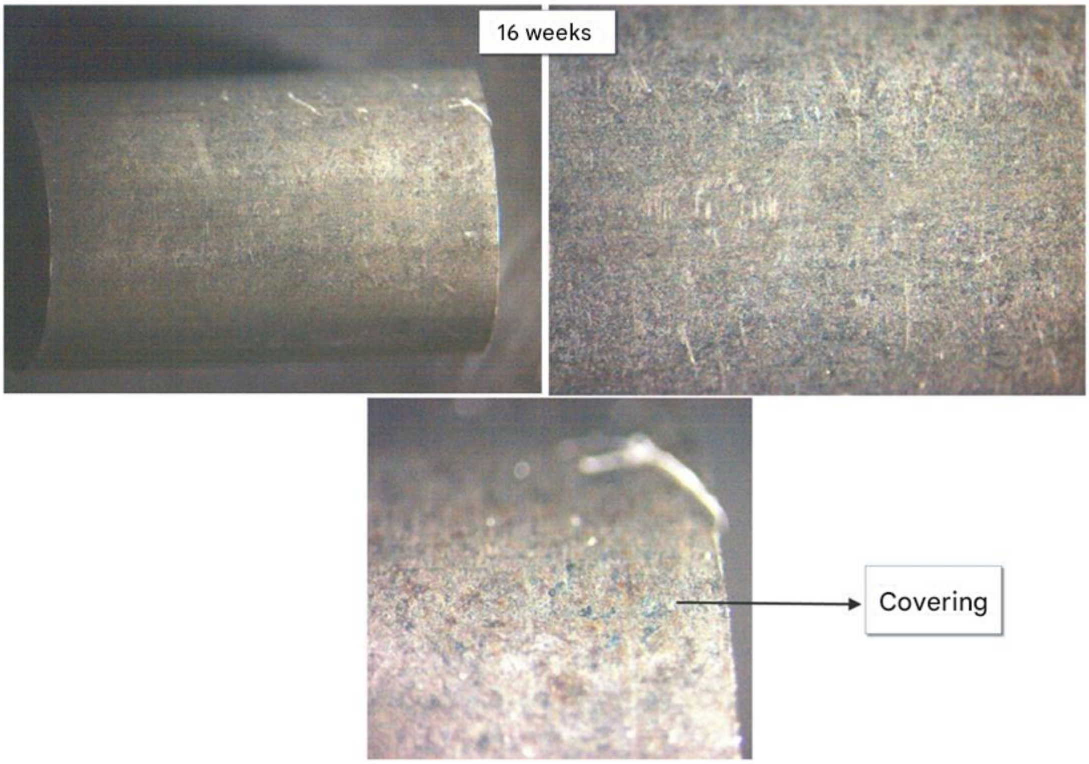

FIGURE 67: Optical images of surfaces dynamically immersed in plasma liquid for different periods of time.

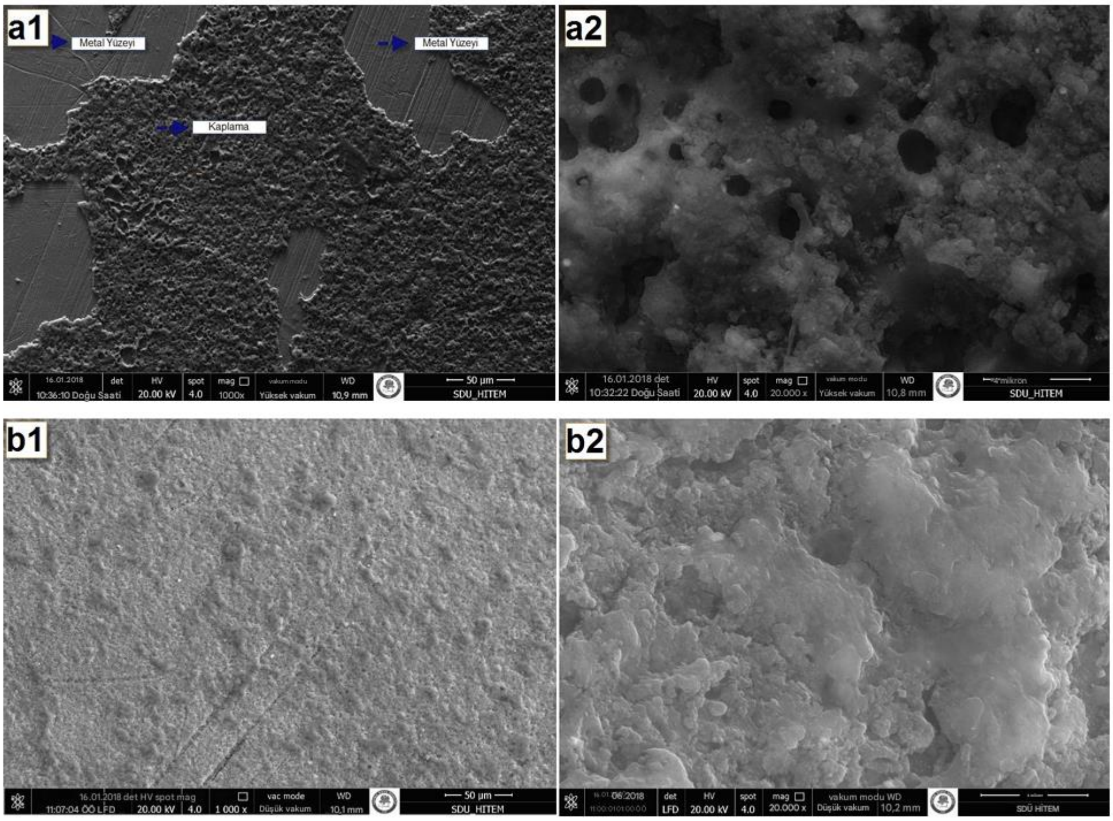
a1
Metal Yüzeyi
Metal Yüzeyi
Kaplama
50 µm
SDU_HITEM
a2
SDU_HITEM
b1
50 µm
SDU_HITEM
b2
SDÜ HİTEM

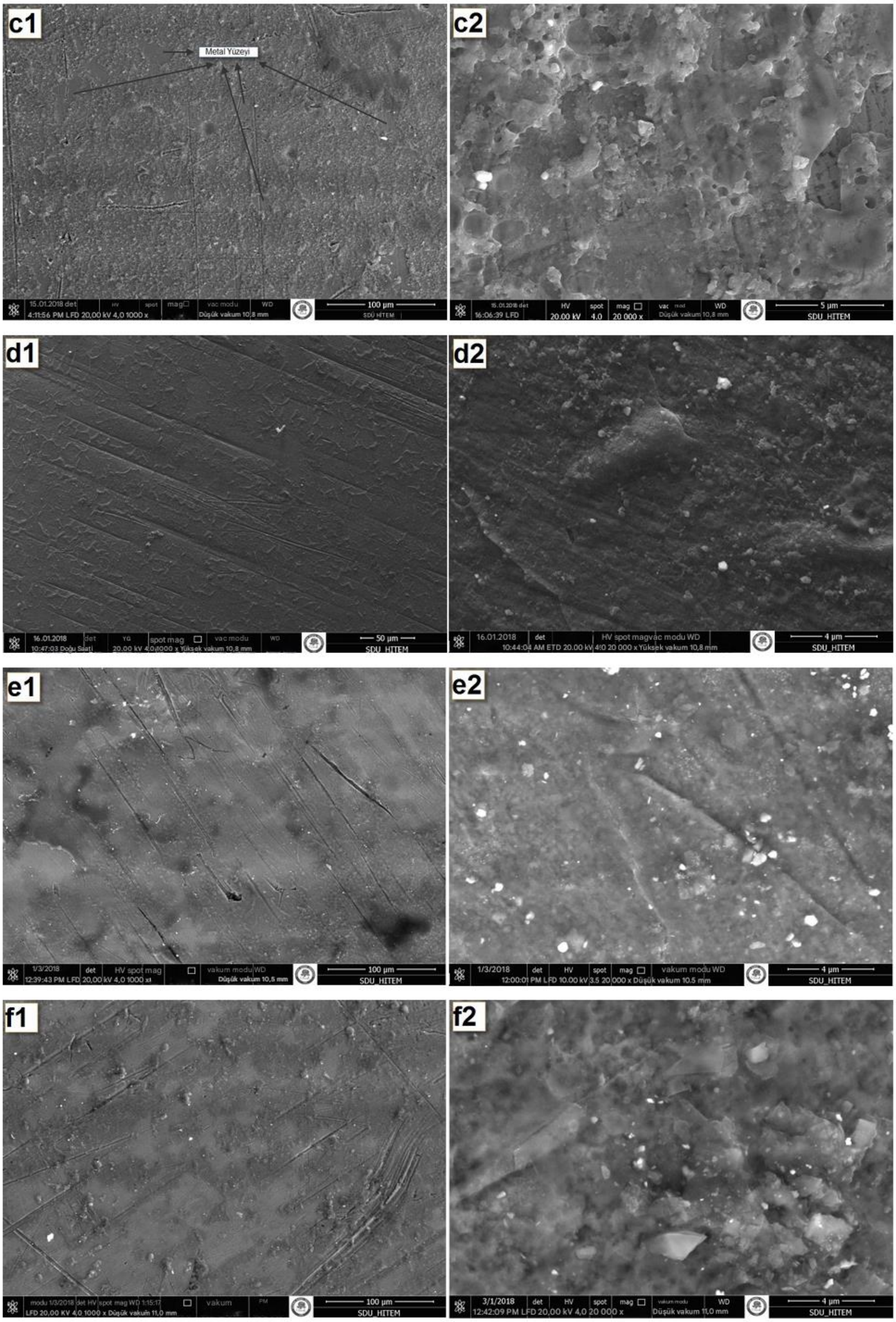
c1
Metal Yüzeyi
100 µm
c2
5 µm
SDU_HITEM
d1
50 µm
SDU_HITEM
d2
4 µm
SDU_HITEM
e1
100 µm
SDU_HITEM
e2
4 µm
SDU_HITEM
f1
100 µm
SDU_HITEM
f2
4 µm
SDU_HITEM

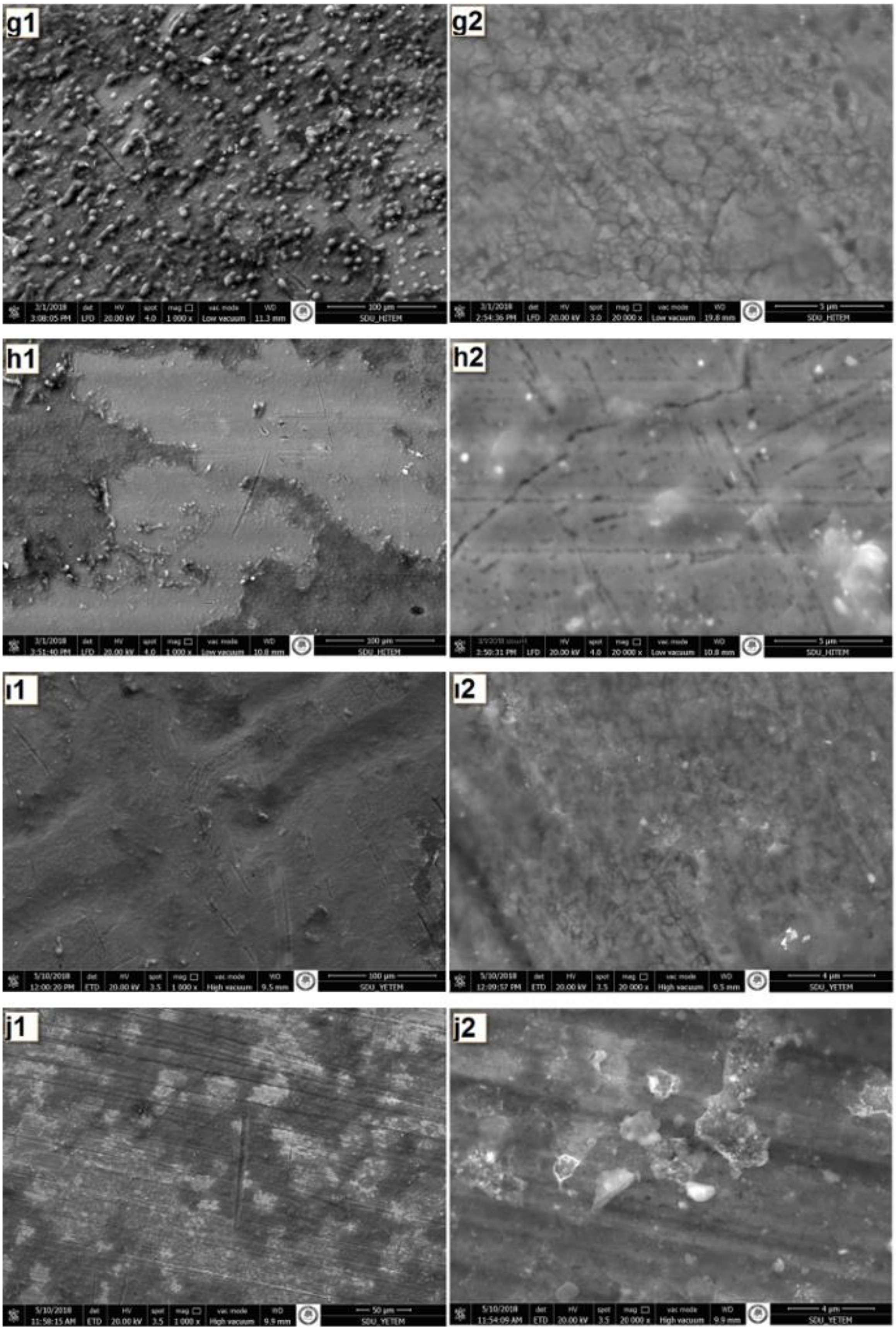
g1
g2
h1
h2
ı1
ı2
j1
j2

FIGURE 68: **Dynamic conditions:**
a-1) 12 h, 1KX a-2) 12 h, 20KX b-1) 24 h, 1KX b-2) 24 h, 20KX
c-1) 1 week, 1KX c-2) 1 week, 20KX d-1) 2 weeks, 1KX d-2) 2 weeks, 20KX
e-1) 3 weeks, 1KX e-2) 3 weeks, 20KX f-1) 4 weeks, 1KX f-2) 4 weeks, 20KX
g-1) 6 weeks, 1KX g-2) 6 weeks, 20KX h-1) 8 weeks, 1KX h-2) 8 weeks, 20KX
i-1) 10 weeks, 1KX i-2) 10 weeks, 20KX j-1) 16 weeks, 1KX j-2) 16 weeks, 20KX*Microstructures of the coatings kept at different time intervals.*

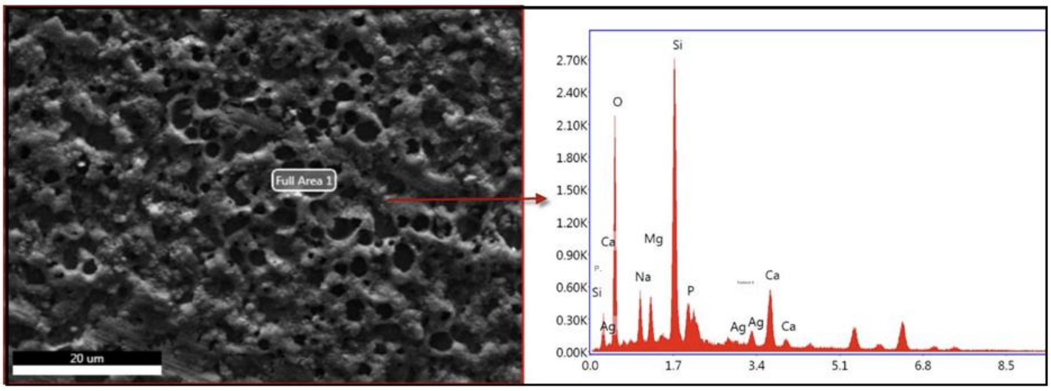

(a)

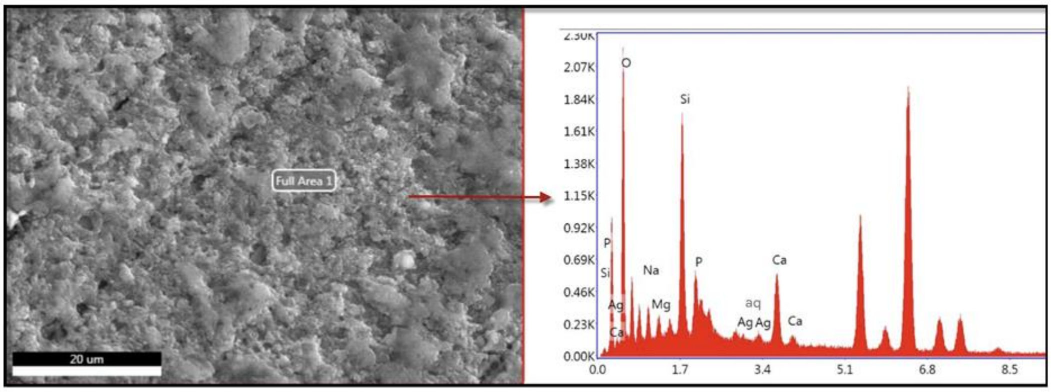

(b)

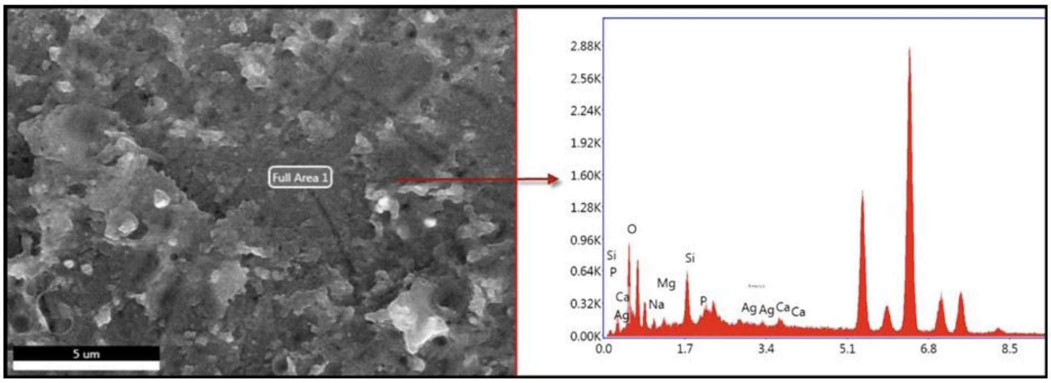

(C)

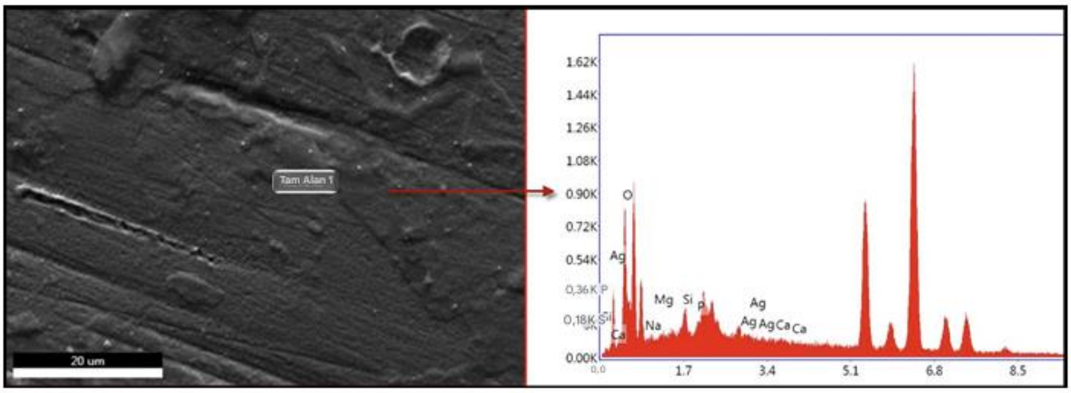
Tam Alan 1
20 um
1.62K
1.44K
1.26K
1.08K
0.90K
0.72K
0.54K
0,36K
0,18K
0.00K
O
Ag
P
Si
Ca
Mg
Si
P
Na
Ag
Ag Ag Ca Ca
0,0
1.7
3.4
5.1
6.8
8.5

(D)

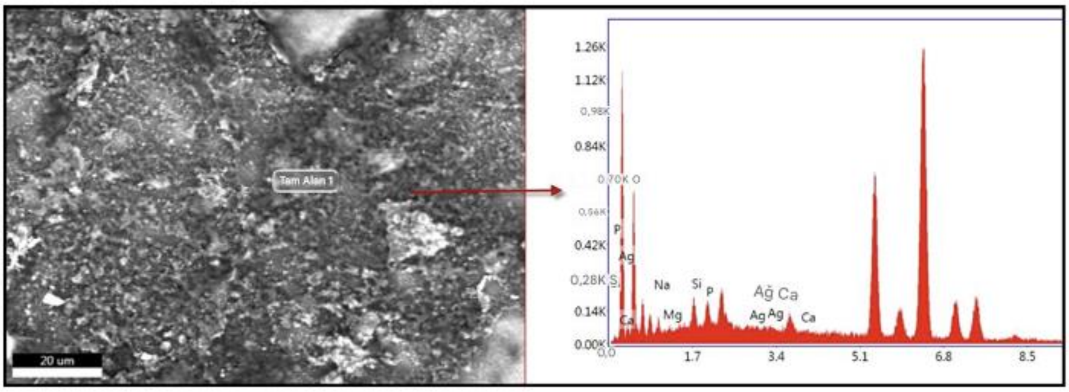
Tam Alan 1
20 um
1.26K
1.12K
0,98K
0.84K
0,70K O
0,56K
0.42K
0,28K
0.14K
0.00K
P
Ag
Na
Si
P
Mg
Ca
Ağ Ca
Ag Ag
Ca
0,0
1.7
3.4
5.1
6.8
8.5

(e)

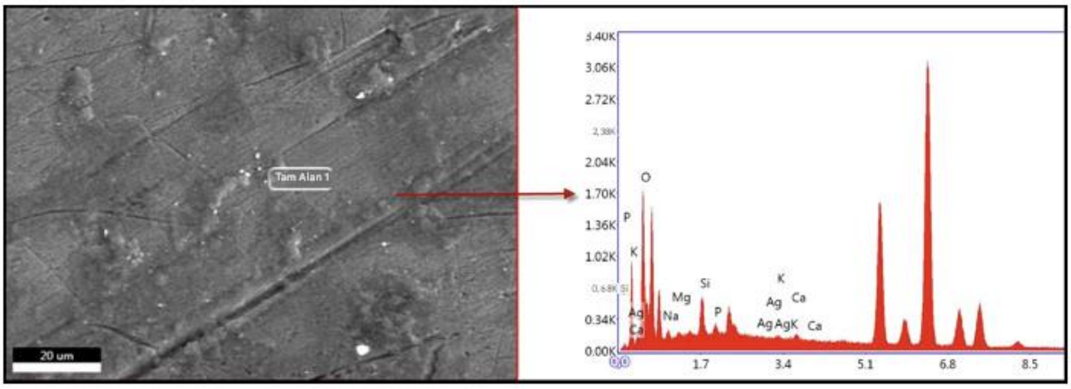
Tam Alan 1
20 um
3.40K
3.06K
2.72K
2,38K
2.04K
1.70K
1.36K
1.02K
0,68K
0.34K
0.00K
O
P
K
Si
Mg
Si
Ag
Na
P
Ca
K
Ag Ca
Ag Ag K Ca
0,0
1.7
3.4
5.1
6.8
8.5

(F)

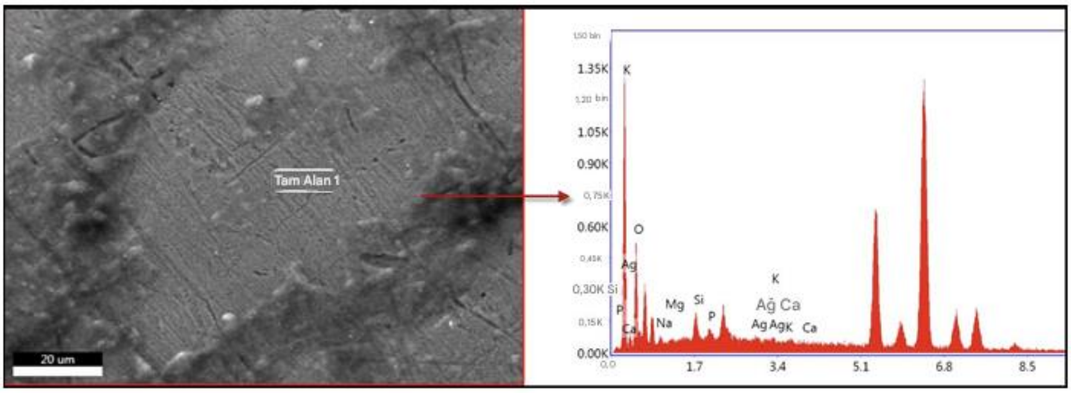
Tam Alan 1
20 um
1,50 bin
1.35K
1,20 bin
1.05K
0.90K
0,75K
0.60K
0,45K
0,30K
0,15K
0.00K
K
O
Ag
Si
Mg
Si
P
P
Na
Ca
K
Ağ Ca
Ag Ag K Ca
0,0
1.7
3.4
5.1
6.8
8.5

(g)

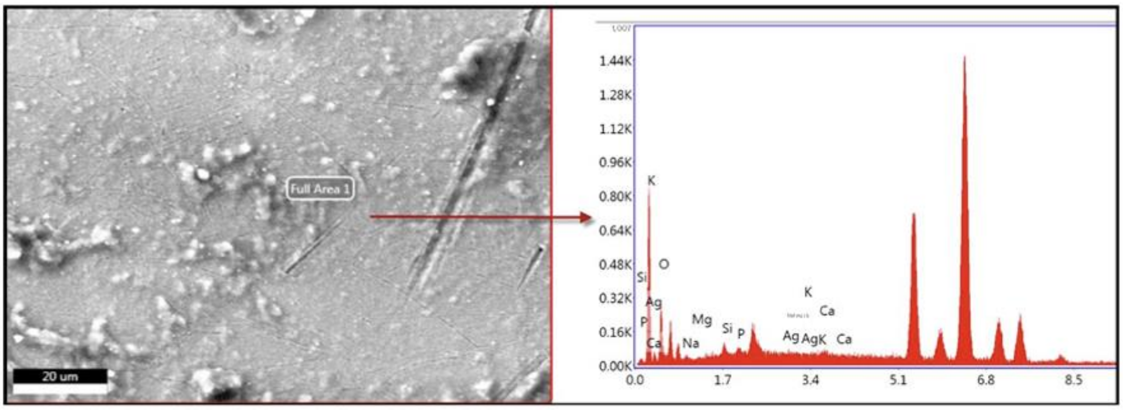

(h)

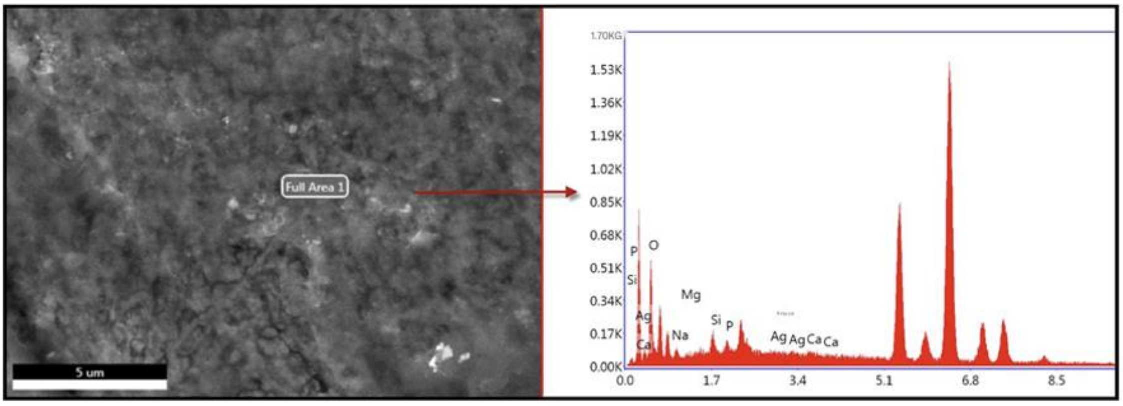

(ı)

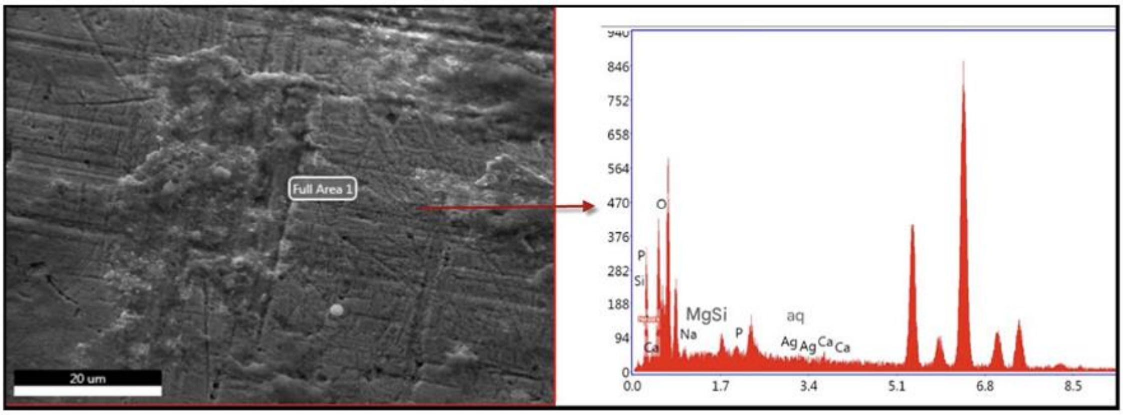

(j)

FIGURE 69: Chemical analysis results of metal implants dynamically kept in plasma liquid for different time periods: a) 12 hours, b) 24 hours, c) 1 week, d) 2 weeks, e) 3 weeks, f) 4 weeks, g) 6 weeks, h) 8 weeks, i) 10 weeks, j) 16 weeks

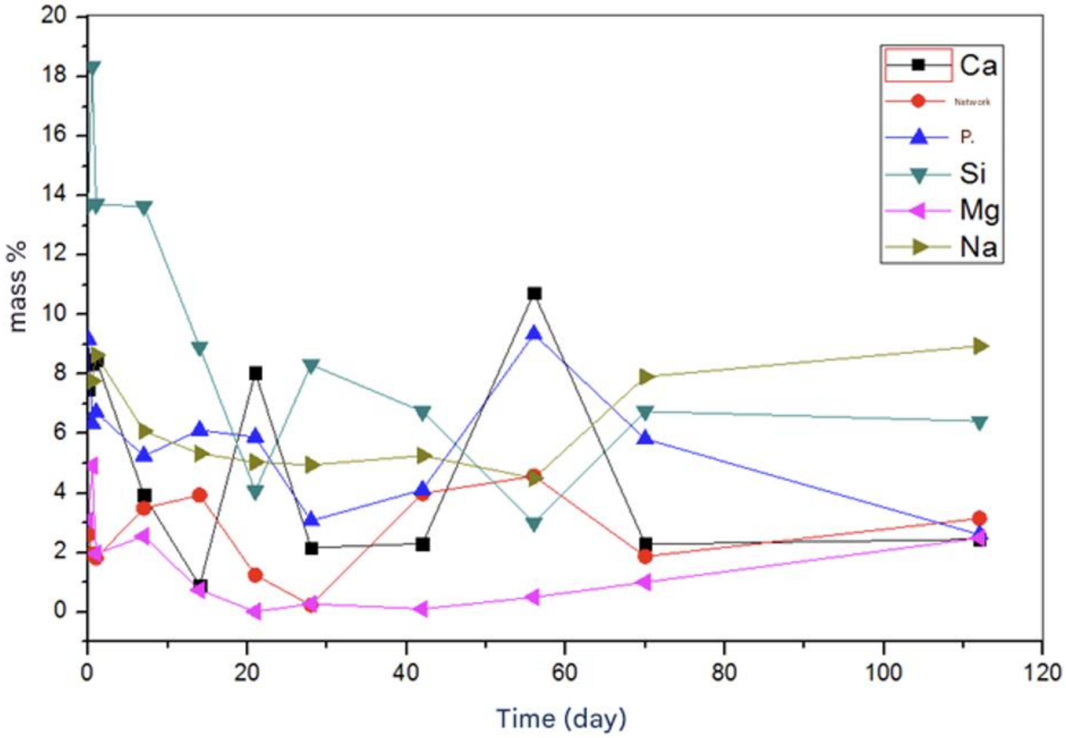

FIGURE 70: Time-dependent change of elements on the metal implant after dynamic testing

In addition to surface analyses, the plasma itself was examined by ICP and EDX to quantify released elements. The results showed gradual, controlled release of calcium, phosphorus, and silver ions (Figure 71; Table 16-18). The release profile was consistent with long-term antibacterial activity, without exhibiting a burst release effect. Importantly, ion concentrations remained below reported cytotoxic limits, demonstrating that the coatings were biocompatible even under extended exposure conditions.

Time	O	Na	Mg	Si	P	Ag	Ca	Mo	Cr	Fe	Ni
Substrate								2.11	18.75	65.22	13.92
0 h	56.19	7.77	3.09	13.70	9.17	2.60	7.48				
12 h	52.23	8.08	3.64	16.74	7.01	1.86	9.22				
24 h	48.38	6.63	2.79	20.30	7.54	1.55	12.83				
1 week	1.78	5.45	0.98	4.56	8.95	9.64	8.64				
2 weeks	62.44	9.87	4.80	5.93	7.11	5.28	4.57				
3 weeks	69.74	3.83	0.24	6.16	7.88	6.42	5.72				
4 weeks	82.77	0.60	0.00	2.85	4.28	4.18	5.32				
6 weeks	83.33	1.41	0.00	2.23	5.01	3.37	4.56				
10 weeks	55.93	15.16	1.63	2.90	8.51	1.28	14.60				
16 weeks	81.24	4.87	0.79	4.18	3.63	1.35	3.94				

TABLE 16: Chemical analysis results of samples subjected to static testing at different time intervals.

Time	O	Na	Mg	Si	P	Ag	Ca	Mo	Cr	Fe	Ni
Substrate								2.11	18.75	65.22	13.92
0 h	56.19	7.77	3.09	13.70	9.17	2.60	7.48				
12 h	51.47	8.65	4.92	18.34	6.34	1.94	8.34				
24 h	61.21	6.09	1.96	13.71	6.72	1.82	8.49				
1 week	65.79	5.33	2.55	13.64	5.25	3.49	3.94				
2 weeks	74.37	5.04	0.74	8.91	6.12	3.93	0.88				
3 weeks	75.78	4.95	0.01	4.07	5.88	1.24	8.05				
4 weeks	80.12	5.26	0.27	8.32	3.07	0.23	2.16				
6 weeks	76.63	4.50	0.10	6.74	4.11	3.98	2.30				
8 weeks	63.64	7.91	0.50	3.00	9.34	4.57	10.73				
10 weeks	73.33	8.95	1.00	6.75	5.82	1.87	2.28				
16 weeks	69.94	12.95	2.49	6.42	2.60	3.15	2.45				

TABLE 17: Chemical analysis results of samples subjected to dynamic testing at different time intervals.

Sample	Element concentration (mg/g) / Wavelength (nm)				
	Mg 285.213	Ca 317.933	Na 589.592	P 214.914	K 766.490
Initial	0.112	1.027	29.43	0.979	1.110
12 h	0.249	2.421	28.30	1.140	1.142
24 h	0.120	2.222	29.50	0.974	0.150
1 week	0.178	2.160	28.03	1.000	1.068
2 weeks	0.624	5.173	34.06	1.160	1.810
3 weeks	0.153	1.125	26.00	0.889	0.936
4 weeks	0.099	1.310	30.94	1.047	1.209
6 weeks	0.374	4.732	29.99	1.086	1.247
8 weeks	0.384	3.355	29.95	1.146	1.475
10 weeks	0.161	2.439	28.71	1.021	1.357

TABLE 18: Changes in element concentrations in plasma solution after static testing over time

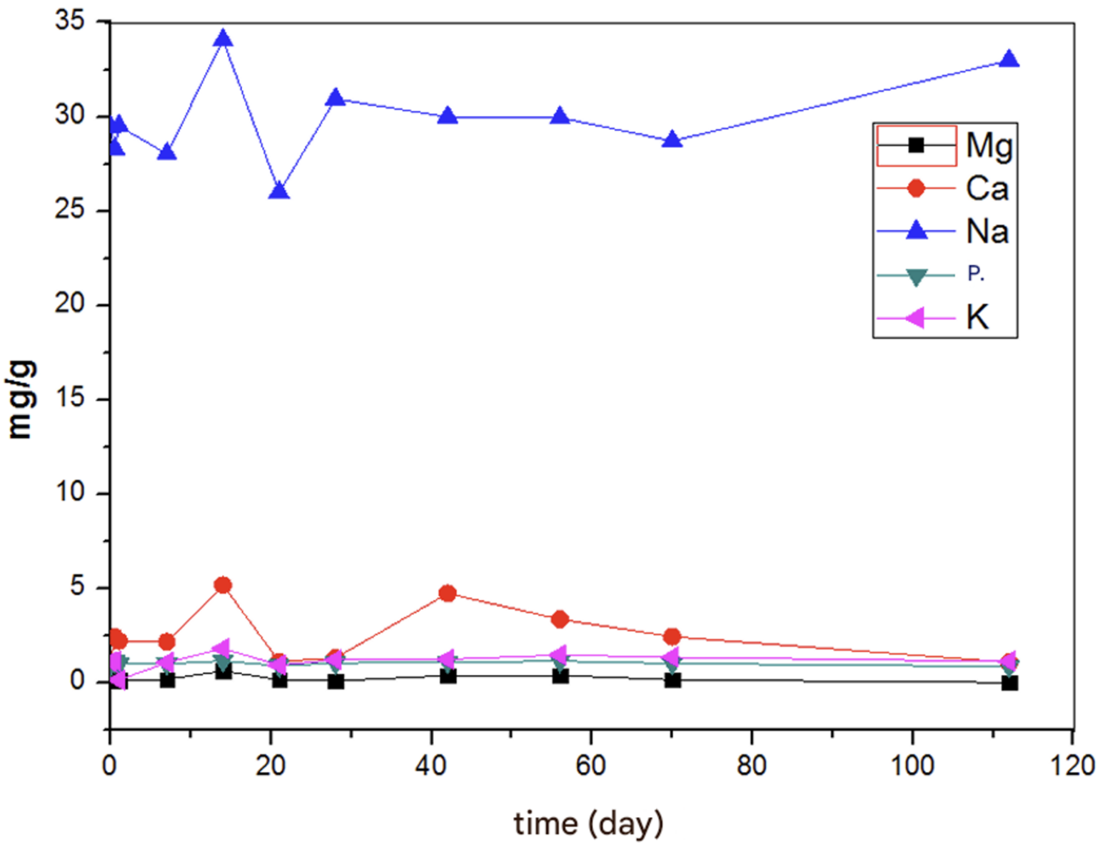

FIGURE 71: Time-dependent change of elements in plasma liquid.

Figure 72 Scanning electron microscopy (SEM) images showing the microstructural evolution of hydroxyapatite (HAP) in simulated body fluid (SBF) at different time intervals. At the initial stage (a), the surface exhibited well-defined polygonal grains with smooth boundaries. After 7 days, the formation of apatite nodules became evident, indicating the onset of bioactive mineral deposition. At 14 days, localized dissolution zones were observed alongside newly precipitated apatite clusters, reflecting simultaneous degradation and mineralization processes. By 21 days, the surface displayed a more heterogeneous microstructure with extensive apatite formation and partial surface roughening, confirming the long-term bioactivity and dynamic remodeling behavior of the coating in a physiological environment.

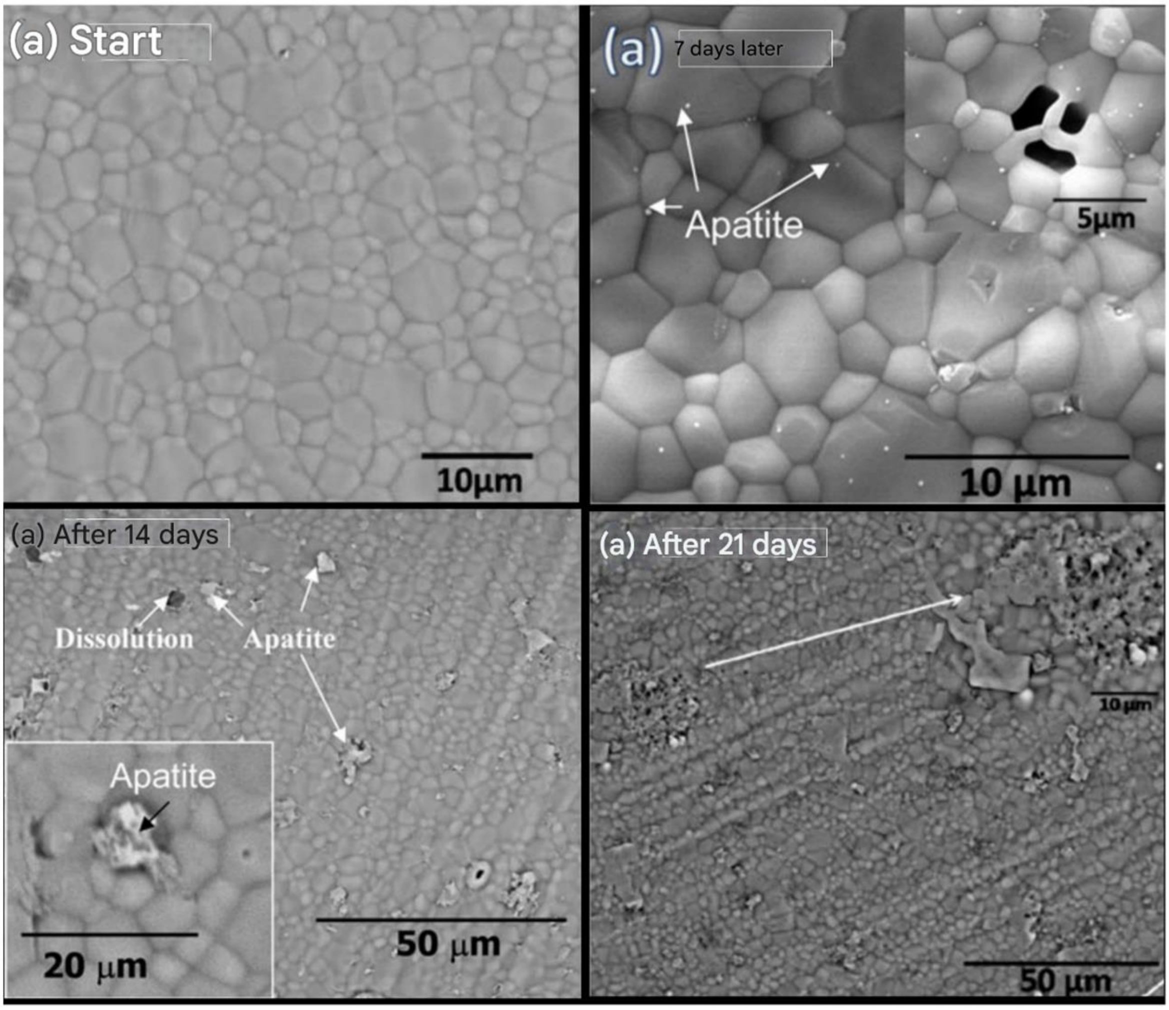

FIGURE 72: Changes in the microstructure of HAP with time in artificial body fluid

In summary, both static and dynamic dissolution experiments confirmed the stability and durability of the coatings. The minimal mass loss, consistent elemental composition, and absence of delamination supported their long-term structural integrity. Simultaneously, the controlled release of silver provided sustained antibacterial potential, while retention of calcium and phosphorus ensured osteoconductive properties. These findings validated the coatings as suitable for clinical applications requiring both mechanical robustness and biological functionality.

6. Post-Clinical Characterization of Coated Implants

Retrieved implants were analyzed to assess the stability of silver-ion–doped calcium phosphate coatings after surgical application. Optical inspection of multiple patient samples confirmed that the coatings remained adherent and continuous, with only minor polishing visible in areas subjected to mechanical stress (Figure 73). SEM surface examinations demonstrated coating continuity and adhesion without evidence of delamination (Figure 74). Complementary elemental analysis by EDX at the implant–bone interface confirmed the presence of calcium, phosphorus, and oxygen, indicating preservation of the coating composition without significant degradation (Figure 75).

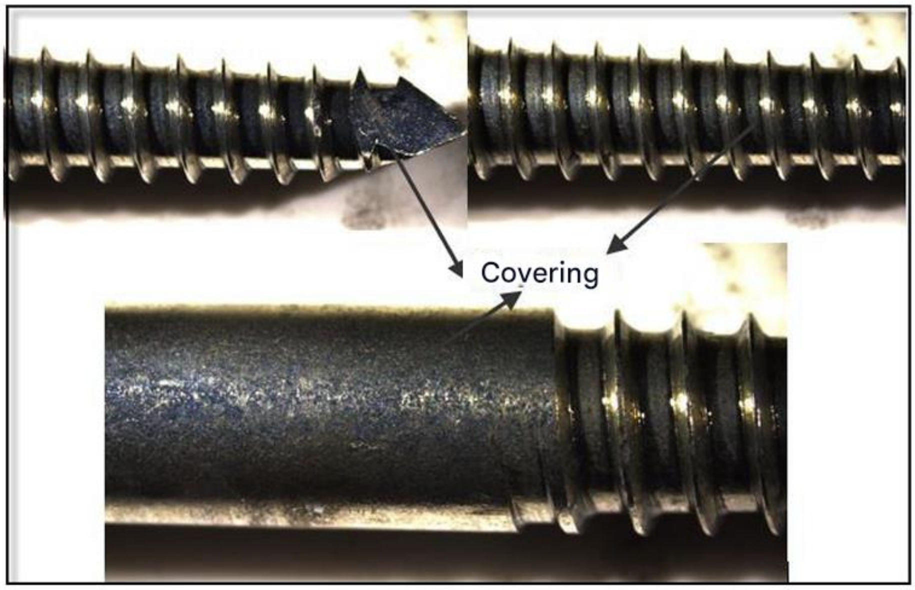

(a)

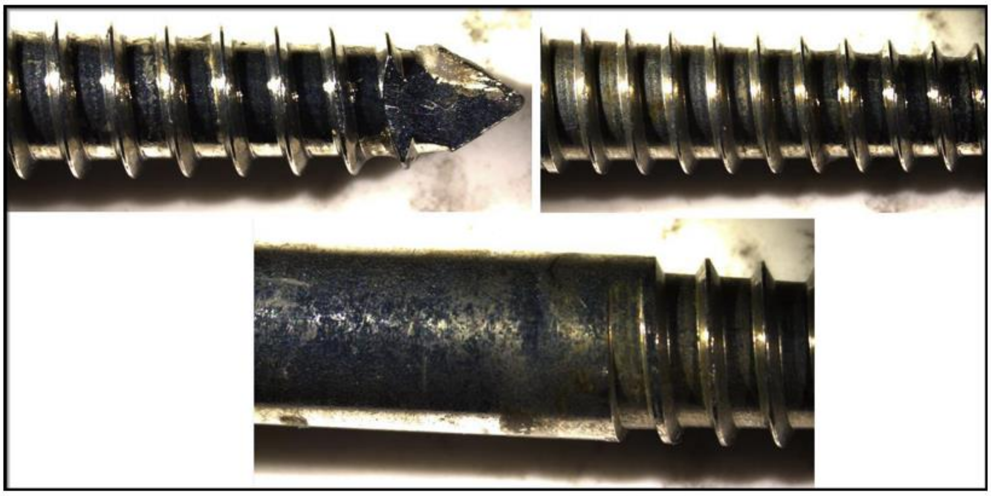

(b)

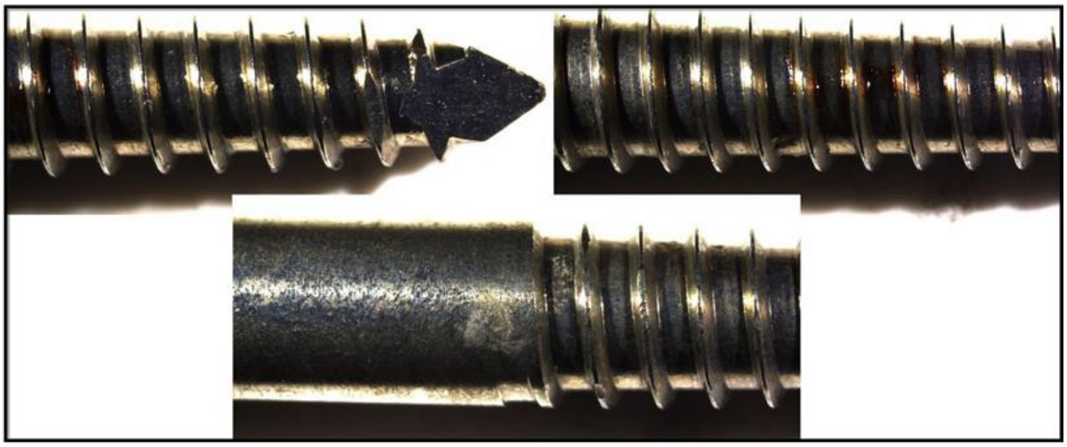

(c)

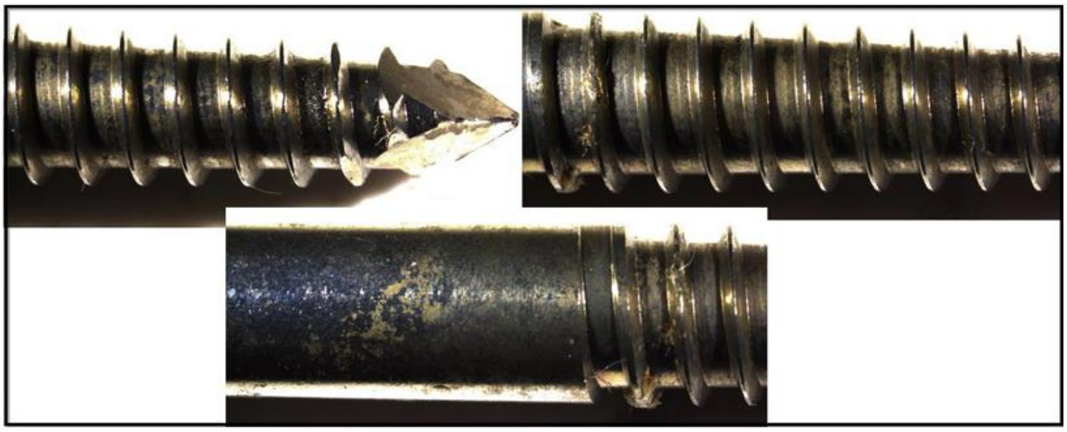

(d)

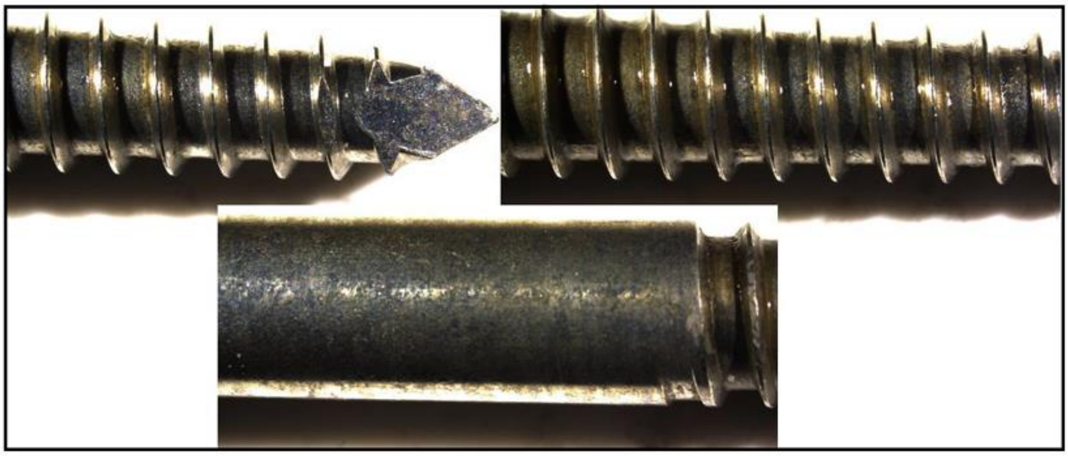

(e)

FIGURE 73: Optical surface images of implants.

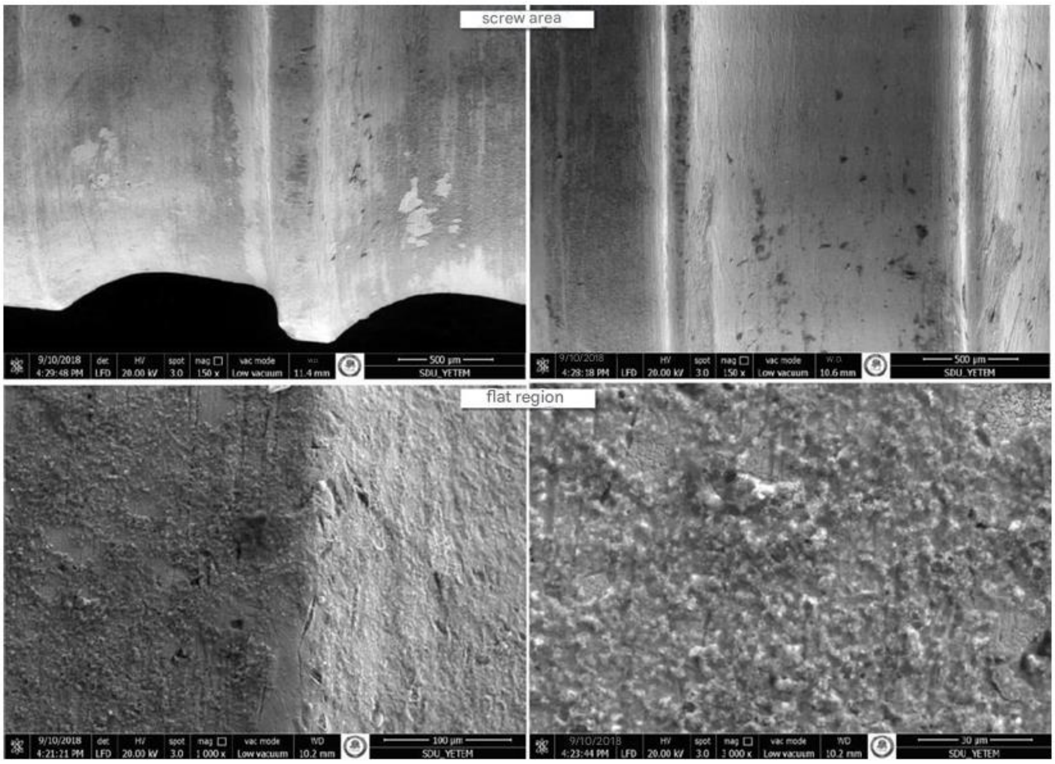
screw area
flat region
500 μm
100 μm
30 μm
SDU_YETEM

(a)

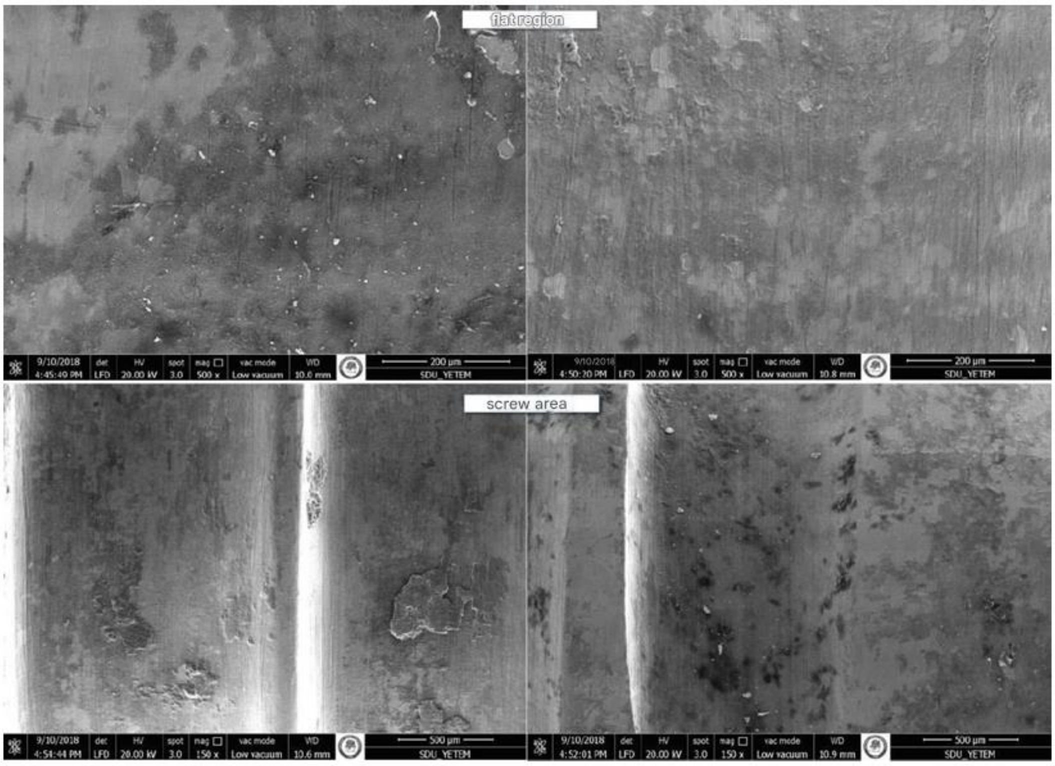
flat region
screw area
200 μm
500 μm
SDU_YETEM

(b)

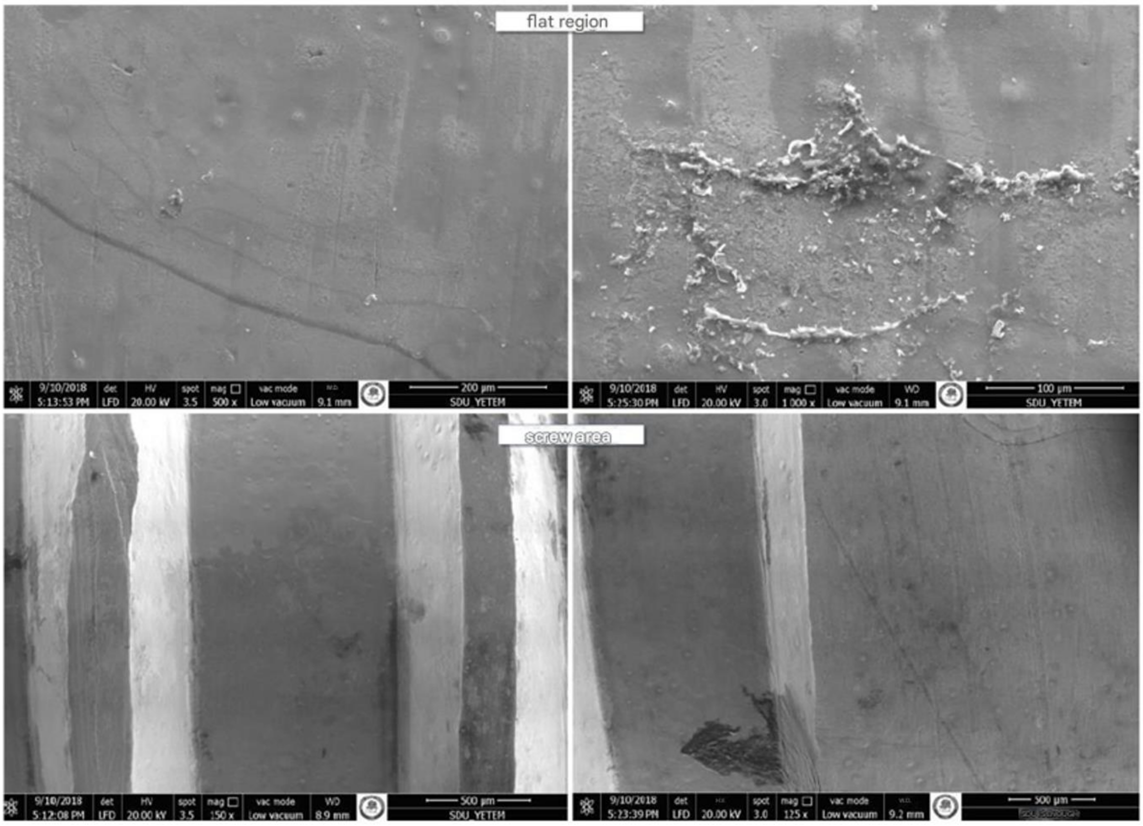

(c)

FIGURE 74: SEM surface morphology.

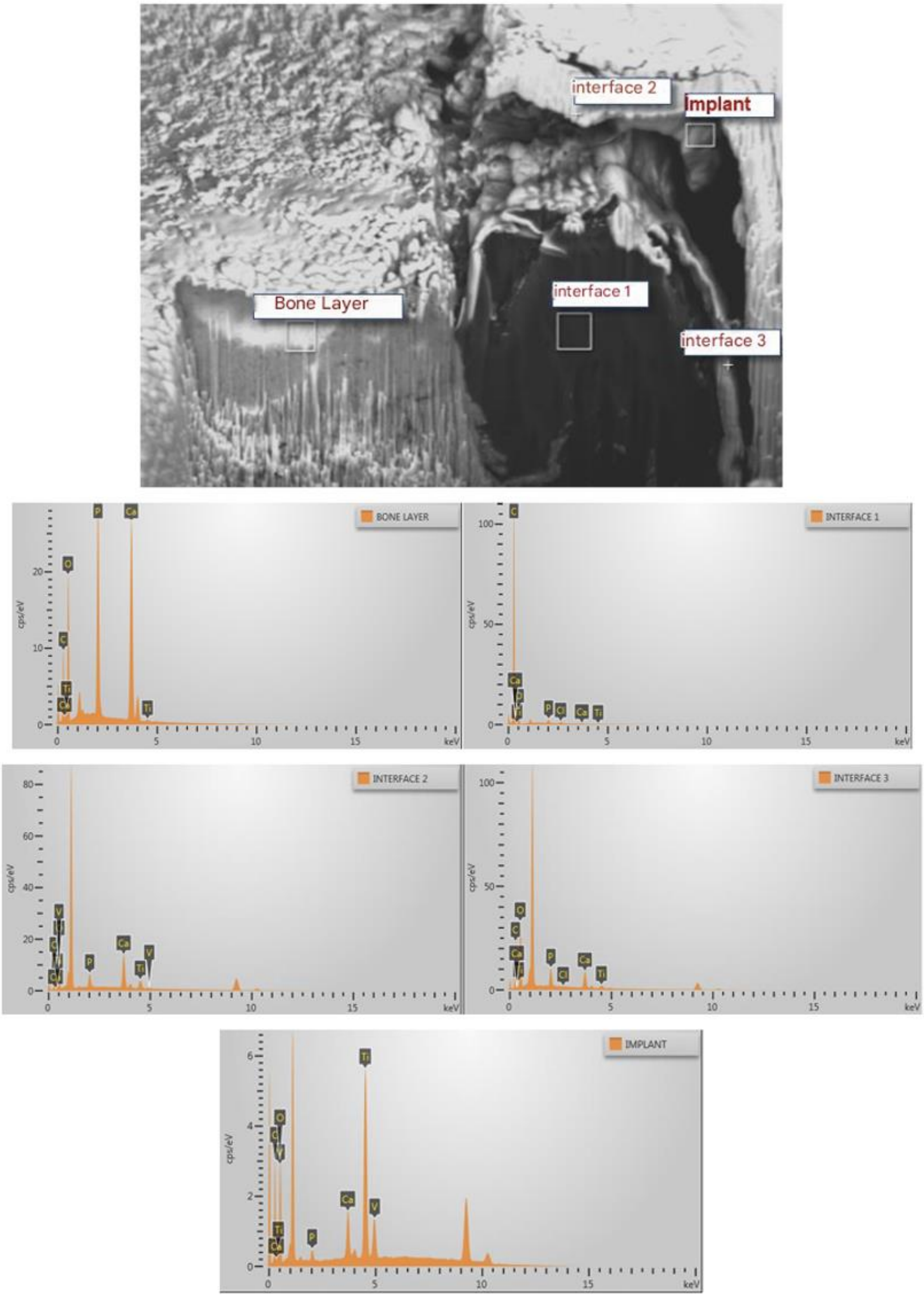

FIGURE 75: EDX chemical analysis.
