# Supplementary material for: Safety and Preliminary Clinical Outcomes of Silver Ion-Doped Calcium Phosphate-Coated Orthopedic Implants: A Prospective Pilot Study
Source: J Funct Biomater. 2026 Oct 15;17(10):517. doi: 10.3390/jfb17100517 (PMC13706277; doi:10.3390/jfb17100517)
Supplement: Supplementary file 1 [file jfb-17-00517-s001.zip › supplementary file S2 medical specialty thesis.pdf]

SUPPLEMENTARY FILE S2

Detailed Clinical Laboratory and Culture Data

This file contains the extended follow-up data of the study cohort, including serial biochemical laboratory results (ALT, AST, BUN, creatinine, ESR, and CRP) by implant subgroup and overall, plus microbiological culture results. Time points: Preoperative, 1 month, 3 months, 6 months, and Final. Data are presented as mean ± SD (min–max) as reported in the thesis.

Groups: External Fixator (ExFix, n=14), Proximal Femoral Nail (PFN, n=10), Partial Arthroplasty (n=6), Total (n=30).

Serial measurements of ALT and AST across all groups showed no significant deterioration during follow-up. Mild fluctuations observed in the early postoperative period remained within clinically acceptable ranges. Overall, hepatic function was preserved throughout the study.

**Time**	**ExFix (n=14)**	**PFN (n=10)**	**Partial Arthroplasty (n=6)**	**Total (n=30)**
Preop	27.43 ± 14.55 (16.75–35.75)	25.14 ± 9.06 (18.00–32.00)	21.40 ± 4.04 (18.00–25.50)	25.65 ± 11.74 (17.75–29.75)
1 mo	21.71 ± 7.88 (16.75–26.75)	17.57 ± 9.20 (12.00–20.00)	17.80 ± 7.60 (11.00–24.50)	19.85 ± 8.13 (14.50–23.75)
3 mo	23.29 ± 13.84 (14.00–27.75)	18.60 ± 7.73 (12.50–27.00)	13.60 ± 5.13 (10.00–18.50)	20.29 ± 11.79 (13.25–25.50)
6 mo	18.86 ± 7.07 (14.75–20.50)	11.80 ± 4.44 (7.00–15.50)	15.00 ± 7.68 (9.00–23.00)	16.58 ± 7.11 (12.00–18.75)
Final	17.00 ± 3.46 (14.00–20.25)	14.80 ± 5.89 (10.00–19.50)	12.00 ± 4.83 (8.50–17.00)	15.65 ± 4.51 (12.00–19.00)

TABLE 1 ALT results (mean ± SD, min–max)

Time	ExFix	PFN	Partial Arthroplasty	Total
Preop	20.07 ± 7.08 (14.00–27.25)	20.00 ± 14.66 (11.00–28.00)	12.00 ± 3.87 (8.50–16.00)	18.50 ± 9.51 (12.50–25.50)
1 mo	28.00 ± 13.65 (18.50–37.75)	9.43 ± 2.99 (7.00–12.00)	15.20 ± 15.45 (5.00–27.00)	20.54 ± 14.45 (9.75–28.25)
3 mo	27.00 ± 15.89 (16.00–37.75)	8.60 ± 4.34 (5.50–12.00)	10.40 ± 6.88 (5.50–16.00)	19.71 ± 15.24 (8.00–23.75)
6 mo	22.79 ± 15.09 (14.00–30.50)	9.40 ± 2.97 (7.00–12.00)	15.20 ± 10.55 (5.00–24.50)	18.42 ± 13.45 (9.25–23.75)
Final	18.86 ± 6.60 (13.25–26.00)	11.20 ± 3.70 (8.00–15.00)	11.25 ± 9.91 (5.25–21.50)	15.87 ± 7.49 (10.00–21.00)

TABLE 2 AST results (mean ± SD, min–max)

Renal Function Tests (BUN, Creatinine):BUN and creatinine values demonstrated stability at all time points. Group comparisons revealed no progressive increases suggestive of renal impairment. These findings indicate that renal function remained stable during the entire follow-up period.

Time	ExFix	PFN	Partial Arthroplasty	Total
Preop	15.62 ± 7.18 (10.53–19.25)	26.00 ± 11.76 (17.00–37.00)	14.28 ± 7.85 (9.45–20.75)	18.16 ± 9.68 (10.93–20.50)
1 mo	13.31 ± 2.99 (10.98–15.25)	22.77 ± 7.58 (15.00–31.00)	14.60 ± 6.07 (9.00–20.50)	16.10 ± 6.45 (12.13–19.10)
3 mo	12.71 ± 3.57 (10.00–14.38)	19.60 ± 5.73 (14.50–25.00)	17.54 ± 4.88 (13.25–22.60)	15.15 ± 5.12 (11.00–19.00)
6 mo	13.45 ± 4.19 (10.00–18.25)	20.52 ± 5.62 (15.80–26.00)	14.72 ± 4.35 (11.15–18.65)	15.19 ± 5.17 (11.00–19.00)
Final	13.57 ± 2.98 (11.00–15.00)	19.64 ± 8.26 (13.35–25.75)	18.48 ± 4.78 (14.28–23.35)	15.74 ± 5.35 (12.00–19.00)

TABLE 3: BUN results (mean ± SD, min–max)

Time	ExFix	PFN	Partial Arthroplasty	Total
Preop	0.88 ± 0.33 (0.65–0.98)	0.91 ± 0.36 (0.58–1.08)	0.82 ± 0.29 (0.60–1.13)	0.88 ± 0.32 (0.62–1.03)
1 mo	0.89 ± 0.32 (0.72–0.94)	0.91 ± 0.28 (0.63–1.16)	0.75 ± 0.28 (0.46–1.00)	0.87 ± 0.30 (0.69–1.02)
3 mo	0.82 ± 0.16 (0.72–0.90)	0.84 ± 0.26 (0.62–1.05)	0.75 ± 0.30 (0.47–1.06)	0.81 ± 0.21 (0.70–0.97)
6 mo	0.83 ± 0.18 (0.69–0.99)	0.89 ± 0.29 (0.65–1.15)	0.74 ± 0.21 (0.54–0.94)	0.82 ± 0.21 (0.67–0.97)
Final	0.87 ± 0.17 (0.78–0.95)	1.02 ± 0.28 (0.78–1.32)	0.80 ± 0.16 (0.63–0.93)	0.89 ± 0.20 (0.79–0.96)

TABLE 4: Creatinine results (mean ± SD, min–max)

Inflammatory Markers (ESR, CRP): Both ESR and CRP levels were elevated preoperatively in several patients, reflecting the baseline clinical status. However, a progressive decline was observed during follow-up, with values approaching or reaching near-normal ranges by the final visit. This trend is consistent with the resolution of the inflammatory response after surgery.

Time	ExFix	PFN	Partial Arthroplasty	Total
Preop	29.79 ± 26.11 (10.50–55.25)	73.14 ± 33.20 (47.00–105.00)	40.40 ± 23.74 (21.00–60.50)	43.50 ± 32.60 (16.25–68.50)
1 mo	35.57 ± 33.35 (15.00–61.25)	82.00 ± 11.22 (75.00–86.00)	56.00 ± 20.41 (37.50–71.00)	52.00 ± 32.89 (20.25–75.50)
3 mo	29.64 ± 25.64 (8.75–47.25)	48.00 ± 13.00 (36.00–58.50)	40.40 ± 28.58 (16.00–65.00)	35.71 ± 24.56 (9.75–52.50)
6 mo	24.21 ± 18.45 (10.50–39.00)	47.40 ± 22.90 (29.00–67.50)	39.80 ± 35.46 (13.00–68.50)	32.29 ± 24.57 (13.25–43.50)
Final	18.29 ± 17.29 (6.75–30.75)	47.40 ± 26.39 (18.50–67.00)	44.75 ± 27.99 (16.00–67.75)	29.22 ± 24.60 (8.00–55.00)

TABLE 5: ESR (sedimentation) results (mean ± SD, min–max)

Time	ExFix	PFN	Partial Arthroplasty	Total
Preop	11.54 ± 9.95 (2.23–23.30)	33.77 ± 26.60 (12.80–68.00)	37.80 ± 55.56 (6.00–84.25)	22.57 ± 29.41 (4.58–25.63)

Time	ExFix	PFN	Partial Arthroplasty	Total
1 mo	11.13 ± 12.87 (3.13–14.90)	18.90 ± 7.01 (14.00–19.80)	36.42 ± 35.31 (7.95–66.35)	18.08 ± 19.80 (4.78–23.23)
3 mo	12.96 ± 20.37 (2.75–8.90)	12.40 ± 12.14 (5.00–21.60)	14.36 ± 11.77 (4.65–25.75)	13.13 ± 16.88 (3.40–10.70)
6 mo	8.49 ± 11.09 (3.25–7.70)	8.84 ± 5.67 (4.75–14.15)	7.37 ± 5.66 (2.98–13.55)	8.33 ± 9.00 (3.43–9.88)
Final	7.05 ± 13.27 (1.98–4.50)	6.20 ± 4.04 (2.75–9.95)	9.18 ± 5.50 (3.38–12.95)	7.23 ± 10.59 (2.10–9.90)

TABLE 6: CRP results (mean ± SD, min–max)

Silver (Ag) Measurements: Whole-blood silver levels were consistently measured below 0.34 µg/mL at all time points, indicating that systemic silver accumulation did not occur. This supports the systemic safety of silver-ion–doped calcium phosphate coatings.

Microbiological Culture Results: Among the retrieved samples, *Staphylococcus epidermidis* was the most frequently isolated microorganism (21.9%), followed by *S. aureus* (9.8%). Notably, no bacterial growth was detected in 40.6% of cases, supporting the antibacterial effectiveness of the coating and perioperative protocols.

Microorganism	n	%
Staphylococcus epidermidis	20	21.9
Normal skin flora	9	9.8
Staphylococcus aureus	9	9.8
Staphylococcus lugdunensis	6	6.6
Klebsiella spp.	3	3.3
Other (e.g., *S. hominis, P. mirabilis*, etc.)	7	7.6
No growth	37	40.6

TABLE 7: Microbiological culture results
